# Novel centromeric plasmid for stable extrachromosomal gene expression in *Aurantiochytrium limacinum*

**DOI:** 10.1007/s00253-025-13527-w

**Published:** 2025-07-04

**Authors:** Person Pesona Renta, Cian-Huei Syu, Ta-Yu Huang, Yi-Ting Chang, Yu-Feng Liang, Ssu-Ting Chen, Po-Wei Weng, Ming-Chen Hsu, Keng-Hung Lin, Tsunglin Liu, Anna C.-C. Jang, Che-Chia Tsao, Han-Jia Lin, Hung-Yun Lin, Yi-Min Chen

**Affiliations:** 1 Department of Biotechnology and Bioindustry Sciences, National Cheng Kung University, Tainan, 701401 Taiwan; 2 Department of Bioscience and Biotechnology, National Taiwan Ocean University, Keelung, 202301 Taiwan; 3 Center of Excellence for the Oceans, National Taiwan Ocean University, Keelung, 202301 Taiwan; 4 Department of Biological Sciences and Technology, National University of Tainan, Tainan, 700301 Taiwan

**Keywords:** *Aurantiochytrium limacinum*, Centromeric plasmid, Genetic manipulation, Shuttle vector

## Abstract

**Abstract:**

*Aurantiochytrium* *limacinum* holds great promise for producing sustainable single-cell oil as an alternative to fish oil. However, research into its complex biological and biochemical characteristics and efforts toward strain improvement have been hampered by insufficient genetic tools. Until now, genetic transformations of *A. limacinum* have relied solely on chromosome integration, which is inefficient and prone to insertional mutagenesis and other issues related to genetically modified organisms (GMOs). This paper describes the first centromeric plasmid for *A. limacinum*. Amplification of this shuttle vector by *E. coli* enables direct delivery into *A. limacinum* via electroporation, where it undergoes stable replication and segregation into daughter cells. The key to the stable plasmid maintenance lies in a 500 bp segment derived from chromosome 24 of *Phaeodactylum tricornutum*. While this segment does not significantly enhance the efficiency of vector transformation, it enables the replication and maintenance of the shuttle vector in the host cell as closed circular DNA. The plasmid from three transformants demonstrates a high segregation efficiency of 96.8 ± 0.3% (*n* = 3), even in the absence of antibiotic selection. This novel centromeric plasmid considerably enhances the flexibility of genetic manipulations and gene expression in *A. limacinum*, opening new avenues for its study and industrial application.

**Key points:**

*• First centromeric plasmid developed for genetic transformation in A. limacinum.*

*• The novel plasmid enhances flexibility in genetic manipulation and gene expression.*

*• The plasmid achieves 96.8 ± 0.3% (n = 3) segregation efficiency without antibiotic selection.*

**Supplementary Information:**

The online version contains supplementary material available at 10.1007/s00253-025-13527-w.

## Introduction

Global aquaculture production surpassed wild marine capture fisheries for the first time in 2022 (Fisheries 2022). However, paradoxically, its sustainability still relies on marine-derived feed. Researchers have had considerable success in developing alternative protein sources for fishmeal, including plant proteins, insect meal, and microbial biomass (Jannathulla et al. 2019); however, efforts to replace fish oil with nutritionally equivalent alternatives have proven more challenging. Current microbial sources of omega-3 fatty acids, such as *Crypthecodinium cohnii* and the thraustochytrid *Aurantiochytrium* (formerly *Schizochytrium*) *limacinum*, produce oils with imbalanced fatty acid profiles (DHA-rich but EPA-deficient), and at costs far exceeding those of conventional fish oil (Abad and Turon 2015; Mendes et al. 2009). There is a pressing need for innovative solutions to achieve truly sustainable aquaculture without compromising the integrity of marine ecosystems (Benzie et al. 2010; Bush et al. 2013).

Researchers have developed specialized genetic transformation tools for *A. limacinum* to reduce production costs and improving the fatty acid composition by enhancing DHA yield (Rau and Ertesvåg 2021; Watanabe et al. 2021) or introducing EPA into *A. limacinum* oil through genetic/metabolic engineering (Wang et al. 2020). The genetic transformation tools specific to *A. limacinum* employ techniques, such as electroporation or gene gun bombardment, deliver expression cassettes housed on linear or circular vectors into the host cell nucleus. This allows integration of the cassettes into the chromosome via random insertion or homologous recombination at targeted loci. However, these methods often lack efficiency and are susceptible to insertional mutagenesis and other challenges associated with genetically modified organisms (GMOs) (Watanabe et al. 2021).

Centromeric (or centromere) plasmids are replicating vectors used for gene transformation in yeast (Cao et al. 2017; Gnügge and Rudolf 2017; Piva et al. 2020; Taxis and Knop 2006) and *Plasmodium falciparum* (Iwanaga et al. 2012; Payungwoung et al. 2018). They contain an autonomously replicating sequence (ARS) and a centromeric DNA sequence (CEN), enabling independent replication within the host nucleus without the need for integration into the chromosome. Centromeric plasmids that enter the cell nucleus typically replicate in synchrony with chromosomes and maintain a copy number equivalent to that of chromosomes. After replication, the plasmids are evenly distributed between the two daughter cells. For eukaryotic cell transformations, centromeric plasmids offer several advantages over targeted or random DNA insertion methods: (1) They can be applied as shuttle vectors, enabling seamless transfer between prokaryotic and eukaryotic systems to facilitate preparation and transformation. (2) They provide higher transformation efficiency. (3) They minimize the risk of insertional mutagenesis. (4) They can be conveniently eliminated simply by removing selection pressure, addressing many concerns associated with genetically modified organisms (GMOs) (Cao et al. 2017; Piva et al. 2020; Van Craenenbroeck et al. 2000). Moreover, these vectors are particularly suitable for gene editing applications requiring “long-term” transient expression (Kurita et al. 2022; Payungwoung et al. 2018).

*A. limacinum* strain BL10 (referred to as BL10) is a promising thraustochytrid strain for the industrial-scale production of single-cell oil (Chaung et al. 2012; Yang et al. 2010). Our objective in this study was to develop transformation tools for DNA insertion and foreign gene expression with the aim of enhancing the DHA yield of BL10 through genetic/metabolic engineering. The design of these tools was informed by existing genetic transformation methods developed for thraustochytrids (Cheng et al. 2011; Dahmen et al. 2023; Ma et al. 2021; Rau and Ertesvåg 2021; Sakaguchi et al. 2012; Sun et al. 2015; Watanabe et al. 2021) and the heterokont alga *Phaeodactylum tricornutum* (Diner et al. 2016, 2017; Karas et al. 2015; Moosburner et al. 2020). Various linear and circular DNA constructs were designed and tested for their transformation efficiency. Results showed that incorporating the plasmid maintenance sequence CEN6-ARSH4 (CA) derived from the replicative vector of *Saccharomyces cerevisiae* (Karas et al. 2015) into circular DNA constructs significantly improved transformation efficiency. This improvement can be partially attributed to the fact that this sequence enables the circular vector to replicate autonomously and exist as extrachromosomal DNA in BL10 host cells. Replacing the CA sequence with a centromeric-like DNA sequence from *P. tricornutum* as an alternative plasmid maintenance sequence further stabilized these circular DNA constructs as extrachromosomal DNA in BL10. The development of this novel centromeric vector represents a significant advancement, enhancing the flexibility of genetic manipulation and gene expression in *A. limacinum*.

## Materials and methods

### Preparation of DNA constructs for transformation of BL10 cells

#### Preparation of circular constructs

Construction of the pE0EE plasmid involved three steps: (1) amplifying the *ef1α* gene (from promoter to terminator) of BL10 using primer sets ef1-(−1772)-*Not*I (F) and ef1-2229-*Spe*I (R) with genomic DNA obtained using established methods (Wu et al. 2024); (2) amplifying partial sequences of the T&A vector using primer sets T&A-495-*Spe*I (F) and T&A-170-*Not*I (R), with a T&A vector obtained using a T&A Cloning Kit provided by Yeasen Biotechnology (Gaithersburg, MD, USA); and (3) joining the two fragments into a circular structure through restriction digest (using *Not*I and *Spe*I) and ligation.

Construction of the pE1 NE plasmid involved three steps: (1) replacing the *ef1α* coding sequence in pE1EE with the coding sequence of the neomycin resistance (NeoR) gene, which involved amplifying the coding sequence of neoR from the pEGFP-N1 vector (Addgene, Watertown, MA, USA) using the primer sets N1-2629-*Nde*I (F) and N1-3423-*Bam*HI (R); (2) amplifying a partial sequence of the pE1EE plasmid using the primer sets E1EE-2783-*Bam*HI (F) and E1EE-1780-*Nde*I (R); and (3) joining the two fragments into a circular structure through restriction digest (using *Nde*I and *Bam*HI) and ligation.

Construction of the pE2 NE plasmid involved the following steps: (1) replacing the promoter region of *ef1α* in pE1 NE (E1) with the shorter E2 promoter, which lacks a 301 bp “small open reading frame” sequence near 3′-end of E1. This replacement was achieved by amplifying the E2 sequence from pE1 NE using the primer set E1 NE-1-*Not*I (F) and E1 NE-1478-*Nde*I (R); (2) replacing the E1 promoter in pE1 NE with E2 via restriction digest (using *Not*I and *Nde*I) and ligation.

Construction of the pE1 NG1 and pE2 NG1 plasmids involved three steps: (1) replacing the terminator region of the *ef1α* (E) in pE1 NE and pE2 NE with the terminator region of the glyceraldehyde-3-phosphate dehydrogenase (GAPDH) gene (G1) of BL10; (2) amplifying G1 using the primer sets gadph-t-1-*Bam*HI (F) and gadph-t-978-*Spe*I (R) from genomic DNA of BL10; (3) replacing the E terminator in pE1 NE and pE2 NE with G1 via restriction digest (using *Bam*HI and *Spe*I) and ligation.

The construction of the p18S plasmid involved three steps: (1) amplifying two partial sequences located near the 5′ and 3′ ends of the 18S rRNA gene of BL10 using the primer sets 18S-124-*Nde*I (F) and 18S-566-*Nde*I (R), and 18S-NKNS-934 (F) and 18S-*Bam*HI-1536 (R); (2) obtaining a partial sequence of the T&A vector using the primer sets T&A-457-*Bam*HI (F) and T&A-182-*Nde*I (R); and (3) joining the three fragments into a circular structure through restriction digest (using *Bam*HI and *Nde*I) and ligation.

Construction of the pE1 NE-18S, pE2 NE-18S, pE1 NG1-18S, and pE2 NG1-18S plasmids involved inserting the expression cassette from either pE1 NE, pE2 NE, pE1 NG1, or pE2 NG1 plasmids into the p18S plasmid through restriction digest (using *Not*I and *Spe*I) and ligation. The pE1 NE-18S-CA, pE2 NE-18S-CA, pE1 NG1-18S-CA, and pE2 NG1-18S-CA plasmids were constructed by incorporating the Cen-Ars sequence amplified from the pPtPuc3 vector (Addgene, Watertown, MA, USA) using the primer sets Puc3-2479-*Nde*I (F) and Puc3-3144-*No*tI (R) into the pE1 NE-18S, pE2 NE-18S, pE1 NG1-18S, and pE2 NG1-18S plasmids through restriction digest (using *Nde*I and *No*tI) and ligation.

Construction of the pUNG1-18S plasmid involved replacing the E1 promoter in pE1 NG1-18S with the promoter region of the ubiquitin gene of BL10 through restriction digest (using *Nde*I and *Not*I) and ligation. The ubiquitin gene was amplified using the primer sets ubi-(−762)-*Not*I (F) and ubi-(−1)-*Nde*I (R). pUNG2 plasmid construction involved inserting a partial sequence of pUNG1-18S amplified using the primer set UNG1-18S-641-*Not*I (F) and UNG1-18S-2505-*Spe*I (R) into the T&A vector via restriction digestion (using *Spe*I and *Not*I) and ligation. Unlike the G1 terminator in pUNG1-18S, the G2 terminator in pUNG2 lacks a 648 bp region on the G1 at the 3′-end. The pUNG2-CA plasmid was constructed by introducing the CA sequence from pE1 NG1-18S-CA into the pUNG2 plasmid through restriction digest (using *Kpn*I and *Not*I) and ligation. The CA sequence was amplified using the primer set E1 NG1-18S-633-*Kpn*I (F) and E1 NG1-18S-1298-*Not*I (R).

Construction of the pUNG2-PIT-24, −29, and −30 plasmids involved replacing the CA sequence in the pUNG2-CA plasmid with predicted centromeric-like sequences from chromosomes 24, 29, and 30 of *Phaeodactylum tricornutum*. The process began with the prediction of centromeric-like sequences in *P. tricornutum* using the genome database (NCBI dataset GCA_000150955.2). This involved analyzing 100-bp windows (overlapping by 50 bp) with a GC content of 32% or lower within a 3-kb sliding window, advancing in 1-kb steps. The 3-kb region with the highest number of such 100-bp windows was then selected for further analysis to identify the 500-bp sequence with the lowest GC content within this region, based on 500-bp windows overlapping by 250 bp (Diner et al. 2017). Three of the predicted 500-bp centromeric-like sequences with lowest GC content were selected for synthesis (with added *Kpn*I and *Not*I restriction enzyme sites) by MingXin Biotech (Taipei, Taiwan). The synthesized sequences were then inserted into pUNG2 via restriction digest (using *Kpn*I and *Not*I) and ligation.

Pfu DNA Polymerase (Promega, Madison, WI, USA) was used for high-fidelity PCR to amplify desired partial sequences from plasmids or genomic DNA. The PCR conditions for the construction of circular vectors are specified in Supplemental Table S1. Maps and sequences of the circular vectors are shown in Supplemental Figure S1. The plasmid construction workflow is detailed in Supplemental Figure S2.

#### Preparation of linear constructs

Linear constructs, including L-E1 NE-18S, L-E2 NE-18S, L-E1 NG1-18S, and L-E2 NG1-18S, were generated through PCR amplification from their corresponding circular plasmids: pE1 NE-18S, pE2 NE-18S, pE1 NG1-18S, and pE2 NG1-18S. Amplification was performed using the KAPA LongRange HotStart ReadyMix PCR Kit (Kapa Biosystems, Cape Town, South Africa) under the conditions specified in Supplemental Table S1.

### Transformation of BL10 cells

The BL10 strain used in this study was an axenic strain collected from a mangrove forest near Taipei in 2007 (Yang et al. 2010). This strain has been deposited in the Bioresource Collection and Research Center (BCRC) in Taiwan, with the strain number BCRC 980009. Following purification, specimens were cryopreserved at − 80 °C in accordance with the methods in our previous publication (Chou et al. 2022).

BL10 activation involved the rapid thawing of cryopreserved cell stock in a water bath at 37 °C, after which 50 µL of the stock was transferred into a 50-mL glass culture tube containing 5 mL of H3 medium (Yang et al. 2010) (Supplemental Table S2). The culture was then incubated at 27 °C in a shaker (150 rpm) for 24 h.

Following activation, 50-µL aliquots of the seed culture were transferred into 50-mL test tubes containing 5 mL of 2 × GYSS medium (Supplemental Table S2), consisting of sodium sulfate/yeast extract/glucose (9/9/90 g/L) in 1‰ diluted natural seawater. The cultures underwent agitation in a shaker (150 rpm) at 27 °C for 11.5 h. At this point, the amoeboid cells constituted the highest proportion of the culture, following the transformation of 20–30% of vegetative cells into cell wall-deficient amoeboid cells (Chou et al. 2022). A 1.5-mL aliquot of the culture was harvested, centrifuged at 4000 × g for 5 min to remove the medium, and rinsed twice with 1 mL of 1 M sorbitol aqueous solution. The cells were then resuspended in 200 μL of 1 M sorbitol aqueous solution and gently mixed with 30 μL of DNA aqueous solution containing the desired DNA constructs (1.94 ng/bp). Electroporation was then performed in a 1-mm gap cuvette using a single 500-V pulse for 5 ms utilizing the ECM 399 exponential decay wave electroporation system (BTX, Holliston, MA, USA).

Following electroporation, the contents of the cuvette were transferred into a 1.5-mL Eppendorf tube containing 500 μL of 2 × GYSS medium and recovered in a shaker (150 rpm) at 27 °C for 4 h. The cells were harvested via centrifugation (4000 × g, 5 min at 4 °C) and resuspended in fresh 2 × GYSS medium at a density of approximately 10⁷ cells/100 µL. Using a triangle-ended glass spreader, the resuspended cells were spread evenly on G418 agar plates (15 mL of M3 medium (Supplemental Table S2) with 0.8% agar, supplemented with 2.8 mM G418 disulfate purchased from Amresco, Solon, OH, USA) and incubated at 27 °C for 7 days.

Visible colonies were selected for streaking onto fresh G418 agar plates to isolate single transformants. Real-time PCR was performed to detect neoR gene expression and confirm true transformants (as opposed to mutants) under the conditions listed in Supplemental Table S1. The transformation efficiency of the DNA constructs was calculated according to the number of confirmed transformants, as follows: Transformation Efficiency = Number of transformants/Weight of DNA constructs (μg) (Yasui et al. 2009).

### Confirming the presence of centromeric plasmid in transformed BL10

#### PCR-based analysis

The presence of intact circular DNA constructs in the transformants was confirmed using a PCR-based method involving the amplification of three partially overlapping sequences spanning the entire circular construct. The reactions were performed using the SapphireAmp® Fast PCR Master Mix (Takara Bio USA Inc., CA, USA) under the conditions detailed in Supplemental Table S1. To detect the pE2 NG1 plasmid, the following primer sets were used:

- pE2 NG1-18S-2218 (F) and pE2 NG1-18S-4376 (R)
- pE2 NG1-18S-5210 (F) and pE2 NG1-18S-6397 (R)
- pE2 NG1-18S-3453 (F) and pE2 NG1-18S-3128 (R)

To detect the pUNG2-CA plasmid, the following primer sets were used:

- UNG2-CA-151 (F) and UNG2-CA-3476 (R)
- UNG2-CA-2513 (F) and UNG2-CA-4435 (R)
- UNG2-CA-3809 (F) and UNG2-CA-731 (R)

To detect the pUNG-PIT24, 29, or 30 plasmids, the following primer sets were used:

- PIT24-151 (F) and UNG2-PIT24-3310 (R)
- UNG2-PIT24-3643 (F) and UNG2-PIT24-3310 (R)
- UNG2-PIT24-2347 (F) and UNG2-PIT24-4269 (R)

An additional PCR reaction using primer sets D-ef1α (F) and D-ef1α (R), targeting the 5′-UTR of the *ef1-α* gene in BL10 genomic DNA, was performed as a positive control confirming that BL10 cells had been effectively lysed during PCR analysis.

#### Rescue assay

The rescue assay involved selecting a single colony from an agar plate for transfer into a 50-mL test tube containing 5 mL of H3-G418 medium, consisting of peptone, yeast extract/, and glucose (1/2/4 g/L) in 28‰ diluted natural seawater supplemented with 125 µM G418 disulfate. The culture was incubated at 27 °C under shaking (150 rpm) for 7 days to achieve sufficient cell biomass (~ 5 × 10⁷ cells). Following incubation, the cells were centrifuged at 4000 × g for 5 min and washed twice using 28 ppt sterile diluted seawater prior to plasmid extraction using the Presto™ Mini Plasmid kit (Thermo Fisher Scientific Inc., Waltham, MA, USA) in accordance with the manufacturer’s instructions.

The purified plasmids were transformed into *E. coli* ECOS-101 (DH5α) competent cells (YB Biotech, Taipei, Taiwan) using the heat shock method in accordance with the protocol outlined by the manufacturer (https://static.igem.org/mediawiki/2018/d/d2/T--NYMU-Taipei--protocol-competent-cell.pdf). Briefly, competent cells were thawed on ice and gently mixed with purified plasmid DNA. The mixture was incubated on ice, followed by a heat shock at 42 °C to facilitate DNA uptake. After a brief recovery on ice, pre-warmed LB medium (Supplemental Table S2) was added, and the cells were incubated at 37 °C under gentle shaking to promote the expression of antibiotic resistance genes. Transformants were selected via plating on LB agar plates supplemented with 2.8 mM G418. The presence of plasmids in the transformed *E. coli* was verified using colony PCR under the conditions specified in Supplemental Table S1.

#### Stability maintenance assay

The stability maintenance assay was adapted from the methods described by Diner et al. (Karas et al. 2015) with slight modifications. Briefly, a single colony (~ 3–6 × 104 cells) grown on a G418 plate was inoculated into a 50-mL test tube containing 5 mL of H3-G418 medium and incubated under shaking (150 rpm) at 27 °C for 1 week. Following incubation, a 200-μL aliquot of the culture was then transferred into a 24-well plate, such that each well contained 2 mL of GYSS medium (Supplemental Table S2) (with or without 125 μM G418) (*n* = 3 for each condition). After incubating the cultures at 27 °C for 7 days, a 200-μL aliquot from each well was transferred into wells containing the same medium and cultured under identical conditions. This transfer-incubation process was repeated for two additional rounds, resulting in a total of four rounds.

At the conclusion of the final round, a 10-μL sample from each well was diluted in 990 μL of sterile seawater, after which 3 μL of the diluted mixture was plated onto an M3 agar plate. Prior to incubation, the agar plates were examined under a light microscope. Cell aggregates that had not been properly dispersed were marked to avoid selecting colonies that originated from aggregates rather than single cells. After incubating the plate at 27 °C for 3 days, 50 single colonies were selected at random for inoculation onto a G418 agar plate. After incubation at 27 °C for 10 days, the number of colonies (m) capable of growing on the G418 agar plate was recorded.

The maintenance rate (%), number of cell divisions, and segregation efficiency were calculated as follows:

- **Maintenance rate** (%), *y* = *m*/50, where *m* indicates the number of colonies capable of growing on the G418 agar plate.
- **Number of cell divisions**, *n* = log *r*/log 2, where *r* indicates the fold increase in cell count during the four successive rounds of culturing.
- **Segregation efficiency**, *x* = *y*^*1*/*n*^.

## Results

### Initial transformation strategy using linear and circular constructs

**Fig. 1:**
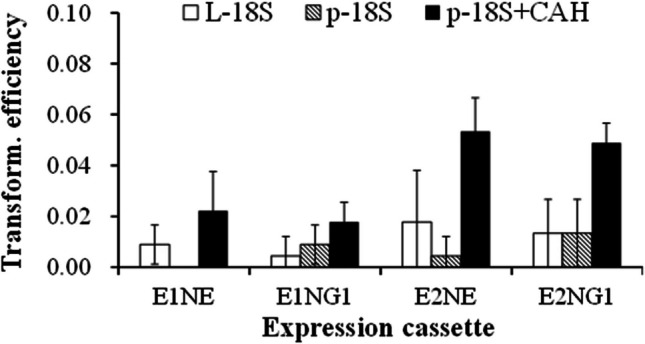
Transformation efficiency of three vector types. L-18S refers to a linear vector with partial 18S rDNA sequences flanking the expression cassette. p-18S refers to a circular vector with partial 18S rDNA sequences flanking the expression cassette. p-18S-CA refers to the aforementioned circular vector with an inserted CA sequence

The experiments assessing the initial design and transformation utilized linear DNA constructs flanked by partial 18S rDNA sequences on both sides of the expression cassettes, including L-E1 NE-18S, L-E2 NE-18S, L-E1 NG1-18S, and L-E2 NG1-18S. Our decision to include partial 18S rDNA sequences was based on the high number of 18S rDNA gene copies in *A. limacinum* (Yasui et al. 2009), which should facilitate the targeted integration of expression cassettes into 18S rDNA loci, while minimizing the risk of insertional mutagenesis (Andreou et al. 2021; White and Garcin 2016). The *neoR* gene—previously used in transformation vectors for thraustochytrid species—was selected as the antibiotic resistance marker. To ensure stable and sufficient expression of *neoR*, this process was performed using promoters and terminators derived from endogenous BL10 housekeeping genes, including *ef1-α*, *gapdh*, and *ubiquitin*. Despite this design strategy, the transformation efficiencies of these linear constructs were suboptimal, with none of the constructs achieving transformation efficiency of greater than 0.02 (Fig. 1).

To improve the transformation efficiency, we modified the circular plasmids used as PCR templates for generating the linear constructs by including the plasmid maintenance sequence CEN6-ARSH4 (CA). The original sequence derived from an artificial yeast chromosome combined two functional elements, including CEN6 (functioning as a centromere) and ARSH4 (functioning at the origin of replication) (Dahmen et al. 2023). The modified circular plasmids were then directly transferred into BL10.

The transformation efficiency of the circular constructs containing the CA sequence was significantly higher than that of the circular plasmids lacking the CA sequence and linear DNA constructs (Fig. 1).

**Fig. 2:**
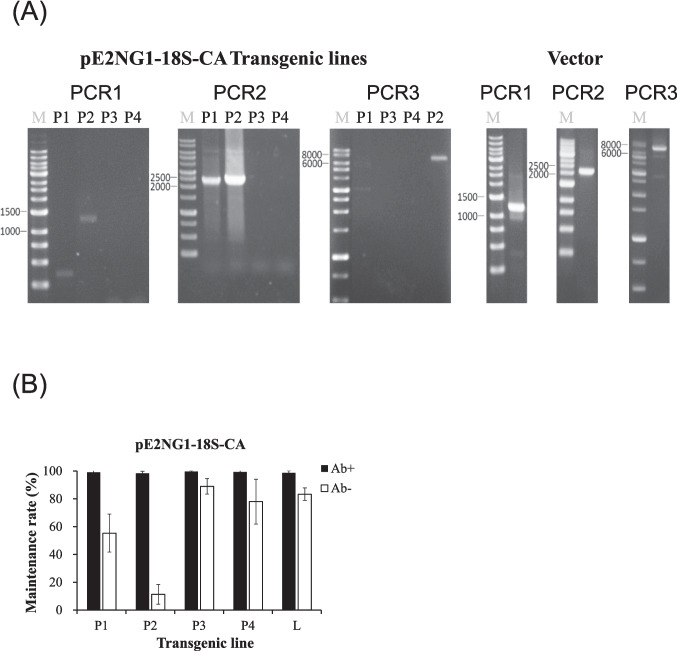
**A** Presence of centromeric plasmids in transformed BL10, as confirmed by three PCR reactions (PCR1, 2, and 3) amplifying overlapping sequences on the pE2 NG1-18S-CA vector. **B** Maintenance of antibiotic resistance in five BL10 transgenic lines with pE2 NG1-18S after four culture rounds of culturing in GYSS medium with (Ab +) or without (Ab −) G418. M: DNA marker, P1 ~ P4: BL10 transgenic lines transformed by pE2 NG1-18S-CA vector; L: BL10 transgenic line transformed by L-E2 NG1-18S-CA vector

Four transgenic lines transformed using pE2 NG1-18S-CA were randomly selected for PCR analysis to determine whether the improved transformation efficiency was linked to the role of the CA fragment in maintaining intact circular vectors in BL10 cells. Specifically, the goal was to determine whether all three partially overlapping sequences spanning the entire pE2 NG1-18S-CA vector were present in these transformants. The intact pE2 NG1-18S-CA vector was detected in one of the four transformants (strain P2) (Fig. 2A).

When cultured in antibiotic-free medium, the loss of antibiotic resistance was more rapid in strain P2 compared to the other three transformants derived from pE2 NG1-18S-CA or L-E2 NG1-18S (Fig. 2B). The segregation efficiency of strain P2 in pE2 NG1-18S-CA was estimated at 90.8%, based on the fact that only 10% of the cells retained antibiotic resistance after four generations (approximately 23 cell divisions), resulting in a 5.9 × 10⁶-fold increase in the number of cells during the culture period. These findings indicate that pE2 NG1-18S-CA replicated in P2 cells and was evenly distributed between daughter cells during cell division.

### Enhancing transformation efficiency through vector minimization

Various strategies were also implemented to reduce the size of the pE2 NG1-18S-CA vector in an effort to further enhance transformation efficiency. These modifications included removal of 18S rDNA partial sequences, replacing the longer E2 promoter (1479 bp) with a shorter ubiquitin (U) promoter (745 bp), and substituting the longer G1 terminator (978 bp) with the shorter G2 terminator (330 bp). These changes resulted in the creation of the pUNG2-CA vector.

Transformation tests demonstrated that reducing the vector size significantly improved transformation efficiency from 0.05 ± 0.01 for pE2 NG1-18S-CA to 0.46 ± 0.20 for pUNG2-CA. However, this strategy did not enhance the function of the circular vector as a replicating plasmid in BL10 transformants. PCR analysis revealed that none of the four BL10 strains transformed using the pUNG2-CA vector contained an intact version of the vector (data not shown).

### Identification and evaluation of centromeric-like elements from P. tricornutum

To determine whether the low plasmid maintenance rate was due to incompatibility between the CA sequence and BL10, we replaced the CA sequence in pUNG2-CA with a centromeric-like sequence derived from *Phaeodactylum tricornutum*. This species was selected due to its status as a model organism and the fact that it shares a closer phylogenetic relationship with *A. limacinum* than does to yeast (Ali et al. 2002; Marchan et al. 2018). Moreover, the availability of complete chromosomal sequences in genomic databases (NCBI dataset GCA_000150955.2) enabled the prediction of centromeric-like sequences by identifying regions exhibiting a sudden drop in GC content.

Supplemental Fig. S3 illustrates the position of the predicted centromere on each chromosome of *P. tricornutum*. We selected centromeric-like sequences on chromosomes 24, 29, and 30 (exhibiting relatively low GC content) to undergo synthesis as a replacement for the CA sequence in pUNG2-CA. This resulted in the creation of three new circular vectors: pUNG2-PIT24, pUNG2-PIT29, and pUNG2-PIT30.

**Fig. 3:**
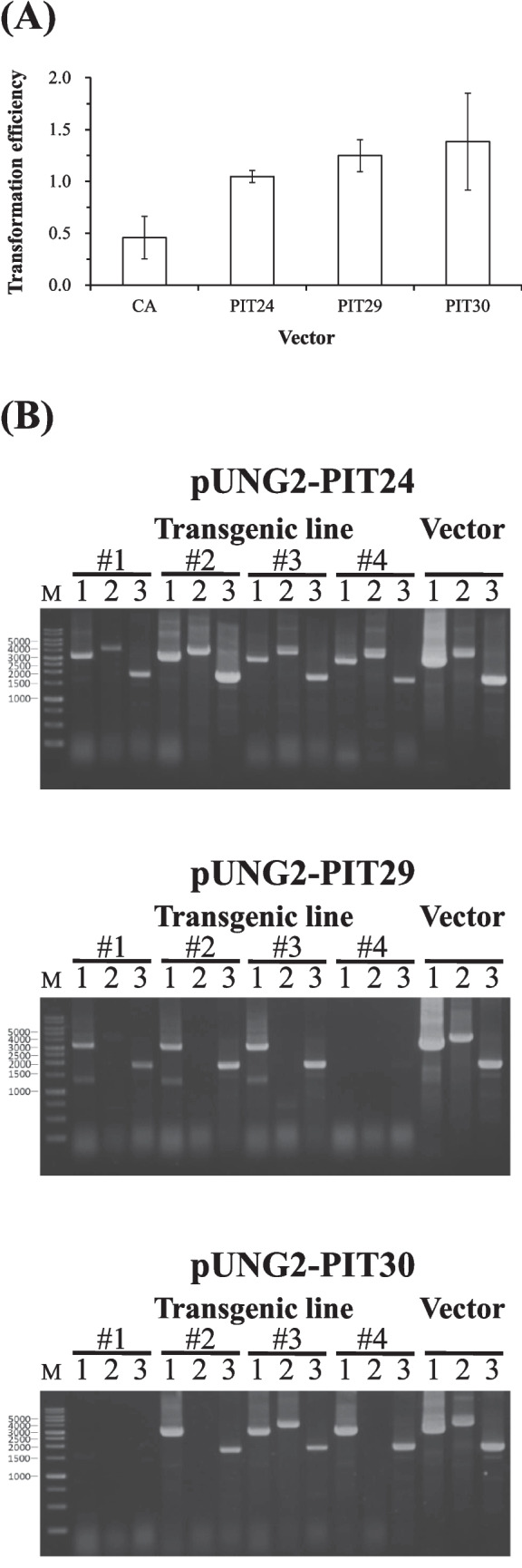
**A** Transformation efficiencies of four pUNG2 vectors with various maintenance sequences, including pUNG2-CA (CA), pUNG2-PIT24 (PIT24), pUNG2-PIT29 (PIT29), and pUNG2-PIT30 (PIT30). **B** Presence of centromeric plasmids in BL10 transformed by pUNG2-PIT24, pUNG2-PIT29, or pUNG2-PIT30, as confirmed by three PCR (PCR1, 2, and 3) amplifying overlapping sequences on these pUNG2 vectors

The transformation efficiency of pUNG2-PIT24 was not significantly higher than that of the original pUNG2-CA or the other two new vectors (Fig. 3A). Nevertheless, pUNG2-PIT24 demonstrated superior performance as an extrachromosomal vector in BL10 cells.

PCR analysis was performed on four transformants selected at random for each vector. Our results revealed that all transformants derived from pUNG2-PIT24 contained the intact vector, whereas pUNG2-PIT30 was detected in only one transformant and the pUNG2-PIT29 vector was not detected in any (Fig. 3B).

Subsequent rescue assays confirmed the presence of the intact pUNG2-PIT24 vector in transformed BL10, as evidenced by the successful extraction of the vector from BL10 cells and its re-transformation into *E. coli* (Supplemental Fig. S4).

The segregation efficiency of pUNG2-PIT24 was calculated at 96.8 ± 0.3% (*n* = 3) from three transformants, indicating that pUNG2-PIT24 replicated within BL10 cells and was evenly distributed between daughter cells after replication—a characteristic consistent with centromeric plasmids.

## Discussion

One previous study utilized a plasmid, pSH65, which contains a bleomycin resistance (BleR) gene and a CA plasmid maintenance sequence, as a vector to introduce the Cre recombinase gene into *A. limacinum* in order to establish a Cre/loxP site-specific recombination system for gene transformation. Note that the CA maintenance sequence used in pSH65 was identical to that used in the episomal vector pPtPUC3 of *Phaeodactylum tricornutum*. The expression of the *Cre* recombinase transgene persisted when the transformed *A. limacinum* was cultured in an antibiotic-containing medium. This enabled the removal of the chloramphenicol resistance (CmR) selectable marker gene (flanked by two loxP sites), which had previously been inserted into the 18S rDNA gene of the host cell. The plasmid pSH65 was gradually lost during subsequent culturing of the transformed *A. limacinum* in an antibiotic-free medium, thereby addressing common GMO-related concerns (Sun et al. 2015).

Plasmid pSH65 is likely the first replicating plasmid successfully applied to *A. limacinum*; however, it remains unclear whether the plasmid maintenance sequence CA on plasmid pSH65 allows an even distribution of the plasmid between daughter cells after replication, which is a characteristic of centromeric plasmids.

Our findings confirmed that the CA sequence from yeast acts as an autonomously replicating sequence in *A. limacinum* and exhibits centromeric sequence functionality. This allows a circular vector to undergo autonomous replication and achieve a roughly even distribution between daughter cells (acting like a centromeric plasmid). Nonetheless, the ability of the CA fragment to preserve a circular vector in *A. limacinum* was suboptimal. This conclusion is supported by the fact that the intact circular vector was detected in only one of four transformants derived from pE2 NG1-18S-CA and none of the four transformants from pUNG2-CA2.

This limitation was largely mitigated by replacing the CA sequence with PIT24, a centromeric-like sequence predicted from the low-GC-content region of chromosome 24 in *P. tricornutum*. All four transformants derived from pUNG2-PIT24 contained the complete pUNG2-PIT24 vector. This suggests that PIT24, likely encompassing the centromeric sequence and replication origin (or autonomously replicating sequence) from chromosome 24 of *P. tricornutum*, is more effective than the CA sequence from yeast at preserving circular vectors in the *A. limacinum* host.

Compared to *P. tricornutum*, we also determined that *A. limacinum* imposes far stricter requirements for plasmid maintenance sequences. Many low-GC-content sequences (GC content ranging from 15 to 35%) from *P. tricornutum* itself—including PIT24, PIT29, and PIT30—or other microorganisms such as *Saccharomyces cerevisiae (CA)*, *Mycoplasma mycoides*, *Cylindrotheca fusiformis*, and *Alteromonas macleodii* are known to possess plasmid maintenance capability or provide effective maintenance of extrachromosomal DNA in *P. tricornutum* (Diner et al. 2017). This study tested various low-GC-content sequences, including three from *P. tricornutum* (PIT24, PIT29, and PIT30), one from *S. cerevisiae* (CA), which have been shown to provide effective maintenance capability of extrachromosomal DNA in *P. tricornutum* (Diner et al. 2017). Among these, only PIT24 from *P. tricornutum* demonstrated a relatively strong ability to maintain circular vectors as extrachromosomal plasmids.

In conclusion, this study presents significant advancements in the genetic engineering of *Aurantiochytrium limacinum*, particularly through the design, construction, and evaluation of plasmids endowed with centromeric-like properties. The incorporation of *Phaeodactylum tricornutum*-derived autonomously replicating sequences (ARS) was shown to enhance plasmid stability and enable sustained transient gene expression within the *A. limacinum* BL10 strain. The ability of these ARS elements was shown to facilitate the episomal maintenance of plasmids without genomic integration represents a milestone in the development of reliable, non-integrative gene delivery systems for this non-model organism. Our findings underscore the feasibility of using heterologous ARS elements in thraustochytrids and lay the groundwork for more sophisticated genetic manipulation strategies, including iterative metabolic engineering, synthetic biology applications, and functional genomic studies. The proposed platform provides a valuable foundation for advancing basic as well as applied research in marine protists and related biotechnological applications.

## Supplementary Information

Below is the link to the electronic supplementary material.

Supplementary file1 (PDF 840 KB)

## Data Availability

Data will be made available on request.
