# Supplementary material for: Novel centromeric plasmid for stable extrachromosomal gene expression in Aurantiochytrium limacinum
Source: Appl Microbiol Biotechnol. 2025 Jul 4;109(1):160. doi: 10.1007/s00253-025-13527-w (PMC12227449; doi:10.1007/s00253-025-13527-w)
Supplement: Supplementary file 1 — Supplementary file1 (PDF 840 KB) [file 253_2025_13527_MOESM1_ESM.pdf]

SUPPLEMENTARY MATERIALS

Journal name: Applied Microbiology and Biotechnology

Manuscript Title: Novel centromeric plasmid for stable extrachromosomal gene expression in *Aurantiochytrium limacinum*

The names of the authors:

Person Pesona Renta [a], Cian-Huei Syu [a], Ta-Yu Huang [a], Yi-Ting Chang [a], Yu-Feng Liang [a], Ssu-Ting Chen [a], Po-Wei Weng [a], Ming-Chen Hsu [a], Keng-Hung Lin [a], Tsunglin Liu [a], Anna C.-C. Jang [a], Che-Chia Tsao [d], Han-Jia Lin [b, c], Hung-Yun Lin [b, c], Yi-Min Chen [a,*]

The affiliations and addresses of the authors:

[a] Department of Biotechnology and Bioindustry Sciences, National Cheng Kung University, Tainan 701401, Taiwan

[b] Department of Bioscience and Biotechnology, National Taiwan Ocean University, Keelung 202301, Taiwan

[c] Center of Excellence for the Oceans, National Taiwan Ocean University, Keelung 202301, Taiwan

[d] Department of Biological Sciences and Technology, National University of Tainan, Tainan 700301, Taiwan

[*] Corresponding author

The e-mail address, telephone and fax numbers of the corresponding author:

E-mail address: cohen@mail.ncku.edu.tw (Yi-Min Chen)

Telephone: +886-6-2757575 Ext. 58217

Fax: +886-6-2766490

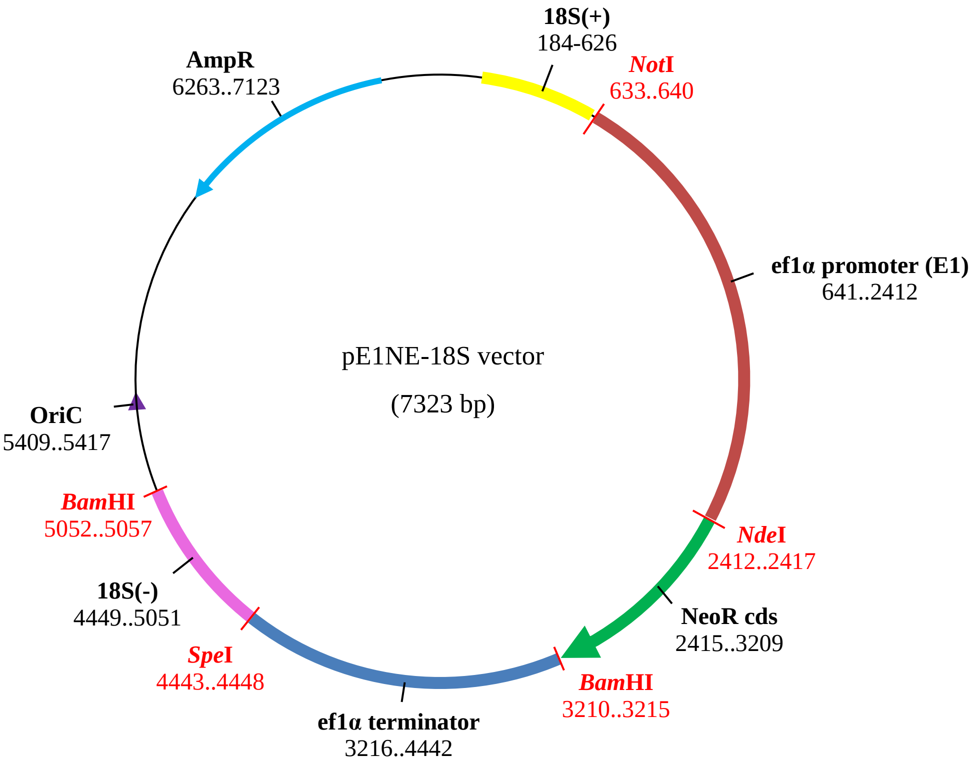

pE1NE-18S vector; 7323 bp

TCGCGCGTTTCGGTGATGACGGTGAAAACCTCTGACACATGCAGCTCCCG
GAGACGGTCACAGCTTGTCTGTAAGCGGATGCCGGGAGCAGACAAGCCCG
TCAGGGCGCGTCAGCGGGTGTTGGCGGGTGTCGGGGCTGGCTTAACTATG
CGGCATCAGAGCAGATTGTACTGAGAGTGCACCATATCGATACCTGCAGT
AATTCTGGAAATAATACATGCTGTAAGAGCCCTGTATGGGGCTGCACTTA
TTAGATTGAAGCCGATTTTATTGGTGAATCATGATAATTGAGCAGATTGA
CATATTTAGTCGATGAATCGTTTGAGTTTCTGCCCCATCAGTTGTCGACG
GTAGTGTATTGGACTACGGTGACTATAACGGGTGACGGAGAGTTAGGGCT
CGACTCCGGAGAGGGAGCCTGAGAGACGGCTACCATATCCAAGGATAGCA
GCAGGCGCGTAAATTACCCACTGTGGACTCCACGAGGTAGTGACGAGAAA
TATCGATGCGAAGCGTGTATGCGTTTTGCTATCGGAATGAGAGCAATGTA
AAACCCTCATCGAGGATCAACTGGAGGGCAAGTCTGGTGCCAGCAGCCGC
GGTAATTCCAGCTCCAGAAGCATATGGGTACCGCGGCCGCTCATGCTCCT
TTCCCGCCAAAAAGAAAGAAGAGGAAAGCACCCCGAAGAAAAGAAAGAAA
TCACCCAAACACCCTCCTCCTTCCTCGTCCACAGACAGCTCAGAATAATA

AAAGCTATCTTTCCATCGCTCTTGACCTAACTCTCTTTCTGCTCCTGTAA
ATTCATCCAACAAATGTTTAGTCTAAGAAACCCATCTGCCTCATACTCCT
ACTTACTACCTTCCTTACTTGAAAGCAGGCAGGCTCACGGCCAGCTTGGC
AGATAGGATAGTTCTCATATCTATTGCTGATCGTTCCCGTTTCTTTCTTA
AAGCAAAGTCTTTTCTTTTCTTTTCTTTTCTTTCCAGGCTCTCCACGTTT
TCAGAAGTAGTACATTTTCTACTTAGTAATTAGAAAGCTTAGTACTTTTT
GCTTTTCTGGATTCTGAAGACTTGGAAATAGAAAGAAATTAAAAATCTTT
TTCTTCTTTCTTTCAGCCTTTGTTGGACTCCCTCGCACGTCTCCTTCTCC
CCCAGCCATCCATCAGCGGGCACTCCACCCGCGCTTCAACGCTCGCTCGA
GTGCGTGCTTATTTGCCTTCAACGCGGCGCGGCGGTTAATATAGTCCCAG
CACTCCTTAAGGGGGGCATCGCAGGGATTATCATTTTAAAAACTGTCACG
GAGTTACATCTTCCCTCGCATCAAAGTGTTCCCGGCCGCGTCGCATATCT
AAGTTTTATAACCTACACCCCTGGTGGGGTAGGGGCGAATTCTATGTACA
CAGCACCTCAGAACTTGCGCGCGTTCCGTGACAAATGAGGGGTGTGGCGG
CGCATTCGGCCGCATCGCCACATTCAGATATCTAACATACCCCCCCTTCG
CGATGGGTGGCAGGCGAGGCGGATTCGCTCGCGAGAGGCGAGGTGCCACA
GCAGACCAGTAACGAGGAGCCAAGGTAGGTGACCACCGACGACTACGACC
ACGACCACAGCCACGGCGGCTGCAGCCACGGGACGCCTCGCATGGCAGCG
CAGCAGCACCAGCAACGACTGCTACAAGGAGTGCAGGGCCGATCTAGACG
CGCCGGAGCCGCACGACCAATGCCGACGCAACGCTGATTCTTCTGGATTC
CCTCTATACATTCATATATATGAAGAGAAGCGGATGAGACGGCCTGCGAA
TAAATGAATGGCTTGGAGTTTGCTTGCTTGCTGTATGCTCGAAGTGCGTG
TGCAGACACAGGCACGACCGAGAGGACAACAGTCTGTGCTTACCTCACCA
GCACATTCTTGCAACGCCATTCGAAGCACGCGAAATCTTGTGGCTCAGAG
CAAAAGGCATCCGTGGTACGGGAACGTGGGGAGCGCTATCAATTTGGAAT
TCAAAATGAGTGAACCAGACAACTAACTGTGACTTGAACTGTTGCTCCAC
GCATCAAAACCAAACCCTTAACAGAAGTAGACCAGTTCGAAGCTACTAGC
ACCAAACAAAATGGGCAAGACGAAGGAGCACGTCAACCTTGTCGTCATCG
GCCACGTCGATGCCGGCAAGTCCACCACCACCGGCCACTTGATCTACAAG
TGCGGTGGTATCGACAAGCGTACCATCGAGAAGTTCGAGAAGGAGGCCGC
CGAGCTCGGTAAGGGTTCCTTCAAGTACGCATGGGTTCTTGACAAGCTCA
AGGCCGAGCGTGAGCGTGGTATCACCATCGATATCGCCTCTGGAAGTTCG
AGTCCCCCAAGTTCGACTTCACCGTCATCGATGCCCCCGGTCACCGTGAT
TTCATCAAGAACATATGATTGAACAAGATGGATTGCACGCAGGTTCTCCG
GCCGCTTGGGTGGAGAGGCTATTCGGCTATGACTGGGCACAACAGACAAT
CGGCTGCTCTGATGCCGCCGTGTTCCGGCTGTCAGCGCAGGGGCGCCCGG
TTCTTTTTGTCAAGACCGACCTGTCCGGTGCCCTGAATGAACTGCAAGAC
GAGGCAGCGCGGCTATCGTGGCTGGCCACGACGGGCGTTCCCTGCGCAGC

TGTGCTCGACGTTGTCACTGAAGCGGGAAGGGACTGGCTGCTATTGGGCG
AAGTGCCGGGGCAGGATCTCCTGTCATCTCACCTTGCTCCTGCCGAGAAA
GTATCCATCATGGCTGATGCAATGCGGCGGCTGCATACGCTTGATCCGGC
TACCTGCCCATTCGACCACCAAGCGAAACATCGCATCGAGCGAGCACGTA
CTCGGATGGAAGCCGGTCTTGTCGATCAGGATGATCTGGACGAAGAGCAT
CAGGGGCTCGCGCCAGCCGAACTGTTCGCCAGGCTCAAGGCGAGCATGCC
CGACGGCGAGGATCTCGTCGTGACCCATGGCGATGCCTGCTTGCCGAATA
TCATGGTGGAAAATGGCCGCTTTTCTGGATTCATCGACTGTGGCCGGCTG
GGTGTGGCGGACCGCTATCAGGACATAGCGTTGGCTACCCGTGATATTGC
TGAAGAGCTTGGCGGCGAATGGGCTGACCGCTTCCTCGTGCTTTACGGTA
TCGCCGCTCCCGATTCGCAGCGCATCGCCTTCTATCGCCTTCTTGACGAG
TTCTTCTAAGGATCCGTGGTTTGACCTCTTATACTTGATCGAAATACTAC
CTACACTTAACCTTTTTTGCGATTTTATCGTGATTACTTTGCGTTTTTCT
TGTTTTATTTCTAATCTTCCTAAGTTGTGTGGCCCCATTGTATTGAACAT
TGCGCAGGCTGATTGTATCTTGTTATCATATTCCTACTTGGATGTCCGTG
AAGTTCGGGGGTTCTGCCATGTTTTCTGGAGGTGTTTTGTTATGAAGCTA
GGATGTGCGTTTCTTGTTTTGTAGTGTTGTCTTGAAATAGTTACTCTTTT
AATTTTTTGTATGTTTGAGAGATTGATTCAGCATGGGTATTCAGGATAGT
GTACGTGATGGTACAATCGTTGTCAATGGTTCGTCGTCTGTTTTTTTAAT
CTAAAGATTTGACATGTCGGAAAAGGTCACAACAGATTTAGTCCCCCGAT
TTTGTAGTGGTTGTTGGAGATTTTGGCATTCTTAGATGATTTTTTTTCTG
TGTTCTGCTACGCTGTTGTAGATACTTCCTTATGTTTAAGATTTATGCTT
GTAAGATACCAGGGTTGATTGAGATAGCTCTAGATGTTTATTTATGGTAT
TGGGTATTGTGAACTACGAAATCATTGATGTTTGAGACTTTTAAAATATA
CTTACGTTTCTACTGTAAAACATGATGGTTATAGATCTCTAAGAAAATAG
GTTTATGGGATGTTAAGCGATGGATAAAAGTTGTTTAAGAGGAAAGTATT
CGATATCGCAACTGTTTCGATCAACGATGGGCAAAGAATCTATTCGCTAA
ATCAAAAACCTATCCTGTCTGTCGTTGGCGTGCGACCAAGAAGCACGGGT
TCGGCAGCAGGTACTGTTTGGAGCTCGAGAAAAGCTTAGTAAACGCTGAG
GTGCCTCCATCGTTGGAGCCATCAGAGAGATTTCTGCTGCTTCACTTTCG
TTGGAAAGTGGAGTGAACCATCTGTTCGATACCCGGACTACAACGTGAGT
TGGGAAACAGTCTTGCTGAGAGGCAGTTTTCGGCCGCTTAGATTCTTTAG
ATTTTGGTAAAGTTCGAAGAGGACATTTGACTGGTTTTGTCTCATAGCTT
GTTTTCTTTACAGAACAACACTTATCATTGATTTAAAGCGGTGCGATAGA
ATTTCAATTGATTCGCTGCATTCTATTTCATAACAGTTAAAATGGGTAGC
GACAATAACCGATCGCGGGTAGAAAACCTGCCAATGCCTGTGACTAGTCT
GGGGATCGAAGATGATTAGATACCATCGTAGTCTAGACCGTAAACGATGC
CGACTTGCGATTGTTGGGTGCTTTATTACATGGGCCTCAGCAGCAGCACA

TGAGAAATCAAAGTCTTTGGGTTCCGGGGGGAGTATGGTCGCAAGGCTGA
AACTTAAAGGAATTGACGGAAGGGCACCACCAGGAGTGGAGCCTGCGGCT
TAATTTGACTCAACACGGGAAAACTTACCAGGTCCAGACATAGGTAGGAT
TGACAGATTGAGAGCTCTTTCATGATTCTATGGGTGGTGGTGCATGGCCG
TTCTTAGTTGGTGGAGTGATTTGTCTGGTTAATTCCGTTAACGAACGAGA
CCTCGGCCTACTAAATAGTGCGTGGTATGGCAACATAGTACGTTTTTAAC
TTCTTAGAGGGACATGTCCGGTTTACGGGCAGGAAGTTCGAGGCAATAAC
AGGTCTGTGATGCCCTTAGATGTTCTGGGCCGCACGCGCGCTACACTGAT
GGGTTCATCGGGTTTTAAATTCAATTTTTGGAATTGAGTGCTTGGTCGGA
AGGCCTGGCTAATCCTTGGAACGCTCATCGTGCTGGGGCTAGATTTTTGC
AGGATCCCCTCTAGAGTCGACCTGCAGGCATGCAAGCTTGGCGTAATCAT
GGTCATAGCTGTTTCCTGTGTGAAATTGTTATCCGCTCACAATTCCACAC
AACATACGAGCCGGAAGCATAAAGTGTAAAGCCTGGGGTGCCTAATGAGT
GAGCTAACTCACATTAATTGCGTTGCGCTCACTGCCCGCTTTCCAGTCGG
GAAACCTGTCGTGCCAGCTGCATTAATGAATCGGCCAACGCGCGGGGAGA
GGCGGTTTGCGTATTGGGCGCTCTTCCGCTTCCTCGCTCACTGACTCGCT
GCGCTCGGTCGTTCGGCTGCGGCGAGCGGTATCAGCTCACTCAAAGGCGG
TAATACGGTTATCCACAGAATCAGGGGATAACGCAGGAAAGAACATGTGA
GCAAAAGGCCAGCAAAAGGCCAGGAACCGTAAAAAGGCCGCGTTGCTGGC
GTTTTTCCATAGGCTCCGCCCCCCTGACGAGCATCACAAAAATCGACGCT
CAAGTCAGAGGTGGCGAAACCCGACAGGACTATAAAGATACCAGGCGTTT
CCCCCTGGAAGCTCCCTCGTGCGCTCTCCTGTTCCGACCCTGCCGCTTAC
CGGATACCTGTCCGCCTTTCTCCCTTCGGGAAGCGTGGCGCTTTCTCATA
GCTCACGCTGTAGGTATCTCAGTTCGGTGTAGGTCGTTCGCTCCAAGCTG
GGCTGTGTGCACGAACCCCCCGTTCAGCCCGACCGCTGCGCCTTATCCGG
TAACTATCGTCTTGAGTCCAACCCGGTAAGACACGACTTATCGCCACTGG
CAGCAGCCACTGGTAACAGGATTAGCAGAGCGAGGTATGTAGGCGGTGCT
ACAGAGTTCTTGAAGTGGTGGCCTAACTACGGCTACACTAGAAGGACAGT
ATTTGGTATCTGCGCTCTGCTGAAGCCAGTTACCTTCGGAAAAAGAGTTG
GTAGCTCTTGATCCGGCAAACAAACCACCGCTGGTAGCGGTGGTTTTTTT
GTTTGCAAGCAGCAGATTACGCGCAGAAAAAAAGGATCTCAAGAAGATCC
TTTGATCTTTTCTACGGGGTCTGACGCTCAGTGGAACGAAAACTCACGTT
AAGGGATTTTGGTCATGAGATTATCAAAAAGGATCTTCACCTAGATCCTT
TTAAATTAAAAATGAAGTTTTAAATCAATCTAAAGTATATATGAGTAAAC
TTGGTCTGACAGTTACCAATGCTTAATCAGTGAGGCACCTATCTCAGCGA
TCTGTCTATTTCGTTCATCCATAGTTGCCTGACTCCCCGTCGTGTAGATA
ACTACGATACGGGAGGGCTTACCATCTGGCCCCAGTGCTGCAATGATACC
GCGAGACCCACGCTCACCGGCTCCAGATTTATCAGCAATAAACCAGCCAG

CCGGAAGGGCCGAGCGCAGAAGTGGTCCTGCAACTTTATCCGCCTCCATC
CAGTCTATTAATTGTTGCCGGGAAGCTAGAGTAAGTAGTTCGCCAGTTAA
TAGTTTGCGCAACGTTGTTGCCATTGCTACAGGCATCGTGGTGTCACGCT
CGTCGTTTGGTATGGCTTCATTCAGCTCCGGTTCCCAACGATCAAGGCGA
GTTACATGATCCCCCATGTTGTGCAAAAAAGCGGTTAGCTCCTTCGGTCC
TCCGATCGTTGTCAGAAGTAAGTTGGCCGCAGTGTTATCACTCATGGTTA
TGGCAGCACTGCATAATTCTCTTACTGTCATGCCATCCGTAAGATGCTTT
TCTGTGACTGGTGAGTACTCAACCAAGTCATTCTGAGAATAGTGTATGCG
GCGACCGAGTTGCTCTTGCCCGGCGTCAATACGGGATAATACCGCGCCAC
ATAGCAGAACTTTAAAAGTGCTCATCATTGGAAAACGTTCTTCGGGGCGA
AAACTCTCAAGGATCTTACCGCTGTTGAGATCCAGTTCGATGTAACCCAC
TCGTGCACCCAACTGATCTTCAGCATCTTTTACTTTCACCAGCGTTTCTG
GGTGAGCAAAAACAGGAAGGCAAAATGCCGCAAAAAAGGGAATAAGGGCG
ACACGGAAATGTTGAATACTCATACTCTTCCTTTTTCAATATTATTGAAG
CATTTATCAGGGTTATTGTCTCATGAGCGGATACATATTTGAATGTATTT
AGAAAAATAAACAAATAGGGGTTCCGCGCACATTTCCCCGAAAAGTGCCA
CCTGACGTCTAAGAAACCATTATTATCATGACATTAACCTATAAAAATAG
GCGTATCACGAGGCCCTTTCGTC

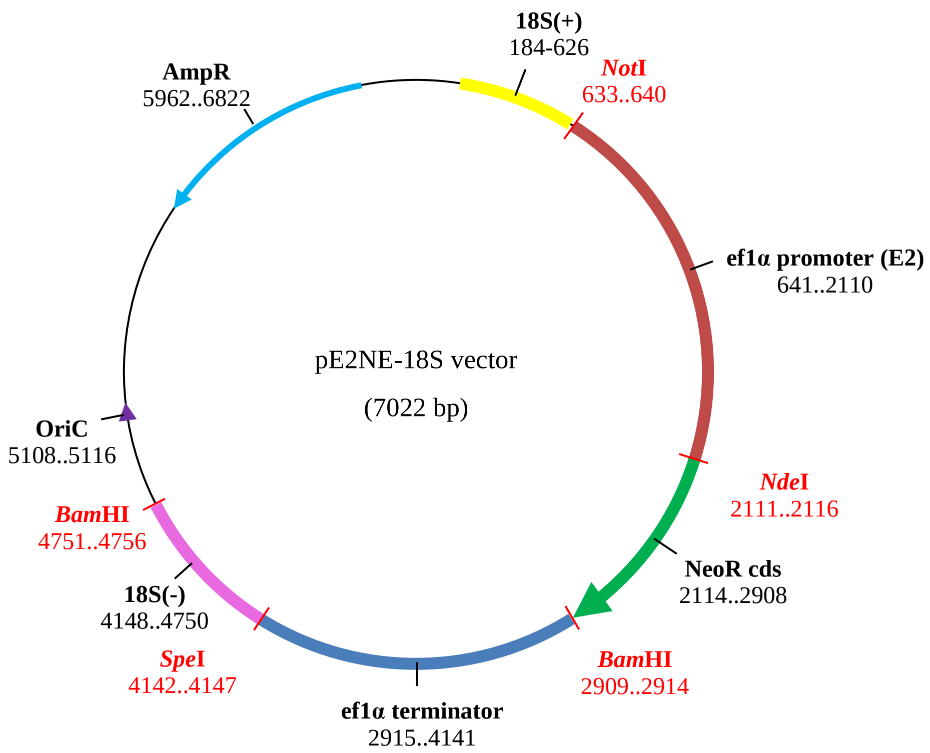

pE2NE-18S vector; 7022 bp

TCGCGCGTTTCGGTGATGACGGTGAAAACCTCTGACACATGCAGCTCCCG
GAGACGGTCACAGCTTGTCTGTAAGCGGATGCCGGGAGCAGACAAGCCCG
TCAGGGCGCGTCAGCGGGTGTTGGCGGGTGTCGGGGCTGGCTTAACTATG
CGGCATCAGAGCAGATTGTACTGAGAGTGCACCATATCGATACCTGCAGT
AATTCTGGAAATAATACATGCTGTAAGAGCCCTGTATGGGGCTGCACTTA
TTAGATTGAAGCCGATTTTATTGGTGAATCATGATAATTGAGCAGATTGA
CATATTTAGTCGATGAATCGTTTGAGTTTCTGCCCCATCAGTTGTCGACG
GTAGTGTATTGGACTACGGTGACTATAACGGGTGACGGAGAGTTAGGGCT
CGACTCCGGAGAGGGAGCCTGAGAGACGGCTACCATATCCAAGGATAGCA
GCAGGCGCGTAAATTACCCACTGTGGACTCCACGAGGTAGTGACGAGAAA
TATCGATGCGAAGCGTGTATGCGTTTTGCTATCGGAATGAGAGCAATGTA
AAACCCTCATCGAGGATCAACTGGAGGGCAAGTCTGGTGCCAGCAGCCGC
GGTAATTCCAGCTCCAGAAGCATATGGGTACCGCGGCCGCTCATGCTCCT
TTCCCGCCAAAAAGAAAGAAGAGGAAAGCACCCCGAAGAAAAGAAAGAAA
TCACCCAAACACCCTCCTCCTTCCTCGTCCACAGACAGCTCAGAATAATA
AAAGCTATCTTTCCATCGCTCTTGACCTAACTCTCTTTCTGCTCCTGTAA
ATTCATCCAACAAATGTTTAGTCTAAGAAACCCATCTGCCTCATACTCCT
ACTTACTACCTTCCTTACTTGAAAGCAGGCAGGCTCACGGCCAGCTTGGC
AGATAGGATAGTTCTCATATCTATTGCTGATCGTTCCCGTTTCTTTCTTA

AAGCAAAGTCTTTTCTTTTCTTTTCTTTTCTTTCCAGGCTCTCCACGTTT
TCAGAAGTAGTACATTTTCTACTTAGTAATTAGAAAGCTTAGTACTTTTT
GCTTTTCTGGATTCTGAAGACTTGGAAATAGAAAGAAATTAAAAATCTTT
TTCTTCTTTCTTTCAGCCTTTGTTGGACTCCCTCGCACGTCTCCTTCTCC
CCCAGCCATCCATCAGCGGGCACTCCACCCGCGCTTCAACGCTCGCTCGA
GTGCGTGCTTATTTGCCTTCAACGCGGCGCGGCGGTTAATATAGTCCCAG
CACTCCTTAAGGGGGGCATCGCAGGGATTATCATTTTAAAAACTGTCACG
GAGTTACATCTTCCCTCGCATCAAAGTGTTCCCGGCCGCGTCGCATATCT
AAGTTTTATAACCTACACCCCTGGTGGGGTAGGGGCGAATTCTATGTACA
CAGCACCTCAGAACTTGCGCGCGTTCCGTGACAAATGAGGGGTGTGGCGG
CGCATTCGGCCGCATCGCCACATTCAGATATCTAACATACCCCCCCTTCG
CGATGGGTGGCAGGCGAGGCGGATTCGCTCGCGAGAGGCGAGGTGCCACA
GCAGACCAGTAACGAGGAGCCAAGGTAGGTGACCACCGACGACTACGACC
ACGACCACAGCCACGGCGGCTGCAGCCACGGGACGCCTCGCATGGCAGCG
CAGCAGCACCAGCAACGACTGCTACAAGGAGTGCAGGGCCGATCTAGACG
CGCCGGAGCCGCACGACCAATGCCGACGCAACGCTGATTCTTCTGGATTC
CCTCTATACATTCATATATATGAAGAGAAGCGGATGAGACGGCCTGCGAA
TAAATGAATGGCTTGGAGTTTGCTTGCTTGCTGTATGCTCGAAGTGCGTG
TGCAGACACAGGCACGACCGAGAGGACAACAGTCTGTGCTTACCTCACCA
GCACATTCTTGCAACGCCATTCGAAGCACGCGAAATCTTGTGGCTCAGAG
CAAAAGGCATCCGTGGTACGGGAACGTGGGGAGCGCTATCAATTTGGAAT
TCAAAATGAGTGAACCAGACAACTAACTGTGACTTGAACTGTTGCTCCAC
GCATCAAAACCAAACCCTTAACAGAAGTAGACCAGTTCGAAGCTACTAGC
ACCAAACAAACATATGATTGAACAAGATGGATTGCACGCAGGTTCTCCGG
CCGCTTGGGTGGAGAGGCTATTCGGCTATGACTGGGCACAACAGACAATC
GGCTGCTCTGATGCCGCCGTGTTCCGGCTGTCAGCGCAGGGGCGCCCGGT
TCTTTTTGTCAAGACCGACCTGTCCGGTGCCCTGAATGAACTGCAAGACG
AGGCAGCGCGGCTATCGTGGCTGGCCACGACGGGCGTTCCCTGCGCAGCT
GTGCTCGACGTTGTCACTGAAGCGGGAAGGGACTGGCTGCTATTGGGCGA
AGTGCCGGGGCAGGATCTCCTGTCATCTCACCTTGCTCCTGCCGAGAAAG
TATCCATCATGGCTGATGCAATGCGGCGGCTGCATACGCTTGATCCGGCT
ACCTGCCCATTCGACCACCAAGCGAAACATCGCATCGAGCGAGCACGTAC
TCGGATGGAAGCCGGTCTTGTCGATCAGGATGATCTGGACGAAGAGCATC
AGGGGCTCGCGCCAGCCGAACTGTTCGCCAGGCTCAAGGCGAGCATGCCC
GACGGCGAGGATCTCGTCGTGACCCATGGCGATGCCTGCTTGCCGAATAT
CATGGTGGAAAATGGCCGCTTTTCTGGATTCATCGACTGTGGCCGGCTGG
GTGTGGCGGACCGCTATCAGGACATAGCGTTGGCTACCCGTGATATTGCT
GAAGAGCTTGGCGGCGAATGGGCTGACCGCTTCCTCGTGCTTTACGGTAT

CGCCGCTCCCGATTCGCAGCGCATCGCCTTCTATCGCCTTCTTGACGAGT
TCTTCTAAGGATCCGTGGTTTGACCTCTTATACTTGATCGAAATACTACC
TACACTTAACCTTTTTTGCGATTTTATCGTGATTACTTTGCGTTTTTCTT
GTTTTATTTCTAATCTTCCTAAGTTGTGTGGCCCCATTGTATTGAACATT
GCGCAGGCTGATTGTATCTTGTTATCATATTCCTACTTGGATGTCCGTGA
AGTTCGGGGGTTCTGCCATGTTTTCTGGAGGTGTTTTGTTATGAAGCTAG
GATGTGCGTTTCTTGTTTTGTAGTGTTGTCTTGAAATAGTTACTCTTTTA
ATTTTTTGTATGTTTGAGAGATTGATTCAGCATGGGTATTCAGGATAGTG
TACGTGATGGTACAATCGTTGTCAATGGTTCGTCGTCTGTTTTTTTAATC
TAAAGATTTGACATGTCGGAAAAGGTCACAACAGATTTAGTCCCCCGATT
TTGTAGTGGTTGTTGGAGATTTTGGCATTCTTAGATGATTTTTTTTCTGT
GTTCTGCTACGCTGTTGTAGATACTTCCTTATGTTTAAGATTTATGCTTG
TAAGATACCAGGGTTGATTGAGATAGCTCTAGATGTTTATTTATGGTATT
GGGTATTGTGAACTACGAAATCATTGATGTTTGAGACTTTTAAAATATAC
TTACGTTTCTACTGTAAAACATGATGGTTATAGATCTCTAAGAAAATAGG
TTTATGGGATGTTAAGCGATGGATAAAAGTTGTTTAAGAGGAAAGTATTC
GATATCGCAACTGTTTCGATCAACGATGGGCAAAGAATCTATTCGCTAAA
TCAAAAACCTATCCTGTCTGTCGTTGGCGTGCGACCAAGAAGCACGGGTT
CGGCAGCAGGTACTGTTTGGAGCTCGAGAAAAGCTTAGTAAACGCTGAGG
TGCCTCCATCGTTGGAGCCATCAGAGAGATTTCTGCTGCTTCACTTTCGT
TGGAAAGTGGAGTGAACCATCTGTTCGATACCCGGACTACAACGTGAGTT
GGGAAACAGTCTTGCTGAGAGGCAGTTTTCGGCCGCTTAGATTCTTTAGA
TTTTGGTAAAGTTCGAAGAGGACATTTGACTGGTTTTGTCTCATAGCTTG
TTTTCTTTACAGAACAACACTTATCATTGATTTAAAGCGGTGCGATAGAA
TTTCAATTGATTCGCTGCATTCTATTTCATAACAGTTAAAATGGTAGCG
ACAATAACCGATCGCGGGTAGAAAACCTGCCAATGCCTGTGACTAGTCTG
GGGATCGAAGATGATTAGATACCATCGTAGTCTAGACCGTAAACGATGCC
GACTTGCGATTGTTGGGTGCTTTATTACATGGGCCTCAGCAGCAGCACAT
GAGAAATCAAAGTCTTTGGGTTCCGGGGGAGTATGGTCGCAAGGCTGAA
ACTTAAAGGAATTGACGGAAGGGCACCACCAGGAGTGGAGCCTGCGGCTT
AATTTGACTCAACACGGGAAAACTTACCAGGTCCAGACATAGGTAGGATT
GACAGATTGAGAGCTCTTTCATGATTCTATGGGTGGTGGTGCATGGCCGT
TCTTAGTTGGTGGAGTGATTTGTCTGGTTAATTCCGTTAACGAACGAGAC
CTCGGCCTACTAAATAGTGCGTGGTATGGCAACATAGTACGTTTTTAACT
TCTTAGAGGGACATGTCCGGTTTACGGGCAGGAAGTTCGAGGCAATAACA
GGTCTGTGATGCCCTTAGATGTTCTGGGCCGCACGCGCGCTACACTGATG
GGTTCATCGGGTTTTAAATTCAATTTTTGGAATTGAGTGCTTGGTCGGAA
GGCCTGGCTAATCCTTGGAACGCTCATCGTGCTGGGGCTAGATTTTTGCA

GGATCCCCTCTAGAGTCGACCTGCAGGCATGCAAGCTTGGCGTAATCATG
GTCATAGCTGTTTCCTGTGTGAAATTGTTATCCGCTCACAATTCCACACA
ACATACGAGCCGGAAGCATAAAGTGTAAAGCCTGGGGTGCCTAATGAGTG
AGCTAACTCACATTAATTGCGTTGCGCTCACTGCCCGCTTTCCAGTCGGG
AAACCTGTCGTGCCAGCTGCATTAATGAATCGGCCAACGCGCGGGGAGAG
GCGGTTTGCGTATTGGGCGCTCTTCCGCTTCCTCGCTCACTGACTCGCTG
CGCTCGGTCGTTCGGCTGCGGCGAGCGGTATCAGCTCACTCAAAGGCGGT
AATACGGTTATCCACAGAATCAGGGGATAACGCAGGAAAGAACATGTGAG
CAAAAGGCCAGCAAAAGGCCAGGAACCGTAAAAAGGCCGCGTTGCTGGCG
TTTTTCCATAGGCTCCGCCCCCCTGACGAGCATCACAAAAATCGACGCTC
AAGTCAGAGGTGGCGAAACCCGACAGGACTATAAAGATACCAGGCGTTTC
CCCCTGGAAGCTCCCTCGTGCGCTCTCCTGTTCCGACCCTGCCGCTTACC
GGATACCTGTCCGCCTTTCTCCCTTCGGGAAGCGTGGCGCTTTCTCATAG
CTCACGCTGTAGGTATCTCAGTTCGGTGTAGGTCGTTCGCTCCAAGCTGG
GCTGTGTGCACGAACCCCCCGTTCAGCCCGACCGCTGCGCCTTATCCGGT
AACTATCGTCTTGAGTCCAACCCGGTAAGACACGACTTATCGCCACTGGC
AGCAGCCACTGGTAACAGGATTAGCAGAGCGAGGTATGTAGGCGGTGCTA
CAGAGTTCTTGAAGTGGTGGCCTAACTACGGCTACACTAGAAGGACAGTA
TTTGGTATCTGCGCTCTGCTGAAGCCAGTTACCTTCGGAAAAAGAGTTGG
TAGCTCTTGATCCGGCAAACAAACCACCGCTGGTAGCGGTGGTTTTTTTG
TTTGCAAGCAGCAGATTACGCGCAGAAAAAAAGGATCTCAAGAAGATCCT
TTGATCTTTTCTACGGGGTCTGACGCTCAGTGGAACGAAAACTCACGTTA
AGGGATTTTGGTCATGAGATTATCAAAAAGGATCTTCACCTAGATCCTTT
TAAATTAAAAATGAAGTTTTAAATCAATCTAAAGTATATATGAGTAAACT
TGGTCTGACAGTTACCAATGCTTAATCAGTGAGGCACCTATCTCAGCGAT
CTGTCTATTTCGTTCATCCATAGTTGCCTGACTCCCCGTCGTGTAGATAA
CTACGATACGGGAGGGCTTACCATCTGGCCCCAGTGCTGCAATGATACCG
CGAGACCCACGCTCACCGGCTCCAGATTTATCAGCAATAAACCAGCCAGC
CGGAAGGGCCGAGCGCAGAAGTGGTCCTGCAACTTTATCCGCCTCCATCC
AGTCTATTAATTGTTGCCGGGAAGCTAGAGTAAGTAGTTCGCCAGTTAAT
AGTTTGCGCAACGTTGTTGCCATTGCTACAGGCATCGTGGTGTCACGCTC
GTCGTTTGGTATGGCTTCATTCAGCTCCGGTTCCCAACGATCAAGGCGAG
TTACATGATCCCCCATGTTGTGCAAAAAAGCGGTTAGCTCCTTCGGTCCT
CCGATCGTTGTCAGAAGTAAGTTGGCCGCAGTGTTATCACTCATGGTTAT
GGCAGCACTGCATAATTCTCTTACTGTCATGCCATCCGTAAGATGCTTTT
CTGTGACTGGTGAGTACTCAACCAAGTCATTCTGAGAATAGTGTATGCGG
CGACCGAGTTGCTCTTGCCCGGCGTCAATACGGGATAATACCGCGCCACA
TAGCAGAACTTTAAAAGTGCTCATCATTGGAAAACGTTCTTCGGGGCGAA

AACTCTCAAGGATCTTACCGCTGTTGAGATCCAGTTCGATGTAACCCACT
CGTGCACCCAACTGATCTTCAGCATCTTTTACTTTCACCAGCGTTTCTGG
GTGAGCAAAAACAGGAAGGCAAAATGCCGCAAAAAAGGGAATAAGGGCGA
CACGGAAATGTTGAATACTCATACTCTTCCTTTTTCAATATTATTGAAGC
ATTTATCAGGGTTATTGTCTCATGAGCGGATACATATTTGAATGTATTTA
GAAAAATAAACAAATAGGGGTTCCGCGCACATTTCCCCGAAAAGTGCCAC
CTGACGTCTAAGAAACCATTATTATCATGACATTAACCTATAAAAATAGG
CGTATCACGAGGCCCTTTCGTC

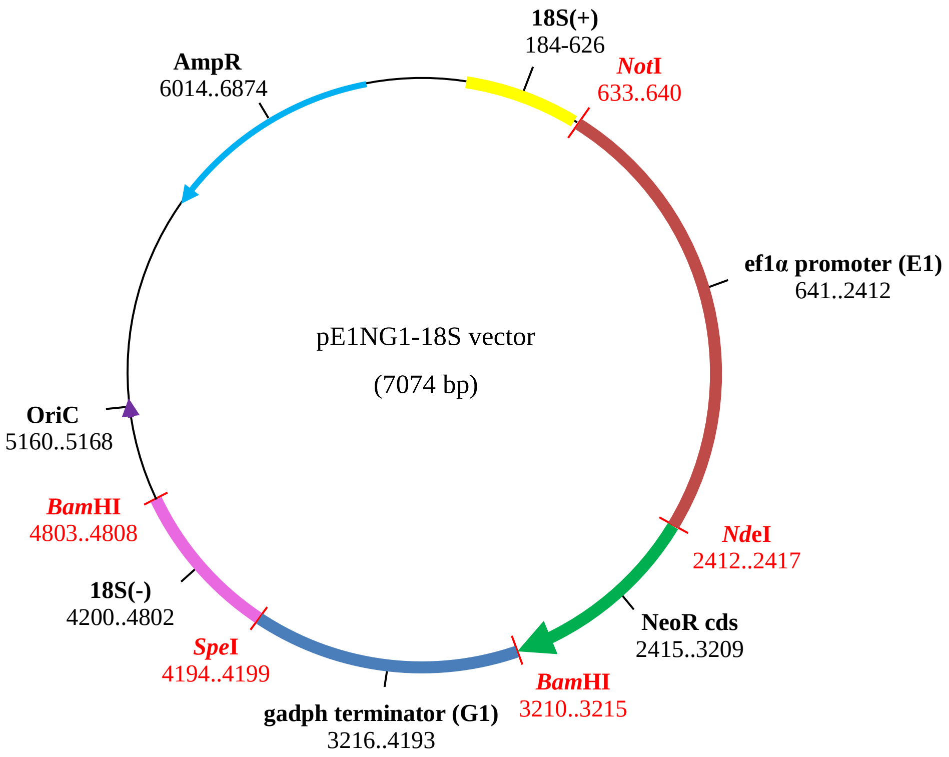

pE1NG1-18S vector; 7074 bp

TCGCGCGTTTCGGTGATGACGGTGAAAACCTCTGACACATGCAGCTCCCG
GAGACGGTCACAGCTTGTCTGTAAGCGGATGCCGGGAGCAGACAAGCCCG
TCAGGGCGCGTCAGCGGGTGTTGGCGGGTGTCGGGGCTGGCTTAACTATG
CGGCATCAGAGCAGATTGTACTGAGAGTGCACCATATCGATACCTGCAGT
AATTCTGGAAATAATACATGCTGTAAGAGCCCTGTATGGGGCTGCACTTA
TTAGATTGAAGCCGATTTTATTGGTGAATCATGATAATTGAGCAGATTGA
CATATTTAGTCGATGAATCGTTTGAGTTTCTGCCCCATCAGTTGTCGACG
GTAGTGTATTGGACTACGGTGACTATAACGGGTGACGGAGAGTTAGGGCT
CGACTCCGGAGAGGGAGCCTGAGAGACGGCTACCATATCCAAGGATAGCA
GCAGGCGCGTAAATTACCCACTGTGGACTCCACGAGGTAGTGACGAGAAA
TATCGATGCGAAGCGTGTATGCGTTTTGCTATCGGAATGAGAGCAATGTA
AAACCCTCATCGAGGATCAACTGGAGGGCAAGTCTGGTGCCAGCAGCCGC
GGTAATTCCAGCTCCAGAAGCATATGGGTACCGCGGCCGCTCATGCTCCT
TTCCCGCCAAAAAGAAAGAAGAGGAAAGCACCCCGAAGAAAAGAAAGAAA
TCACCCAAACACCCTCCTCCTTCCTCGTCCACAGACAGCTCAGAATAATA
AAAGCTATCTTTCCATCGCTCTTGACCTAACTCTCTTTCTGCTCCTGTAA
ATTCATCCAACAAATGTTTAGTCTAAGAAACCCATCTGCCTCATACTCCT
ACTTACTACCTTCCTTACTTGAAAGCAGGCAGGCTCACGGCCAGCTTGGC
AGATAGGATAGTTCTCATATCTATTGCTGATCGTTCCCGTTTCTTTCTTA
AAGCAAAGTCTTTTCTTTTCTTTTCTTTTCTTTCCAGGCTCTCCACGTTT

TCAGAAGTAGTACATTTTCTACTTAGTAATTAGAAAGCTTAGTACTTTTT
GCTTTTCTGGATTCTGAAGACTTGGAAATAGAAAGAAATTAAAAATCTTT
TTCTTCTTTCTTTCAGCCTTTGTTGGACTCCCTCGCACGTCTCCTTCTCC
CCCAGCCATCCATCAGCGGGCACTCCACCCGCGCTTCAACGCTCGCTCGA
GTGCGTGCTTATTTGCCTTCAACGCGGCGCGGCGGTTAATATAGTCCCAG
CACTCCTTAAGGGGGGCATCGCAGGGATTATCATTTTAAAAACTGTCACG
GAGTTACATCTTCCCTCGCATCAAAGTGTTCCCGGCCGCGTCGCATATCT
AAGTTTTATAACCTACACCCCTGGTGGGGTAGGGGCGAATTCTATGTACA
CAGCACCTCAGAACTTGCGCGCGTTCCGTGACAAATGAGGGGTGTGGCGG
CGCATTCGGCCGCATCGCCACATTCAGATATCTAACATACCCCCCCTTCG
CGATGGGTGGCAGGCGAGGCGGATTCGCTCGCGAGAGGCGAGGTGCCACA
GCAGACCAGTAACGAGGAGCCAAGGTAGGTGACCACCGACGACTACGACC
ACGACCACAGCCACGGCGGCTGCAGCCACGGGACGCCTCGCATGGCAGCG
CAGCAGCACCAGCAACGACTGCTACAAGGAGTGCAGGGCCGATCTAGACG
CGCCGGAGCCGCACGACCAATGCCGACGCAACGCTGATTCTTCTGGATTC
CCTCTATACATTCATATATATGAAGAGAAGCGGATGAGACGGCCTGCGAA
TAAATGAATGGCTTGGAGTTTGCTTGCTTGCTGTATGCTCGAAGTGCGTG
TGCAGACACAGGCACGACCGAGAGGACAACAGTCTGTGCTTACCTCACCA
GCACATTCTTGCAACGCCATTCGAAGCACGCGAAATCTTGTGGCTCAGAG
CAAAAGGCATCCGTGGTACGGGAACGTGGGGAGCGCTATCAATTTGGAAT
TCAAAATGAGTGAACCAGACAACTAACTGTGACTTGAACTGTTGCTCCAC
GCATCAAAACCAAACCCTTAACAGAAGTAGACCAGTTCGAAGCTACTAGC
ACCAAACAAAATGGGCAAGACGAAGGAGCACGTCAACCTTGTCGTCATCG
GCCACGTCGATGCCGGCAAGTCCACCACCACCGGCCACTTGATCTACAAG
TGCGGTGGTATCGACAAGCGTACCATCGAGAAGTTCGAGAAGGAGGCCGC
CGAGCTCGGTAAGGGTTCCTTCAAGTACGCATGGGTTCTTGACAAGCTCA
AGGCCGAGCGTGAGCGTGGTATCACCATCGATATCGCCTCTGGAAGTTCG
AGTCCCCCAAGTTCGACTTCACCGTCATCGATGCCCCCGGTCACCGTGAT
TTCATCAAGAACATATGATTGAACAAGATGGATTGCACGCAGGTTCTCCG
GCCGCTTGGGTGGAGAGGCTATTCGGCTATGACTGGGCACAACAGACAAT
CGGCTGCTCTGATGCCGCCGTGTTCCGGCTGTCAGCGCAGGGGCGCCCGG
TTCTTTTTGTCAAGACCGACCTGTCCGGTGCCCTGAATGAACTGCAAGAC
GAGGCAGCGCGGCTATCGTGGCTGGCCACGACGGGCGTTCCCTGCGCAGC
TGTGCTCGACGTTGTCACTGAAGCGGGAAGGGACTGGCTGCTATTGGGCG
AAGTGCCGGGGCAGGATCTCCTGTCATCTCACCTTGCTCCTGCCGAGAAA
GTATCCATCATGGCTGATGCAATGCGGCGGCTGCATACGCTTGATCCGGC
TACCTGCCCATTCGACCACCAAGCGAAACATCGCATCGAGCGAGCACGTA
CTCGGATGGAAGCCGGTCTTGTCGATCAGGATGATCTGGACGAAGAGCAT

CAGGGGCTCGCGCCAGCCGAACTGTTCGCCAGGCTCAAGGCGAGCATGCC
CGACGGCGAGGATCTCGTCGTGACCCATGGCGATGCCTGCTTGCCGAATA
TCATGGTGGAAAATGGCCGCTTTTCTGGATTCATCGACTGTGGCCGGCTG
GGTGTGGCGGACCGCTATCAGGACATAGCGTTGGCTACCCGTGATATTGC
TGAAGAGCTTGGCGGCGAATGGGCTGACCGCTTCCTCGTGCTTTACGGTA
TCGCCGCTCCCGATTCGCAGCGCATCGCCTTCTATCGCCTTCTTGACGAG
TTCTTCTAAGGATCCATGTACCCAATACCACACCGGTAGCTTCTCGCGGC
GGCTGACAAGAAAGATTGTTTTTACACATTTCGAGGCATTAATGACCCTT
ATCGACCTATCGTCTCAGATCATAAAATGCACGAGCATGAAGTACGCGTG
TTGTGACTTGCTTGCGTCCCACCTTCTAGATGGCTTTCTTCTTTTCAAGT
GATTAAAACACCACAGTAGATGAGGACTCTTAGTAAGCACTGAAGAAGCG
AGTAGATAGCCCTCATCCCGTTTCCCTCTTTTCTAACACACTTTGTTTGG
AATTCTAAAATATCTTTATCATCTCTTTTCATTCACAAAACTAGTATTTC
TGCATTAGAAATCATTATCCTATCTGGCACTTTCACTCTGACAAGAACTT
GCGTACATGGCGGGTCCTGGCAATCATTTTACTTGTACAGACCCAAGACT
TTGTGAGTCAGTAAATAGTAAAGAAATGCAGAATAATCATTAGATAATTG
CAAAACCCTGATCTTCAAAATGTTATATCACAAGTACCTACCAAGACATT
TGTATCTTCTTTTTGTCTTATGCATTTTTATTTCCCATAGCGCAATTAAA
AATATTAAATAACTGACGACTCTTTTCTTAAGGATTCGCGATTTGCTAGT
TGCACTATTGAAAAGAGCACAGTATTAATTTCATAGTTTTACTTTGTTGG
CAATCTCAGCAAACATGGGTTTTAATTTTAAAATTAAATAATATTTGTGT
TAGACTCAAAAGATACCTATACAACCCTTAGGGCTCATCGTCAACATTTG
CAAATTAAAACGATTCAGCTCCTTTGGTATGGTCATGTCTGTTTCTGATG
CACTGAAAAATCTTGGACGAACGACACATTTTATTTTCATTAGGGCATCT
AAAAAACGTTGTAAAGAGTATACAATAAGCAAAAAAAAGGAGAGAAAAAA
AAAGGACATATATGGTTCTGCTTTTCTAGTGCTTCAAGTTTGTACTAGTC
TGGGGATCGAAGATGATTAGATACCATCGTAGTCTAGACCGTAAACGATG
CCGACTTGCGATTGTTGGGTGCTTTATTACATGGGCCTCAGCAGCAGCAC
ATGAGAAATCAAAGTCTTTGGGTTCCGGGGGGAGTATGGTCGCAAGGCTG
AAACTTAAAGGAATTGACGGAAGGGCACCACCAGGAGTGGAGCCTGCGGC
TTAATTTGACTCAACACGGGAAAACTTACCAGGTCCAGACATAGGTAGGA
TTGACAGATTGAGAGCTCTTTCATGATTCTATGGGTGGTGGTGCATGGCC
GTTCTTAGTTGGTGGAGTGATTTGTCTGGTTAATTCCGTTAACGAACGAG
ACCTCGGCCTACTAAATAGTGCGTGGTATGGCAACATAGTACGTTTTTAA
CTTCTTAGAGGGACATGTCCGGTTTACGGGCAGGAAGTTCGAGGCAATAA
CAGGTCTGTGATGCCCTTAGATGTTCTGGGCCGCACGCGCGCTACACTGA
TGGGTTCATCGGGTTTTAAATTCAATTTTTGGAATTGAGTGCTTGGTCGG
AAGGCCTGGCTAATCCTTGGAACGCTCATCGTGCTGGGGCTAGATTTTTG

CAGGATCCCCTCTAGAGTCGACCTGCAGGCATGCAAGCTTGGCGTAATCA
TGGTCATAGCTGTTTCCTGTGTGAAATTGTTATCCGCTCACAATTCCACA
CAACATACGAGCCGGAAGCATAAAGTGTAAAGCCTGGGGTGCCTAATGAG
TGAGCTAACTCACATTAATTGCGTTGCGCTCACTGCCCGCTTTCCAGTCG
GGAAACCTGTCGTGCCAGCTGCATTAATGAATCGGCCAACGCGCGGGGAG
AGGCGGTTTGCGTATTGGGCGCTCTTCCGCTTCCTCGCTCACTGACTCGC
TGCGCTCGGTCGTTCGGCTGCGGCGAGCGGTATCAGCTCACTCAAAGGCG
GTAATACGGTTATCCACAGAATCAGGGGATAACGCAGGAAAGAACATGTG
AGCAAAAGGCCAGCAAAAGGCCAGGAACCGTAAAAAGGCCGCGTTGCTGG
CGTTTTTCCATAGGCTCCGCCCCCCTGACGAGCATCACAAAAATCGACGC
TCAAGTCAGAGGTGGCGAAACCCGACAGGACTATAAAGATACCAGGCGTT
TCCCCCTGGAAGCTCCCTCGTGCGCTCTCCTGTTCCGACCCTGCCGCTTA
CCGGATACCTGTCCGCCTTTCTCCCTTCGGGAAGCGTGGCGCTTTCTCAT
AGCTCACGCTGTAGGTATCTCAGTTCGGTGTAGGTCGTTCGCTCCAAGCT
GGGCTGTGTGCACGAACCCCCCGTTCAGCCCGACCGCTGCGCCTTATCCG
GTAACTATCGTCTTGAGTCCAACCCGGTAAGACACGACTTATCGCCACTG
GCAGCAGCCACTGGTAACAGGATTAGCAGAGCGAGGTATGTAGGCGGTGC
TACAGAGTTCTTGAAGTGGTGGCCTAACTACGGCTACACTAGAAGGACAG
TATTTGGTATCTGCGCTCTGCTGAAGCCAGTTACCTTCGGAAAAAGAGTT
GGTAGCTCTTGATCCGGCAAACAAACCACCGCTGGTAGCGGTGGTTTTTT
TGTTTGCAAGCAGCAGATTACGCGCAGAAAAAAAGGATCTCAAGAAGATC
CTTTGATCTTTTCTACGGGGTCTGACGCTCAGTGGAACGAAAACTCACGT
TAAGGGATTTTGGTCATGAGATTATCAAAAAGGATCTTCACCTAGATCCT
TTTAAATTAAAAATGAAGTTTTAAATCAATCTAAAGTATATATGAGTAAA
CTTGGTCTGACAGTTACCAATGCTTAATCAGTGAGGCACCTATCTCAGCG
ATCTGTCTATTTCGTTCATCCATAGTTGCCTGACTCCCCGTCGTGTAGAT
AACTACGATACGGGAGGGCTTACCATCTGGCCCCAGTGCTGCAATGATAC
CGCGAGACCCACGCTCACCGGCTCCAGATTTATCAGCAATAAACCAGCCA
GCCGGAAGGGCCGAGCGCAGAAGTGGTCCTGCAACTTTATCCGCCTCCAT
CCAGTCTATTAATTGTTGCCGGGAAGCTAGAGTAAGTAGTTCGCCAGTTA
ATAGTTTGCGCAACGTTGTTGCCATTGCTACAGGCATCGTGGTGTCACGC
TCGTCGTTTGGTATGGCTTCATTCAGCTCCGGTTCCCAACGATCAAGGCG
AGTTACATGATCCCCCATGTTGTGCAAAAAAGCGGTTAGCTCCTTCGGTC
CTCCGATCGTTGTCAGAAGTAAGTTGGCCGCAGTGTTATCACTCATGGTT
ATGGCAGCACTGCATAATTCTCTTACTGTCATGCCATCCGTAAGATGCTT
TTCTGTGACTGGTGAGTACTCAACCAAGTCATTCTGAGAATAGTGTATGC
GGCGACCGAGTTGCTCTTGCCCGGCGTCAATACGGGATAATACCGCGCCA
CATAGCAGAACTTTAAAAGTGCTCATCATTGGAAAACGTTCTTCGGGGCG

AAAACTCTCAAGGATCTTACCGCTGTTGAGATCCAGTTCGATGTAACCCA
CTCGTGCACCCAACTGATCTTCAGCATCTTTTACTTTCACCAGCGTTTCT
GGGTGAGCAAAAACAGGAAGGCAAAATGCCGCAAAAAAGGGAATAAGGGC
GACACGGAAATGTTGAATACTCATACTCTTCCTTTTTCAATATTATTGAA
GCATTTATCAGGGTTATTGTCTCATGAGCGGATACATATTTGAATGTATT
TAGAAAAATAAACAAATAGGGGTTCCGCGCACATTTCCCCGAAAAGTGCC
ACCTGACGTCTAAGAAACCATTATTATCATGACATTAACCTATAAAAATA
GGCGTATCACGAGGCCCTTTCGTC

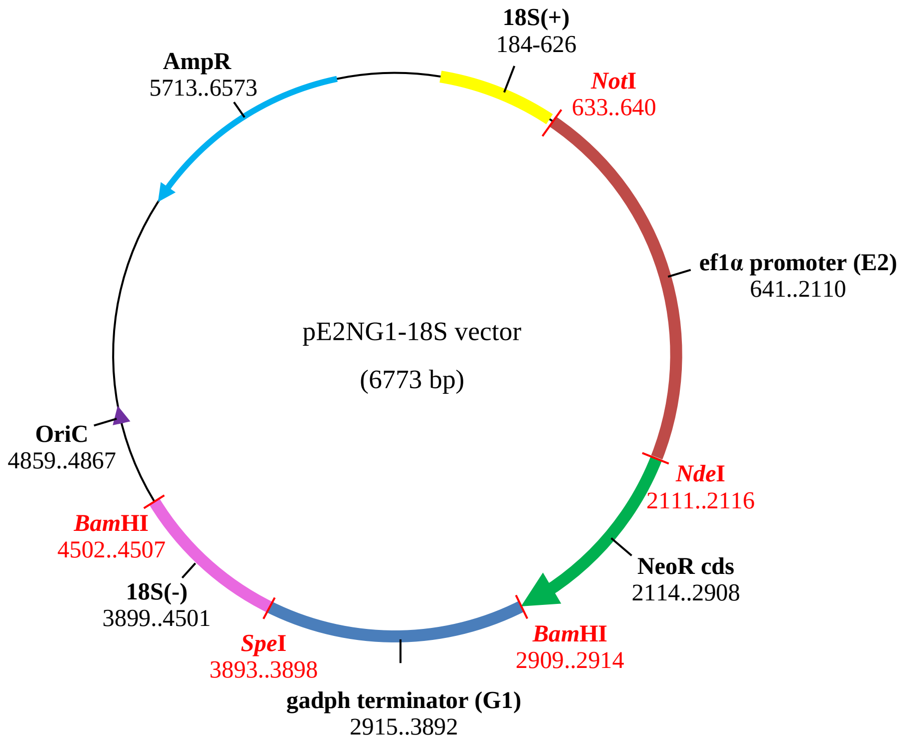

pE2NG1-18S vector; 6773 bp

TCGCGCGTTTCGGTGATGACGGTGAAAACCTCTGACACATGCAGCTCCCG
GAGACGGTCACAGCTTGTCTGTAAGCGGATGCCGGGAGCAGACAAGCCCG
TCAGGGCGCGTCAGCGGGTGTTGGCGGGTGTCGGGGCTGGCTTAACTATG
CGGCATCAGAGCAGATTGTACTGAGAGTGCACCATATCGATACCTGCAGT
AATTCTGGAAATAATACATGCTGTAAGAGCCCTGTATGGGGCTGCACTTA
TTAGATTGAAGCCGATTTTATTGGTGAATCATGATAATTGAGCAGATTGA
CATATTTAGTCGATGAATCGTTTGAGTTTCTGCCCCATCAGTTGTCGACG
GTAGTGTATTGGACTACGGTGACTATAACGGGTGACGGAGAGTTAGGGCT
CGACTCCGGAGAGGGAGCCTGAGAGACGGCTACCATATCCAAGGATAGCA
GCAGGCGCGTAAATTACCCACTGTGGACTCCACGAGGTAGTGACGAGAAA
TATCGATGCGAAGCGTGTATGCGTTTTGCTATCGGAATGAGAGCAATGTA
AAACCCTCATCGAGGATCAACTGGAGGGCAAGTCTGGTGCCAGCAGCCGC
GGTAATTCCAGCTCCAGAAGCATATGGGTACCGCGGCCGCTCATGCTCCT
TTCCCGCCAAAAAGAAAGAAGAGGAAAGCACCCCGAAGAAAAGAAAGAAA
TCACCCAAACACCCTCCTCCTTCCTCGTCCACAGACAGCTCAGAATAATA
AAAGCTATCTTTCCATCGCTCTTGACCTAACTCTCTTTCTGCTCCTGTAA
ATTCATCCAACAAATGTTTAGTCTAAGAAACCCATCTGCCTCATACTCCT
ACTTACTACCTTCCTTACTTGAAAGCAGGCAGGCTCACGGCCAGCTTGGC
AGATAGGATAGTTCTCATATCTATTGCTGATCGTTCCCGTTTCTTTCTTA

AAGCAAAGTCTTTTCTTTTCTTTTCTTTTCTTTCCAGGCTCTCCACGTTT
TCAGAAGTAGTACATTTTCTACTTAGTAATTAGAAAGCTTAGTACTTTTT
GCTTTTCTGGATTCTGAAGACTTGGAAATAGAAAGAAATTAAAAATCTTT
TTCTTCTTTCTTTCAGCCTTTGTTGGACTCCCTCGCACGTCTCCTTCTCC
CCCAGCCATCCATCAGCGGCACTCCACCCGCGCTTCAACGCTCGCTCGA
GTGCGTGCTTATTTGCCTTCAACGCGGCGCGGCGGTTAATATAGTCCCAG
CACTCCTTAAGGGGGGCATCGCAGGGATTATCATTTTAAAAACTGTCACG
GAGTTACATCTTCCCTCGCATCAAAGTGTTCCCGGCCGCGTCGCATATCT
AAGTTTTATAACCTACACCCCTGGTGGGGTAGGGGCGAATTCTATGTACA
CAGCACCTCAGAACTTGCGCGCGTTCCGTGACAAATGAGGGGTGTGGCGG
CGCATTCGGCCGCATCGCCACATTCAGATATCTAACATACCCCCCCTTCG
CGATGGGTGGCAGGCGAGGCGGATTCGCTCGCGAGAGGCGAGGTGCCACA
GCAGACCAGTAACGAGGAGCCAAGGTAGGTGACCACCGACGACTACGACC
ACGACCACAGCCACGGCGGCTGCAGCCACGGGACGCCTCGCATGGCAGCG
CAGCAGCACCAGCAACGACTGCTACAAGGAGTGCAGGGCCGATCTAGACG
CGCCGGAGCCGCACGACCAATGCCGACGCAACGCTGATTCTTCTGGATTC
CCTCTATACATTCATATATATGAAGAGAAGCGGATGAGACGGCCTGCGAA
TAAATGAATGGCTTGGAGTTTGCTTGCTTGCTGTATGCTCGAAGTGCGTG
TGCAGACACAGGCACGACCGAGAGGACAACAGTCTGTGCTTACCTCACCA
GCACATTCTTGCAACGCCATTCGAAGCACGCGAAATCTTGTGGCTCAGAG
CAAAAGGCATCCGTGGTACGGGAACGTGGGGAGCGCTATCAATTTGGAAT
TCAAAATGAGTGAACCAGACAACTAACTGTGACTTGAACTGTTGCTCCAC
GCATCAAAACCAAACCCTTAACAGAAGTAGACCAGTTCGAAGCTACTAGC
ACCAAACAAACATATGATTGAACAAGATGGATTGCACGCAGGTTCTCCGG
CCGCTTGGGTGGAGAGGCTATTCGGCTATGACTGGGCACAACAGACAATC
GGCTGCTCTGATGCCGCCGTGTTCCGGCTGTCAGCGCAGGGGCGCCCGGT
TCTTTTTGTCAAGACCGACCTGTCCGGTGCCCTGAATGAACTGCAAGACG
AGGCAGCGCGGCTATCGTGGCTGGCCACGACGGGCGTTCCCTGCGCAGCT
GTGCTCGACGTTGTCACTGAAGCGGGAAGGGACTGGCTGCTATTGGGCGA
AGTGCCGGGGCAGGATCTCCTGTCATCTCACCTTGCTCCTGCCGAGAAAG
TATCCATCATGGCTGATGCAATGCGGCGGCTGCATACGCTTGATCCGGCT
ACCTGCCCATTCGACCACCAAGCGAAACATCGCATCGAGCGAGCACGTAC
TCGGATGGAAGCCGGTCTTGTCGATCAGGATGATCTGGACGAAGAGCATC
AGGGGCTCGCGCCAGCCGAACTGTTCGCCAGGCTCAAGGCGAGCATGCCC
GACGGCGAGGATCTCGTCGTGACCCATGGCGATGCCTGCTTGCCGAATAT
CATGGTGGAAAATGGCCGCTTTTCTGGATTCATCGACTGTGGCCGGCTGG
GTGTGGCGGACCGCTATCAGGACATAGCGTTGGCTACCCGTGATATTGCT
GAAGAGCTTGGCGGCGAATGGGCTGACCGCTTCCTCGTGCTTTACGGTAT

CGCCGCTCCCGATTCGCAGCGCATCGCCTTCTATCGCCTTCTTGACGAGT
TCTTCTAAGGATCCATGTACCCAATACCACACCGGTAGCTTCTCGCGGCG
GCTGACAAGAAAGATTGTTTTTACACATTTCGAGGCATTAATGACCCTTA
TCGACCTATCGTCTCAGATCATAAAATGCACGAGCATGAAGTACGCGTGT
TGTGACTTGCTTGCGTCCCACCTTCTAGATGGCTTTCTTCTTTTCAAGTG
ATTAAAACACCACAGTAGATGAGGACTCTTAGTAAGCACTGAAGAAGCGA
GTAGATAGCCCTCATCCCGTTTCCCTCTTTTCTAACACACTTTGTTTGGA
ATTCTAAAATATCTTTATCATCTCTTTTCATTCACAAAACTAGTATTTCT
GCATTAGAAATCATTATCCTATCTGGCACTTTCACTCTGACAAGAACTTG
CGTACATGGCGGGTCCTGGCAATCATTTTACTTGTACAGACCCAAGACTT
TGTGAGTCAGTAAATAGTAAAGAAATGCAGAATAATCATTAGATAATTGC
AAAACCCTGATCTTCAAAATGTTATATCACAAGTACCTACCAAGACATTT
GTATCTTCTTTTTGTCTTATGCATTTTTATTTCCCATAGCGCAATTAAAA
ATATTAAATAACTGACGACTCTTTTCTTAAGGATTCGCGATTTGCTAGTT
GCACTATTGAAAAGAGCACAGTATTAATTTCATAGTTTTACTTTGTTGGC
AATCTCAGCAAACATGGGTTTTAATTTTAAAATTAAATAATATTTGTGTT
AGACTCAAAAGATACCTATACAACCCTTAGGGCTCATCGTCAACATTTGC
AAATTAAAACGATTCAGCTCCTTTGGTATGGTCATGTCTGTTTCTGATGC
ACTGAAAAATCTTGGACGAACGACACATTTTATTTTCATTAGGGCATCTA
AAAAACGTTGTAAAGAGTATACAATAAGCAAAAAAAAGGAGAGAAAAAAA
AAGGACATATATGGTTCTGCTTTTCTAGTGCTTCAAGTTTGTACTAGTCT
GGGGATCGAAGATGATTAGATACCATCGTAGTCTAGACCGTAAACGATGC
CGACTTGCGATTGTTGGGTGCTTTATTACATGGGCCTCAGCAGCAGCACA
TGAGAAATCAAAGTCTTTGGGTTCCGGGGGGAGTATGGTCGCAAGGCTGA
AACTTAAAGGAATTGACGGAAGGGCACCACCAGGAGTGGAGCCTGCGGCT
TAATTTGACTCAACACGGGAAAACTTACCAGGTCCAGACATAGGTAGGAT
TGACAGATTGAGAGCTCTTTCATGATTCTATGGGTGGTGGTGCATGGCCG
TTCTTAGTTGGTGGAGTGATTTGTCTGGTTAATTCCGTTAACGAACGAGA
CCTCGGCCTACTAAATAGTGCGTGGTATGGCAACATAGTACGTTTTTAAC
TTCTTAGAGGGACATGTCCGGTTTACGGGCAGGAAGTTCGAGGCAATAAC
AGGTCTGTGATGCCCTTAGATGTTCTGGGCCGCACGCGCGCTACACTGAT
GGGTTCATCGGGTTTTAAATTCAATTTTTGGAATTGAGTGCTTGGTCGGA
AGGCCTGGCTAATCCTTGGAACGCTCATCGTGCTGGGGCTAGATTTTTGC
AGGATCCCCTCTAGAGTCGACCTGCAGGCATGCAAGCTTGGCGTAATCAT
GGTCATAGCTGTTTCCTGTGTGAAATTGTTATCCGCTCACAATTCCACAC
AACATACGAGCCGGAAGCATAAAGTGTAAAGCCTGGGGTGCCTAATGAGT
GAGCTAACTCACATTAATTGCGTTGCGCTCACTGCCCGCTTTCCAGTCGG
GAAACCTGTCGTGCCAGCTGCATTAATGAATCGGCCAACGCGCGGGGAGA

GGCGGTTTGCGTATTGGGCGCTCTTCCGCTTCCTCGCTCACTGACTCGCT
GCGCTCGGTCGTTCGGCTGCGGCGAGCGGTATCAGCTCACTCAAAGGCGG
TAATACGGTTATCCACAGAATCAGGGGATAACGCAGGAAAGAACATGTGA
GCAAAAGGCCAGCAAAAGGCCAGGAACCGTAAAAAGGCCGCGTTGCTGGC
GTTTTTCCATAGGCTCCGCCCCCCTGACGAGCATCACAAAAATCGACGCT
CAAGTCAGAGGTGGCGAAACCCGACAGGACTATAAAGATACCAGGCGTTT
CCCCCTGGAAGCTCCCTCGTGCGCTCTCCTGTTCCGACCCTGCCGCTTAC
CGGATACCTGTCCGCCTTTCTCCCTTCGGGAAGCGTGGCGCTTTCTCATA
GCTCACGCTGTAGGTATCTCAGTTCGGTGTAGGTCGTTCGCTCCAAGCTG
GGCTGTGTGCACGAACCCCCCGTTCAGCCCGACCGCTGCGCCTTATCCGG
TAACTATCGTCTTGAGTCCAACCCGGTAAGACACGACTTATCGCCACTGG
CAGCAGCCACTGGTAACAGGATTAGCAGAGCGAGGTATGTAGGCGGTGCT
ACAGAGTTCTTGAAGTGGTGGCCTAACTACGGCTACACTAGAAGGACAGT
ATTTGGTATCTGCGCTCTGCTGAAGCCAGTTACCTTCGGAAAAAGAGTTG
GTAGCTCTTGATCCGGCAAACAAACCACCGCTGGTAGCGGTGGTTTTTTT
GTTTGCAAGCAGCAGATTACGCGCAGAAAAAAAGGATCTCAAGAAGATCC
TTTGATCTTTTCTACGGGGTCTGACGCTCAGTGGAACGAAAACTCACGTT
AAGGGATTTTGGTCATGAGATTATCAAAAAGGATCTTCACCTAGATCCTT
TTAAATTAAAAATGAAGTTTTAAATCAATCTAAAGTATATATGAGTAAAC
TTGGTCTGACAGTTACCAATGCTTAATCAGTGAGGCACCTATCTCAGCGA
TCTGTCTATTTCGTTCATCCATAGTTGCCTGACTCCCCGTCGTGTAGATA
ACTACGATACGGGAGGGCTTACCATCTGGCCCCAGTGCTGCAATGATACC
GCGAGACCCACGCTCACCGGCTCCAGATTTATCAGCAATAAACCAGCCAG
CCGGAAGGGCCGAGCGCAGAAGTGGTCCTGCAACTTTATCCGCCTCCATC
CAGTCTATTAATTGTTGCCGGGAAGCTAGAGTAAGTAGTTCGCCAGTTAA
TAGTTTGCGCAACGTTGTTGCCATTGCTACAGGCATCGTGGTGTCACGCT
CGTCGTTTGGTATGGCTTCATTCAGCTCCGGTTCCCAACGATCAAGGCGA
GTTACATGATCCCCCATGTTGTGCAAAAAAGCGGTTAGCTCCTTCGGTCC
TCCGATCGTTGTCAGAAGTAAGTTGGCCGCAGTGTTATCACTCATGGTTA
TGGCAGCACTGCATAATTCTCTTACTGTCATGCCATCCGTAAGATGCTTT
TCTGTGACTGGTGAGTACTCAACCAAGTCATTCTGAGAATAGTGTATGCG
GCGACCGAGTTGCTCTTGCCCGGCGTCAATACGGGATAATACCGCGCCAC
ATAGCAGAACTTTAAAAGTGCTCATCATTGGAAAACGTTCTTCGGGGCGA
AAACTCTCAAGGATCTTACCGCTGTTGAGATCCAGTTCGATGTAACCCAC
TCGTGCACCCAACTGATCTTCAGCATCTTTTACTTTCACCAGCGTTTCTG
GGTGAGCAAAAACAGGAAGGCAAAATGCCGCAAAAAAGGGAATAAGGGCG
ACACGGAAATGTTGAATACTCATACTCTTCCTTTTTCAATATTATTGAAG
CATTTATCAGGGTTATTGTCTCATGAGCGGATACATATTTGAATGTATTT

AGAAAAATAAACAAATAGGGGTTCCGCGCACATTTCCCCGAAAAGTGCCA
CCTGACGTCTAAGAAACCATTATTATCATGACATTAACCTATAAAAATAG
GCGTATCACGAGGCCCTTTCGTC

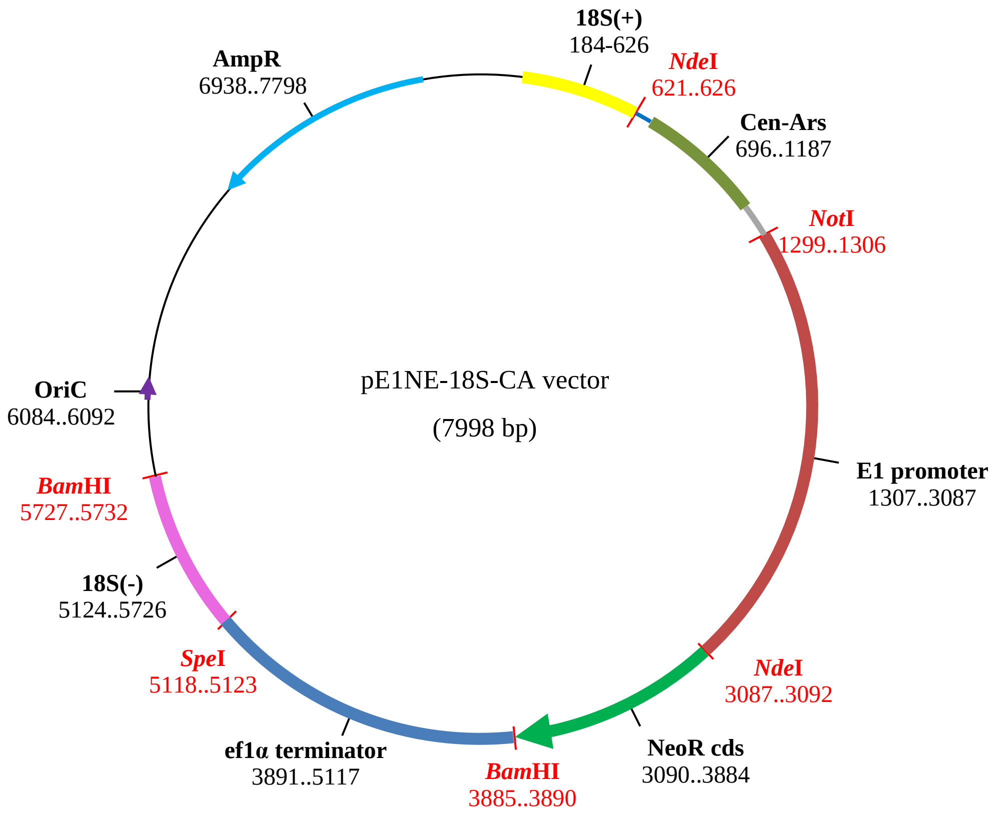

pE1NE-18S-CA; 7998 bp

TCGCGCGTTTCGGTGATGACGGTGAAAACCTCTGACACATGCAGCTCCCG
GAGACGGTCACAGCTTGTCTGTAAGCGGATGCCGGGAGCAGACAAGCCCG
TCAGGGCGCGTCAGCGGGTGTTGGCGGGTGTCGGGGCTGGCTTAACTATG
CGGCATCAGAGCAGATTGTACTGAGAGTGCACCATATCGATACCTGCAGT
AATTCTGGAAATAATACATGCTGTAAGAGCCCTGTATGGGGCTGCACTTA
TTAGATTGAAGCCGATTTTATTGGTGAATCATGATAATTGAGCAGATTGA
CATATTTAGTCGATGAATCGTTTGAGTTTCTGCCCCATCAGTTGTCGACG
GTAGTGTATTGGACTACGGTGACTATAACGGGTGACGGAGAGTTAGGGCT
CGACTCCGGAGAGGGAGCCTGAGAGACGGCTACCATATCCAAGGATAGCA
GCAGGCGCGTAAATTACCCACTGTGGACTCCACGAGGTAGTGACGAGAAA
TATCGATGCGAAGCGTGTATGCGTTTTGCTATCGGAATGAGAGCAATGTA
AAACCCTCATCGAGGATCAACTGGAGGGCAAGTCTGGTGCCAGCAGCCGC
GGTAATTCCAGCTCCAGAAGCATATGGGGCCCGTTGCAGTCACTCCGCTT
TGGTTTCACAGTCAGGAATAACACTAGCTCGTCTTCAGCGAGCATCACGT
GCTATAAAAATAATTATAATTTAAATTTTTTAATATAAATATATAAATTA
AAAATAGAAAGTAAAAAAAGAAATTAAAGAAAAAATAGTTTTTGTTTTCC
GAAGATGTAAAAGACTCTAGGGGGATCGCCAACAAATACTACCTTTTACC
TTGCTCTTCCTGCTCTCAGGTATTAATGCCGAATTGTTTCATCTTGTCTG

TGTAGAAGACCACACACGAAAATCCTGTGATTTTACATTTTACTTATCGT
TAATCGAATGTATATCTATTTAATCTGCTTTTCTTGTCTAATAAATATAT
ATGTAAAGTACGCTTTTTGTTGAAATTTTTTAAACCTTTGTTTATTTTTT
TTTCTTCATTCCGTAACTCTTCTACCTTCTTTATTTACTTTCTAAAATCC
AAATACAAAACATAAAAATAAATAAACACAGAGTAAATTCCCAAATTATT
CCATCATTAAAAGATACGAGGCGCGTGTAAGTTACAGGCAAGCGATCCTA
GTACACTCTATATTTTTTTATGCCTCGGTAATGATTTTCATTTTTTTTTT
CCACCTAGCGGATGACTCTTTTTTTTTCTTAGCGATTGGCATTATCACGC
GGCCGCCTAGCTAGCTCATGCTCCTTTCCCGCCAAAAAGAAAGAAGAGGA
AAGCACCCCGAAGAAAAGAAAGAAATCACCCAAACACCCTCCTCCTTCCT
CGTCCACAGACAGCTCAGAATAATAAAAGCTATCTTTCCATCGCTCTTGA
CCTAACTCTCTTTCTGCTCCTGTAAATTCATCCAACAAATGTTTAGTCTA
AGAAACCCATCTGCCTCATACTCCTACTTACTACCTTCCTTACTTGAAAG
CAGGCAGGCTCACGGCCAGCTTGGCAGATAGGATAGTTCTCATATCTATT
GCTGATCGTTCCCGTTTCTTTCTTAAAGCAAAGTCTTTTCTTTTCTTTTC
TTTTCTTTCCAGGCTCTCCACGTTTTCAGAAGTAGTACATTTTCTACTTA
GTAATTAGAAAGCTTAGTACTTTTTGCTTTTCTGGATTCTGAAGACTTGG
AAATAGAAAGAAATTAAAAATCTTTTTCTTCTTTCTTTCAGCCTTTGTTG
GACTCCCTCGCACGTCTCCTTCTCCCCCAGCCATCCATCAGCGGGCACTC
CACCCGCGCTTCAACGCTCGCTCGAGTGCGTGCTTATTTGCCTTCAACGC
GGCGCGGCGGTTAATATAGTCCCAGCACTCCTTAAGGGGGGCATCGCAGG
GATTATCATTTTAAAAACTGTCACGGAGTTACATCTTCCCTCGCATCAAA
GTGTTCCCGGCCGCGTCGCATATCTAAGTTTTATAACCTACACCCCTGGT
GGGGTAGGGGCGAATTCTATGTACACAGCACCTCAGAACTTGCGCGCGTT
CCGTGACAAATGAGGGGTGTGGCGGCGCATTCGGCCGCATCGCCACATTC
AGATATCTAACATACCCCCCCTTCGCGATGGGTGGCAGGCGAGGCGGATT
CGCTCGCGAGAGGCGAGGTGCCACAGCAGACCAGTAACGAGGAGCCAAGG
TAGGTGACCACCGACGACTACGACCACGACCACAGCCACGGCGGCTGCAG
CCACGGGACGCCTCGCATGGCAGCGCAGCAGCACCAGCAACGACTGCTAC
AAGGAGTGCAGGGCCGATCTAGACGCGCCGGAGCCGCACGACCAATGCCG
ACGCAACGCTGATTCTTCTGGATTCCCTCTATACATTCATATATATGAAG
AGAAGCGGATGAGACGGCCTGCGAATAAATGAATGGCTTGGAGTTTGCTT
GCTTGCTGTATGCTCGAAGTGCGTGTGCAGACACAGGCACGACCGAGAGG
ACAACAGTCTGTGCTTACCTCACCAGCACATTCTTGCAACGCCATTCGAA
GCACGCGAAATCTTGTGGCTCAGAGCAAAAGGCATCCGTGGTACGGAAC
GTGGGGAGCGCTATCAATTTGGAATTCAAAATGAGTGAACCAGACAACTA
ACTGTGACTTGAACTGTTGCTCCACGCATCAAAACCAAACCCTTAACAGA
AGTAGACCAGTTCGAAGCTACTAGCACCAAACAAAATGGGCAAGACGAAG

GAGCACGTCAACCTTGTCGTCATCGGCCACGTCGATGCCGGCAAGTCCAC
CACCACCGGCCACTTGATCTACAAGTGCGGTGGTATCGACAAGCGTACCA
TCGAGAAGTTCGAGAAGGAGGCCGCCGAGCTCGGTAAGGGTTCCTTCAAG
TACGCATGGGTTCTTGACAAGCTCAAGGCCGAGCGTGAGCGTGGTATCAC
CATCGATATCGCCTCTGGAAGTTCGAGTCCCCCAAGTTCGACTTCACCGT
CATCGATGCCCCCGGTCACCGTGATTTCATCAAGAACATATGATTGAACA
AGATGGATTGCACGCAGGTTCTCCGGCCGCTTGGGTGGAGAGGCTATTCG
GCTATGACTGGGCACAACAGACAATCGGCTGCTCTGATGCCGCCGTGTTC
CGGCTGTCAGCGCAGGGGCGCCCGGTTCTTTTTGTCAAGACCGACCTGTC
CGGTGCCCTGAATGAACTGCAAGACGAGGCAGCGCGGCTATCGTGGCTGG
CCACGACGGGCGTTCCCTGCGCAGCTGTGCTCGACGTTGTCACTGAAGCG
GGAAGGGACTGGCTGCTATTGGGCGAAGTGCCGGGGCAGGATCTCCTGTC
ATCTCACCTTGCTCCTGCCGAGAAAGTATCCATCATGGCTGATGCAATGC
GGCGGCTGCATACGCTTGATCCGGCTACCTGCCCATTCGACCACCAAGCG
AAACATCGCATCGAGCGAGCACGTACTCGGATGGAAGCCGGTCTTGTCGA
TCAGGATGATCTGGACGAAGAGCATCAGGGGCTCGCGCCAGCCGAACTGT
TCGCCAGGCTCAAGGCGAGCATGCCCGACGGCGAGGATCTCGTCGTGACC
CATGGCGATGCCTGCTTGCCGAATATCATGGTGGAAAATGGCCGCTTTTC
TGGATTCATCGACTGTGGCCGGCTGGGTGTGGCGGACCGCTATCAGGACA
TAGCGTTGGCTACCCGTGATATTGCTGAAGAGCTTGGCGGCGAATGGGCT
GACCGCTTCCTCGTGCTTTACGGTATCGCCGCTCCCGATTCGCAGCGCAT
CGCCTTCTATCGCCTTCTTGACGAGTTCTTCTAAGGATCCGTGGTTTGAC
CTCTTATACTTGATCGAAATACTACCTACACTTAACCTTTTTTGCGATTT
TATCGTGATTACTTTGCGTTTTTCTTGTTTTATTTCTAATCTTCCTAAGT
TGTGTGGCCCCATTGTATTGAACATTGCGCAGGCTGATTGTATCTTGTTA
TCATATTCCTACTTGGATGTCCGTGAAGTTCGGGGGTTCTGCCATGTTTT
CTGGAGGTGTTTTGTTATGAAGCTAGGATGTGCGTTTCTTGTTTTGTAGT
GTTGTCTTGAAATAGTTACTCTTTTAATTTTTTGTATGTTTGAGAGATTG
ATTCAGCATGGGTATTCAGGATAGTGTACGTGATGGTACAATCGTTGTCA
ATGGTTCGTCGTCTGTTTTTTTAATCTAAAGATTTGACATGTCGGAAAAG
GTCACAACAGATTTAGTCCCCCGATTTTGTAGTGGTTGTTGGAGATTTTG
GCATTCTTAGATGATTTTTTTTCTGTGTTCTGCTACGCTGTTGTAGATAC
TTCCTTATGTTTAAGATTTATGCTTGTAAGATACCAGGGTTGATTGAGAT
AGCTCTAGATGTTTATTTATGGTATTGGGTATTGTGAACTACGAAATCAT
TGATGTTTGAGACTTTTAAAATATACTTACGTTTCTACTGTAAAACATGA
TGGTTATAGATCTCTAAGAAAATAGGTTTATGGGATGTTAAGCGATGGAT
AAAAGTTGTTTAAGAGGAAAGTATTCGATATCGCAACTGTTTCGATCAAC
GATGGGCAAAGAATCTATTCGCTAAATCAAAAACCTATCCTGTCTGTCGT

TGGCGTGCGACCAAGAAGCACGGGTTCGGCAGCAGGTACTGTTTGGAGCT
CGAGAAAAGCTTAGTAAACGCTGAGGTGCCTCCATCGTTGGAGCCATCAG
AGAGATTTCTGCTGCTTCACTTTCGTTGGAAAGTGGAGTGAACCATCTGT
TCGATACCCGGACTACAACGTGAGTTGGGAAACAGTCTTGCTGAGAGGCA
GTTTTCGGCCGCTTAGATTCTTTAGATTTTGGTAAAGTTCGAAGAGGACA
TTTGACTGGTTTTGTCTCATAGCTTGTTTTCTTTACAGAACAACACTTAT
CATTGATTTAAAGCGGTGCGATAGAATTTCAATTGATTCGCTGCATTCTA
TTTCATAACAGTTAAAATGGGTAGCGACAATAACCGATCGCGGGTAGAAA
ACCTGCCAATGCCTGTGACTAGTCTGGGGATCGAAGATGATTAGATACCA
TCGTAGTCTAGACCGTAAACGATGCCGACTTGCGATTGTTGGGTGCTTTA
TTACATGGGCCTCAGCAGCAGCACATGAGAAATCAAAGTCTTTGGGTTCC
GGGGGGAGTATGGTCGCAAGGCTGAAACTTAAAGGAATTGACGGAAGGGC
ACCACCAGGAGTGGAGCCTGCGGCTTAATTTGACTCAACACGGGAAAACT
TACCAGGTCCAGACATAGGTAGGATTGACAGATTGAGAGCTCTTTCATGA
TTCTATGGGTGGTGGTGCATGGCCGTTCTTAGTTGGTGGAGTGATTTGTC
TGGTTAATTCCGTTAACGAACGAGACCTCGGCCTACTAAATAGTGCGTGG
TATGGCAACATAGTACGTTTTTAACTTCTTAGAGGGACATGTCCGGTTTA
CGGGCAGGAAGTTCGAGGCAATAACAGGTCTGTGATGCCCTTAGATGTTC
TGGGCCGCACGCGCGCTACACTGATGGGTTCATCGGGTTTTAAATTCAAT
TTTTGGAATTGAGTGCTTGGTCGGAAGGCCTGGCTAATCCTTGGAACGCT
CATCGTGCTGGGGCTAGATTTTTGCAGGATCCCCTCTAGAGTCGACCTGC
AGGCATGCAAGCTTGGCGTAATCATGGTCATAGCTGTTTCCTGTGTGAAA
TTGTTATCCGCTCACAATTCCACACAACATACGAGCCGGAAGCATAAAGT
GTAAAGCCTGGGGTGCCTAATGAGTGAGCTAACTCACATTAATTGCGTTG
CGCTCACTGCCCGCTTTCCAGTCGGGAAACCTGTCGTGCCAGCTGCATTA
ATGAATCGGCCAACGCGCGGGGAGAGGCGGTTTGCGTATTGGGCGCTCTT
CCGCTTCCTCGCTCACTGACTCGCTGCGCTCGGTCGTTCGGCTGCGGCGA
GCGGTATCAGCTCACTCAAAGGCGGTAATACGGTTATCCACAGAATCAGG
GGATAACGCAGGAAAGAACATGTGAGCAAAAGGCCAGCAAAAGGCCAGGA
ACCGTAAAAAGGCCGCGTTGCTGGCGTTTTTCCATAGGCTCCGCCCCCCT
GACGAGCATCACAAAAATCGACGCTCAAGTCAGAGGTGGCGAAACCCGAC
AGGACTATAAAGATACCAGGCGTTTCCCCCTGGAAGCTCCCTCGTGCGCT
CTCCTGTTCCGACCCTGCCGCTTACCGGATACCTGTCCGCCTTTCTCCCT
TCGGGAAGCGTGGCGCTTTCTCATAGCTCACGCTGTAGGTATCTCAGTTC
GGTGTAGGTCGTTCGCTCCAAGCTGGGCTGTGTGCACGAACCCCCCGTTC
AGCCCGACCGCTGCGCCTTATCCGGTAACTATCGTCTTGAGTCCAACCCG
GTAAGACACGACTTATCGCCACTGGCAGCAGCCACTGGTAACAGGATTAG
CAGAGCGAGGTATGTAGGCGGTGCTACAGAGTTCTTGAAGTGGTGGCCTA

ACTACGGCTACACTAGAAGGACAGTATTTGGTATCTGCGCTCTGCTGAAG
CCAGTTACCTTCGGAAAAAGAGTTGGTAGCTCTTGATCCGGCAAACAAAC
CACCGCTGGTAGCGGTGGTTTTTTTGTTTGCAAGCAGCAGATTACGCGCA
GAAAAAAAGGATCTCAAGAAGATCCTTTGATCTTTTCTACGGGGTCTGAC
GCTCAGTGGAACGAAAACTCACGTTAAGGGATTTTGGTCATGAGATTATC
AAAAAGGATCTTCACCTAGATCCTTTTAAATTAAAAATGAAGTTTTAAAT
CAATCTAAAGTATATATGAGTAAACTTGGTCTGACAGTTACCAATGCTTA
ATCAGTGAGGCACCTATCTCAGCGATCTGTCTATTTCGTTCATCCATAGT
TGCCTGACTCCCCGTCGTGTAGATAACTACGATACGGGAGGGCTTACCAT
CTGGCCCCAGTGCTGCAATGATACCGCGAGACCCACGCTCACCGGCTCCA
GATTTATCAGCAATAAACCAGCCAGCCGGAAGGGCCGAGCGCAGAAGTGG
TCCTGCAACTTTATCCGCCTCCATCCAGTCTATTAATTGTTGCCGGGAAG
CTAGAGTAAGTAGTTCGCCAGTTAATAGTTTGCGCAACGTTGTTGCCATT
GCTACAGGCATCGTGGTGTCACGCTCGTCGTTTGGTATGGCTTCATTCAG
CTCCGGTTCCCAACGATCAAGGCGAGTTACATGATCCCCCATGTTGTGCA
AAAAAGCGGTTAGCTCCTTCGGTCCTCCGATCGTTGTCAGAAGTAAGTTG
GCCGCAGTGTTATCACTCATGGTTATGGCAGCACTGCATAATTCTCTTAC
TGTCATGCCATCCGTAAGATGCTTTTCTGTGACTGGTGAGTACTCAACCA
AGTCATTCTGAGAATAGTGTATGCGGCGACCGAGTTGCTCTTGCCCGGCG
TCAATACGGGATAATACCGCGCCACATAGCAGAACTTTAAAAGTGCTCAT
CATTGGAAAACGTTCTTCGGGGCGAAAACTCTCAAGGATCTTACCGCTGT
TGAGATCCAGTTCGATGTAACCCACTCGTGCACCCAACTGATCTTCAGCA
TCTTTTACTTTCACCAGCGTTTCTGGGTGAGCAAAAACAGGAAGGCAAAA
TGCCGCAAAAAAGGGAATAAGGGCGACACGGAAATGTTGAATACTCATAC
TCTTCCTTTTTCAATATTATTGAAGCATTTATCAGGGTTATTGTCTCATG
AGCGGATACATATTTGAATGTATTTAGAAAAATAAACAAATAGGGGTTCC
GCGCACATTTCCCCGAAAAGTGCCACCTGACGTCTAAGAAACCATTATTA
TCATGACATTAACCTATAAAAATAGGCGTATCACGAGGCCCTTTCGTC

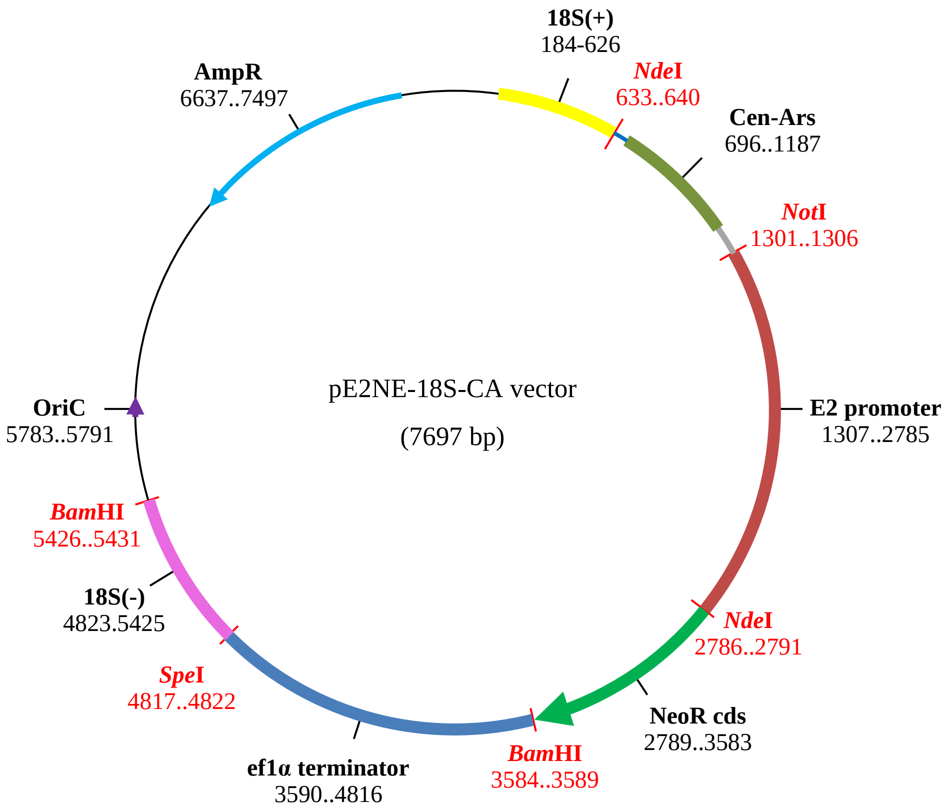

pE2NE-18S-CA; 7697 bp

TCGCGCGTTTCGGTGATGACGGTGAAAACCTCTGACACATGCAGCTCCCG
GAGACGGTCACAGCTTGTCTGTAAGCGGATGCCGGGAGCAGACAAGCCCG
TCAGGGCGCGTCAGCGGGTGTTGGCGGGTGTCGGGGCTGGCTTAACTATG
CGGCATCAGAGCAGATTGTACTGAGAGTGCACCATATCGATACCTGCAGT
AATTCTGGAAATAATACATGCTGTAAGAGCCCTGTATGGGGCTGCACTTA
TTAGATTGAAGCCGATTTTATTGGTGAATCATGATAATTGAGCAGATTGA
CATATTTAGTCGATGAATCGTTTGAGTTTCTGCCCCATCAGTTGTCGACG
GTAGTGTATTGGACTACGGTGACTATAACGGGTGACGGAGAGTTAGGGCT
CGACTCCGGAGAGGGAGCCTGAGAGACGGCTACCATATCCAAGGATAGCA
GCAGGCGCGTAAATTACCCACTGTGGACTCCACGAGGTAGTGACGAGAAA
TATCGATGCGAAGCGTGTATGCGTTTTGCTATCGGAATGAGAGCAATGTA
AAACCCTCATCGAGGATCAACTGGAGGGCAAGTCTGGTGCCAGCAGCCGC
GGTAATTCCAGCTCCAGAAGCATATGGGGCCCGTTGCAGTCACTCCGCTT
TGGTTTCACAGTCAGGAATAACACTAGCTCGTCTTCAGCGAGCATCACGT
GCTATAAAAATAATTATAATTTAAATTTTTTAATATAAATATATAAATTA
AAAATAGAAAGTAAAAAAAGAAATTAAAGAAAAAATAGTTTTTGTTTTCC
GAAGATGTAAAAGACTCTAGGGGGATCGCCAACAAATACTACCTTTTACC

TTGCTCTTCCTGCTCTCAGGTATTAATGCCGAATTGTTTCATCTTGTCTG
TGTAGAAGACCACACACGAAAATCCTGTGATTTTACATTTTACTTATCGT
TAATCGAATGTATATCTATTTAATCTGCTTTTCTTGTCTAATAAATATAT
ATGTAAAGTACGCTTTTTGTTGAAATTTTTTAAACCTTTGTTTATTTTTT
TTTCTTCATTCCGTAACTCTTCTACCTTCTTTATTTACTTTCTAAAATCC
AAATACAAAACATAAAAATAAATAAACACAGAGTAAATTCCCAAATTATT
CCATCATTAAAAGATACGAGGCGCGTGTAAGTTACAGGCAAGCGATCCTA
GTACACTCTATATTTTTTTATGCCTCGGTAATGATTTTCATTTTTTTTTT
CCACCTAGCGGATGACTCTTTTTTTTTCTTAGCGATTGGCATTATCACGC
GGCCGCCTAGCTAGCTCATGCTCCTTTCCCGCCAAAAAGAAAGAAGAGGA
AAGCACCCCGAAGAAAAGAAAGAAATCACCCAAACACCCTCCTCCTTCCT
CGTCCACAGACAGCTCAGAATAATAAAAGCTATCTTTCCATCGCTCTTGA
CCTAACTCTCTTTCTGCTCCTGTAAATTCATCCAACAAATGTTTAGTCTA
AGAAACCCATCTGCCTCATACTCCTACTTACTACCTTCCTTACTTGAAAG
CAGGCAGGCTCACGGCCAGCTTGGCAGATAGGATAGTTCTCATATCTATT
GCTGATCGTTCCCGTTTCTTTCTTAAAGCAAAGTCTTTTCTTTTCTTTTC
TTTTCTTTCCAGGCTCTCCACGTTTTCAGAAGTAGTACATTTTCTACTTA
GTAATTAGAAAGCTTAGTACTTTTTGCTTTTCTGGATTCTGAAGACTTGG
AAATAGAAAGAAATTAAAAATCTTTTTCTTCTTTCTTTCAGCCTTTGTTG
GACTCCCTCGCACGTCTCCTTCTCCCCCAGCCATCCATCAGCGGGCACTC
CACCCGCGCTTCAACGCTCGCTCGAGTGCGTGCTTATTTGCCTTCAACGC
GGCGCGGCGGTTAATATAGTCCCAGCACTCCTTAAGGGGGGCATCGCAGG
GATTATCATTTTAAAAACTGTCACGGAGTTACATCTTCCCTCGCATCAAA
GTGTTCCCGGCCGCGTCGCATATCTAAGTTTTATAACCTACACCCCTGGT
GGGGTAGGGGCGAATTCTATGTACACAGCACCTCAGAACTTGCGCGCGTT
CCGTGACAAATGAGGGGTGTGGCGGCGCATTCGGCCGCATCGCCACATTC
AGATATCTAACATACCCCCCCTTCGCGATGGGTGGCAGGCGAGGCGGATT
CGCTCGCGAGAGGCGAGGTGCCACAGCAGACCAGTAACGAGGAGCCAAGG
TAGGTGACCACCGACGACTACGACCACGACCACAGCCACGGCGGCTGCAG
CCACGGGACGCCTCGCATGGCAGCGCAGCAGCACCAGCAACGACTGCTAC
AAGGAGTGCAGGGCCGATCTAGACGCGCCGGAGCCGCACGACCAATGCCG
ACGCAACGCTGATTCTTCTGGATTCCCTCTATACATTCATATATATGAAG
AGAAGCGGATGAGACGGCCTGCGAATAAATGAATGGCTTGGAGTTTGCTT
GCTTGCTGTATGCTCGAAGTGCGTGTGCAGACACAGGCACGACCGAGAGG
ACAACAGTCTGTGCTTACCTCACCAGCACATTCTTGCAACGCCATTCGAA
GCACGCGAAATCTTGTGGCTCAGAGCAAAAGGCATCCGTGGTACGGGAAC
GTGGGGAGCGCTATCAATTTGGAATTCAAAATGAGTGAACCAGACAACTA
ACTGTGACTTGAACTGTTGCTCCACGCATCAAAACCAAACCCTTAACAGA

AGTAGACCAGTTCGAAGCTACTAGCACCAAACAAACATATGATTGAACAA
GATGGATTGCACGCAGGTTCTCCGGCCGCTTGGGTGGAGAGGCTATTCGG
CTATGACTGGGCACAACAGACAATCGGCTGCTCTGATGCCGCCGTGTTCC
GGCTGTCAGCGCAGGGGCGCCCGGTTCTTTTTGTCAAGACCGACCTGTCC
GGTGCCCTGAATGAACTGCAAGACGAGGCAGCGCGGCTATCGTGGCTGGC
CACGACGGGCGTTCCCTGCGCAGCTGTGCTCGACGTTGTCACTGAAGCGG
GAAGGGACTGGCTGCTATTGGGCGAAGTGCCGGGGCAGGATCTCCTGTCA
TCTCACCTTGCTCCTGCCGAGAAAGTATCCATCATGGCTGATGCAATGCG
GCGGCTGCATACGCTTGATCCGGCTACCTGCCCATTCGACCACCAAGCGA
AACATCGCATCGAGCGAGCACGTACTCGGATGGAAGCCGGTCTTGTCGAT
CAGGATGATCTGGACGAAGAGCATCAGGGGCTCGCGCCAGCCGAACTGTT
CGCCAGGCTCAAGGCGAGCATGCCCGACGGCGAGGATCTCGTCGTGACCC
ATGGCGATGCCTGCTTGCCGAATATCATGGTGGAAAATGGCCGCTTTTCT
GGATTCATCGACTGTGGCCGGCTGGGTGTGGCGGACCGCTATCAGGACAT
AGCGTTGGCTACCCGTGATATTGCTGAAGAGCTTGGCGGCGAATGGGCTG
ACCGCTTCCTCGTGCTTTACGGTATCGCCGCTCCCGATTCGCAGCGCATC
GCCTTCTATCGCCTTCTTGACGAGTTCTTCTAAGGATCCGTGGTTTGACC
TCTTATACTTGATCGAAATACTACCTACACTTAACCTTTTTTGCGATTTT
ATCGTGATTACTTTGCGTTTTTCTTGTTTTATTTCTAATCTTCCTAAGTT
GTGTGGCCCCATTGTATTGAACATTGCGCAGGCTGATTGTATCTTGTTAT
CATATTCCTACTTGGATGTCCGTGAAGTTCGGGGGTTCTGCCATGTTTTC
TGGAGGTGTTTTGTTATGAAGCTAGGATGTGCGTTTCTTGTTTTGTAGTG
TTGTCTTGAAATAGTTACTCTTTTAATTTTTTGTATGTTTGAGAGATTGA
TTCAGCATGGGTATTCAGGATAGTGTACGTGATGGTACAATCGTTGTCAA
TGGTTCGTCGTCTGTTTTTTTAATCTAAAGATTTGACATGTCGGAAAAGG
TCACAACAGATTTAGTCCCCCGATTTTGTAGTGGTTGTTGGAGATTTTGG
CATTCTTAGATGATTTTTTTTCTGTGTTCTGCTACGCTGTTGTAGATACT
TCCTTATGTTTAAGATTTATGCTTGTAAGATACCAGGGTTGATTGAGATA
GCTCTAGATGTTTATTTATGGTATTGGGTATTGTGAACTACGAAATCATT
GATGTTTGAGACTTTTAAAATATACTTACGTTTCTACTGTAAAACATGAT
GGTTATAGATCTCTAAGAAAATAGGTTTATGGGATGTTAAGCGATGGATA
AAAGTTGTTTAAGAGGAAAGTATTCGATATCGCAACTGTTTCGATCAACG
ATGGGCAAAGAATCTATTCGCTAAATCAAAAACCTATCCTGTCTGTCGTT
GGCGTGCGACCAAGAAGCACGGGTTCGGCAGCAGGTACTGTTTGGAGCTC
GAGAAAAGCTTAGTAAACGCTGAGGTGCCTCCATCGTTGGAGCCATCAGA
GAGATTTCTGCTGCTTCACTTTCGTTGGAAAGTGGAGTGAACCATCTGTT
CGATACCCGGACTACAACGTGAGTTGGGAAACAGTCTTGCTGAGAGGCAG
TTTTCGGCCGCTTAGATTCTTTAGATTTTGGTAAAGTTCGAAGAGGACAT

TTGACTGGTTTTGTCTCATAGCTTGTTTTCTTTACAGAACAACACTTATC
ATTGATTTAAAGCGGTGCGATAGAATTTCAATTGATTCGCTGCATTCTAT
TTCATAACAGTTAAAATGGGTAGCGACAATAACCGATCGCGGGTAGAAAA
CCTGCCAATGCCTGTGACTAGTCTGGGGATCGAAGATGATTAGATACCAT
CGTAGTCTAGACCGTAAACGATGCCGACTTGCGATTGTTGGGTGCTTTAT
TACATGGGCCTCAGCAGCAGCACATGAGAAATCAAAGTCTTTGGGTTCCG
GGGGGAGTATGGTCGCAAGGCTGAAACTTAAAGGAATTGACGGAAGGGCA
CCACCAGGAGTGGAGCCTGCGGCTTAATTTGACTCAACACGGGAAAACTT
ACCAGGTCCAGACATAGGTAGGATTGACAGATTGAGAGCTCTTTCATGAT
TCTATGGGTGGTGGTGCATGGCCGTTCTTAGTTGGTGGAGTGATTTGTCT
GGTTAATTCCGTTAACGAACGAGACCTCGGCCTACTAAATAGTGCGTGGT
ATGGCAACATAGTACGTTTTTAACTTCTTAGAGGGACATGTCCGGTTTAC
GGGCAGGAAGTTCGAGGCAATAACAGGTCTGTGATGCCCTTAGATGTTCT
GGGCCGCACGCGCGCTACACTGATGGGTTCATCGGGTTTTAAATTCAATT
TTTGGAATTGAGTGCTTGGTCGGAAGGCCTGGCTAATCCTTGGAACGCTC
ATCGTGCTGGGGCTAGATTTTTGCAGGATCCCCTCTAGAGTCGACCTGCA
GGCATGCAAGCTTGGCGTAATCATGGTCATAGCTGTTTCCTGTGTGAAAT
TGTTATCCGCTCACAATTCCACACAACATACGAGCCGGAAGCATAAAGTG
TAAAGCCTGGGGTGCCTAATGAGTGAGCTAACTCACATTAATTGCGTTGC
GCTCACTGCCCGCTTTCCAGTCGGGAAACCTGTCGTGCCAGCTGCATTAA
TGAATCGGCCAACGCGCGGGGAGAGGCGGTTTGCGTATTGGGCGCTCTTC
CGCTTCCTCGCTCACTGACTCGCTGCGCTCGGTCGTTCGGCTGCGGCGAG
CGGTATCAGCTCACTCAAAGGCGGTAATACGGTTATCCACAGAATCAGGG
GATAACGCAGGAAAGAACATGTGAGCAAAAGGCCAGCAAAAGGCCAGGAA
CCGTAAAAAGGCCGCGTTGCTGGCGTTTTTCCATAGGCTCCGCCCCCCTG
ACGAGCATCACAAAAATCGACGCTCAAGTCAGAGGTGGCGAAACCCGACA
GGACTATAAAGATACCAGGCGTTTCCCCCTGGAAGCTCCCTCGTGCGCTC
TCCTGTTCCGACCCTGCCGCTTACCGGATACCTGTCCGCCTTTCTCCCTT
CGGGAAGCGTGGCGCTTTCTCATAGCTCACGCTGTAGGTATCTCAGTTCG
GTGTAGGTCGTTCGCTCCAAGCTGGGCTGTGTGCACGAACCCCCCGTTCA
GCCCGACCGCTGCGCCTTATCCGGTAACTATCGTCTTGAGTCCAACCCGG
TAAGACACGACTTATCGCCACTGGCAGCAGCCACTGGTAACAGGATTAGC
AGAGCGAGGTATGTAGGCGGTGCTACAGAGTTCTTGAAGTGGTGGCCTAA
CTACGGCTACACTAGAAGGACAGTATTTGGTATCTGCGCTCTGCTGAAGC
CAGTTACCTTCGGAAAAAGAGTTGGTAGCTCTTGATCCGGCAAACAAACC
ACCGCTGGTAGCGGTGGTTTTTTTGTTTGCAAGCAGCAGATTACGCGCAG
AAAAAAAGGATCTCAAGAAGATCCTTTGATCTTTTCTACGGGGTCTGACG
CTCAGTGGAACGAAAACTCACGTTAAGGGATTTTGGTCATGAGATTATCA

AAAAGGATCTTCACCTAGATCCTTTTAAATTAAAAATGAAGTTTTAAATC
AATCTAAAGTATATATGAGTAAACTTGGTCTGACAGTTACCAATGCTTAA
TCAGTGAGGCACCTATCTCAGCGATCTGTCTATTTCGTTCATCCATAGTT
GCCTGACTCCCCGTCGTGTAGATAACTACGATACGGGAGGGCTTACCATC
TGGCCCCAGTGCTGCAATGATACCGCGAGACCCACGCTCACCGGCTCCAG
ATTTATCAGCAATAAACCAGCCAGCCGGAAGGGCCGAGCGCAGAAGTGGT
CCTGCAACTTTATCCGCCTCCATCCAGTCTATTAATTGTTGCCGGGAAGC
TAGAGTAAGTAGTTCGCCAGTTAATAGTTTGCGCAACGTTGTTGCCATTG
CTACAGGCATCGTGGTGTCACGCTCGTCGTTTGGTATGGCTTCATTCAGC
TCCGGTTCCCAACGATCAAGGCGAGTTACATGATCCCCCATGTTGTGCAA
AAAAGCGGTTAGCTCCTTCGGTCCTCCGATCGTTGTCAGAAGTAAGTTGG
CCGCAGTGTTATCACTCATGGTTATGGCAGCACTGCATAATTCTCTTACT
GTCATGCCATCCGTAAGATGCTTTTCTGTGACTGGTGAGTACTCAACCAA
GTCATTCTGAGAATAGTGTATGCGGCGACCGAGTTGCTCTTGCCCGGCGT
CAATACGGGATAATACCGCGCCACATAGCAGAACTTTAAAAGTGCTCATC
ATTGGAAAACGTTCTTCGGGGCGAAAACTCTCAAGGATCTTACCGCTGTT
GAGATCCAGTTCGATGTAACCCACTCGTGCACCCAACTGATCTTCAGCAT
CTTTTACTTTCACCAGCGTTTCTGGGTGAGCAAAAACAGGAAGGCAAAAT
GCCGCAAAAAAGGGAATAAGGGCGACACGGAAATGTTGAATACTCATACT
CTTCCTTTTTCAATATTATTGAAGCATTTATCAGGGTTATTGTCTCATGA
GCGGATACATATTTGAATGTATTTAGAAAAATAAACAAATAGGGGTTCCG
CGCACATTTCCCCGAAAGTGCCACCTGACGTCTAAGAAACCATTATTAT
CATGACATTAACCTATAAAAATAGGCGTATCACGAGGCCCTTTCGTC

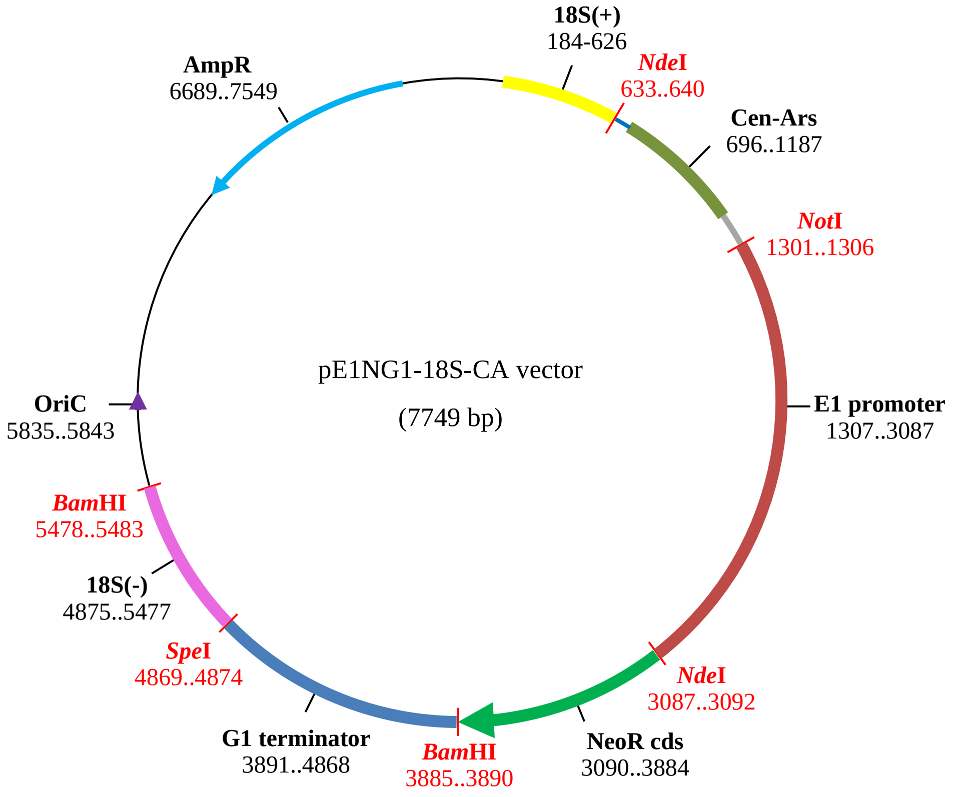

pE1NG1-18S-CA; 7749 bp

TCGCGCGTTTCGGTGATGACGGTGAAAACCTCTGACACATGCAGCTCCCG
GAGACGGTCACAGCTTGTCTGTAAGCGGATGCCGGGAGCAGACAAGCCCG
TCAGGGCGCGTCAGCGGGTGTTGGCGGGTGTCGGGGCTGGCTTAACTATG
CGGCATCAGAGCAGATTGTACTGAGAGTGCACCATATCGATACCTGCAGT
AATTCTGGAAATAATACATGCTGTAAGAGCCCTGTATGGGGCTGCACTTA
TTAGATTGAAGCCGATTTTATTGGTGAATCATGATAATTGAGCAGATTGA
CATATTTAGTCGATGAATCGTTTGAGTTTCTGCCCCATCAGTTGTCGACG
GTAGTGTATTGGACTACGGTGACTATAACGGGTGACGGAGAGTTAGGGCT
CGACTCCGGAGAGGGAGCCTGAGAGACGGCTACCATATCCAAGGATAGCA
GCAGGCGCGTAAATTACCCACTGTGGACTCCACGAGGTAGTGACGAGAAA
TATCGATGCGAAGCGTGTATGCGTTTTGCTATCGGAATGAGAGCAATGTA
AAACCCTCATCGAGGATCAACTGGAGGGCAAGTCTGGTGCCAGCAGCCGC
GGTAATTCCAGCTCCAGAAGCATATGGGGCCCGTTGCAGTCACTCCGCTT
TGGTTTCACAGTCAGGAATAACACTAGCTCGTCTTCAGCGAGCATCACGT
GCTATAAAAATAATTATAATTTAAATTTTTAATATAAATATATAAATTA
AAAATAGAAAGTAAAAAAAGAAATTAAAGAAAAAATAGTTTTTGTTTTCC

GAAGATGTAAAAGACTCTAGGGGGATCGCCAACAAATACTACCTTTTACC
TTGCTCTTCCTGCTCTCAGGTATTAATGCCGAATTGTTTCATCTTGTCTG
TGTAGAAGACCACACACGAAAATCCTGTGATTTTACATTTTACTTATCGT
TAATCGAATGTATATCTATTTAATCTGCTTTTCTTGTCTAATAAATATAT
ATGTAAAGTACGCTTTTTGTTGAAATTTTTTAAACCTTTGTTTATTTTTT
TTTCTTCATTCCGTAACTCTTCTACCTTCTTTATTTACTTTCTAAAATCC
AAATACAAAACATAAAAATAAATAAACACAGAGTAAATTCCCAAATTATT
CCATCATTAAAAGATACGAGGCGCGTGTAAGTTACAGGCAAGCGATCCTA
GTACACTCTATATTTTTTTATGCCTCGGTAATGATTTTCATTTTTTTTTT
CCACCTAGCGGATGACTCTTTTTTTTTCTTAGCGATTGGCATTATCACGC
GGCCGCCTAGCTAGCTCATGCTCCTTTCCCGCCAAAAAGAAAGAAGAGGA
AAGCACCCCGAAGAAAAGAAAGAAATCACCCAAACACCCTCCTCCTTCCT
CGTCCACAGACAGCTCAGAATAATAAAAGCTATCTTTCCATCGCTCTTGA
CCTAACTCTCTTTCTGCTCCTGTAAATTCATCCAACAAATGTTTAGTCTA
AGAAACCCATCTGCCTCATACTCCTACTTACTACCTTCCTTACTTGAAAG
CAGGCAGGCTCACGGCCAGCTTGGCAGATAGGATAGTTCTCATATCTATT
GCTGATCGTTCCCGTTTCTTTCTTAAAGCAAAGTCTTTTCTTTTCTTTTC
TTTTCTTTCCAGGCTCTCCACGTTTTCAGAAGTAGTACATTTTCTACTTA
GTAATTAGAAAGCTTAGTACTTTTTGCTTTTCTGGATTCTGAAGACTTGG
AAATAGAAAGAAATTAAAAATCTTTTTCTTCTTTCTTTCAGCCTTTGTTG
GACTCCCTCGCACGTCTCCTTCTCCCCCAGCCATCCATCAGCGGGCACTC
CACCCGCGCTTCAACGCTCGCTCGAGTGCGTGCTTATTTGCCTTCAACGC
GGCGCGGCGGTTAATATAGTCCCAGCACTCCTTAAGGGGGGCATCGCAGG
GATTATCATTTTAAAAACTGTCACGGAGTTACATCTTCCCTCGCATCAAA
GTGTTCCCGGCCGCGTCGCATATCTAAGTTTTATAACCTACACCCCTGGT
GGGGTAGGGGCGAATTCTATGTACACAGCACCTCAGAACTTGCGCGCGTT
CCGTGACAAATGAGGGGTGTGGCGGCGCATTCGGCCGCATCGCCACATTC
AGATATCTAACATACCCCCCCTTCGCGATGGGTGGCAGGCGAGGCGGATT
CGCTCGCGAGAGGCGAGGTGCCACAGCAGACCAGTAACGAGGAGCCAAGG
TAGGTGACCACCGACGACTACGACCACGACCACAGCCACGGCGGCTGCAG
CCACGGGACGCCTCGCATGGCAGCGCAGCAGCACCAGCAACGACTGCTAC
AAGGAGTGCAGGGCCGATCTAGACGCGCCGGAGCCGCACGACCAATGCCG
ACGCAACGCTGATTCTTCTGGATTCCCTCTATACATTCATATATATGAAG
AGAAGCGGATGAGACGGCCTGCGAATAAATGAATGGCTTGGAGTTTGCTT
GCTTGCTGTATGCTCGAAGTGCGTGTGCAGACACAGGCACGACCGAGAGG
ACAACAGTCTGTGCTTACCTCACCAGCACATTCTTGCAACGCCATTCGAA
GCACGCGAAATCTTGTGGCTCAGAGCAAAAGGCATCCGTGGTACGGAAC
GTGGGGAGCGCTATCAATTTGGAATTCAAAATGAGTGAACCAGACAACTA

ACTGTGACTTGAACTGTTGCTCCACGCATCAAAACCAAACCCTTAACAGA
AGTAGACCAGTTCGAAGCTACTAGCACCAAACAAAATGGGCAAGACGAAG
GAGCACGTCAACCTTGTCGTCATCGGCCACGTCGATGCCGGCAAGTCCAC
CACCACCGGCCACTTGATCTACAAGTGCGGTGGTATCGACAAGCGTACCA
TCGAGAAGTTCGAGAAGGAGGCCGCCGAGCTCGGTAAGGGTTCCTTCAAG
TACGCATGGGTTCTTGACAAGCTCAAGGCCGAGCGTGAGCGTGGTATCAC
CATCGATATCGCCTCTGGAAGTTCGAGTCCCCCAAGTTCGACTTCACCGT
CATCGATGCCCCCGGTCACCGTGATTTCATCAAGAACATATGATTGAACA
AGATGGATTGCACGCAGGTTCTCCGGCCGCTTGGGTGGAGAGGCTATTCG
GCTATGACTGGGCACAACAGACAATCGGCTGCTCTGATGCCGCCGTGTTC
CGGCTGTCAGCGCAGGGGCGCCCGGTTCTTTTTGTCAAGACCGACCTGTC
CGGTGCCCTGAATGAACTGCAAGACGAGGCAGCGCGGCTATCGTGGCTGG
CCACGACGGGCGTTCCCTGCGCAGCTGTGCTCGACGTTGTCACTGAAGCG
GGAAGGGACTGGCTGCTATTGGGCGAAGTGCCGGGGCAGGATCTCCTGTC
ATCTCACCTTGCTCCTGCCGAGAAAGTATCCATCATGGCTGATGCAATGC
GGCGGCTGCATACGCTTGATCCGGCTACCTGCCCATTCGACCACCAAGCG
AAACATCGCATCGAGCGAGCACGTACTCGGATGGAAGCCGGTCTTGTCGA
TCAGGATGATCTGGACGAAGAGCATCAGGGGCTCGCGCCAGCCGAACTGT
TCGCCAGGCTCAAGGCGAGCATGCCCGACGGCGAGGATCTCGTCGTGACC
CATGGCGATGCCTGCTTGCCGAATATCATGGTGGAAAATGGCCGCTTTTC
TGGATTCATCGACTGTGGCCGGCTGGGTGTGGCGGACCGCTATCAGGACA
TAGCGTTGGCTACCCGTGATATTGCTGAAGAGCTTGGCGGCGAATGGGCT
GACCGCTTCCTCGTGCTTTACGGTATCGCCGCTCCCGATTCGCAGCGCAT
CGCCTTCTATCGCCTTCTTGACGAGTTCTTCTAAGGATCCATGTACCCAA
TACCACACCGGTAGCTTCTCGCGGCGGCTGACAAGAAAGATTGTTTTTAC
ACATTTCGAGGCATTAATGACCCTTATCGACCTATCGTCTCAGATCATAA
AATGCACGAGCATGAAGTACGCGTGTTGTGACTTGCTTGCGTCCCACCTT
CTAGATGGCTTTCTTCTTTTCAAGTGATTAAAACACCACAGTAGATGAGG
ACTCTTAGTAAGCACTGAAGAAGCGAGTAGATAGCCCTCATCCCGTTTCC
CTCTTTTCTAACACACTTTGTTTGGAATTCTAAAATATCTTTATCATCTC
TTTTCATTCACAAAACTAGTATTTCTGCATTAGAAATCATTATCCTATCT
GGCACTTTCACTCTGACAAGAACTTGCGTACATGGCGGGTCCTGGCAATC
ATTTTACTTGTACAGACCCAAGACTTTGTGAGTCAGTAAATAGTAAAGAA
ATGCAGAATAATCATTAGATAATTGCAAAACCCTGATCTTCAAAATGTTA
TATCACAAGTACCTACCAAGACATTTGTATCTTCTTTTTGTCTTATGCAT
TTTTATTTCCCATAGCGCAATTAAAAATATTAAATAACTGACGACTCTTT
TCTTAAGGATTCGCGATTTGCTAGTTGCACTATTGAAAAGAGCACAGTAT
TAATTTCATAGTTTTACTTTGTTGGCAATCTCAGCAAACATGGGTTTTAA

TTTTAAAATTAAATAATATTTGTGTTAGACTCAAAAGATACCTATACAAC
CCTTAGGGCTCATCGTCAACATTTGCAAATTAAAACGATTCAGCTCCTTT
GGTATGGTCATGTCTGTTTCTGATGCACTGAAAAATCTTGGACGAACGAC
ACATTTTATTTTCATTAGGGCATCTAAAAAACGTTGTAAAGAGTATACAA
TAAGCAAAAAAAAGGAGAGAAAAAAAAAGGACATATATGGTTCTGCTTTT
CTAGTGCTTCAAGTTTGTACTAGTCTGGGGATCGAAGATGATTAGATACC
ATCGTAGTCTAGACCGTAAACGATGCCGACTTGCGATTGTTGGGTGCTTT
ATTACATGGGCCTCAGCAGCAGCACATGAGAAATCAAAGTCTTTGGGTTC
CGGGGGGAGTATGGTCGCAAGGCTGAAACTTAAAGGAATTGACGGAAGGG
CACCACCAGGAGTGGAGCCTGCGGCTTAATTTGACTCAACACGGGAAAAC
TTACCAGGTCCAGACATAGGTAGGATTGACAGATTGAGAGCTCTTTCATG
ATTCTATGGGTGGTGGTGCATGGCCGTTCTTAGTTGGTGGAGTGATTTGT
CTGGTTAATTCCGTTAACGAACGAGACCTCGGCCTACTAAATAGTGCGTG
GTATGGCAACATAGTACGTTTTTAACTTCTTAGAGGGACATGTCCGGTTT
ACGGGCAGGAAGTTCGAGGCAATAACAGGTCTGTGATGCCCTTAGATGTT
CTGGGCCGCACGCGCGCTACACTGATGGGTTCATCGGGTTTTAAATTCAA
TTTTTGGAATTGAGTGCTTGGTCGGAAGGCCTGGCTAATCCTTGGAACGC
TCATCGTGCTGGGGCTAGATTTTTGCAGGATCCCCTCTAGAGTCGACCTG
CAGGCATGCAAGCTTGGCGTAATCATGGTCATAGCTGTTTCCTGTGTGAA
ATTGTTATCCGCTCACAATTCCACACAACATACGAGCCGGAAGCATAAAG
TGTAAAGCCTGGGGTGCCTAATGAGTGAGCTAACTCACATTAATTGCGTT
GCGCTCACTGCCCGCTTTCCAGTCGGGAAACCTGTCGTGCCAGCTGCATT
AATGAATCGGCCAACGCGCGGGGAGAGGCGGTTTGCGTATTGGGCGCTCT
TCCGCTTCCTCGCTCACTGACTCGCTGCGCTCGGTCGTTCGGCTGCGGCG
AGCGGTATCAGCTCACTCAAAGGCGGTAATACGGTTATCCACAGAATCAG
GGGATAACGCAGGAAAGAACATGTGAGCAAAAGGCCAGCAAAAGGCCAGG
AACCGTAAAAAGGCCGCGTTGCTGGCGTTTTTCCATAGGCTCCGCCCCCC
TGACGAGCATCACAAAAATCGACGCTCAAGTCAGAGGTGGCGAAACCCGA
CAGGACTATAAAGATACCAGGCGTTTCCCCCTGGAAGCTCCCTCGTGCGC
TCTCCTGTTCCGACCCTGCCGCTTACCGGATACCTGTCCGCCTTTCTCCC
TTCGGGAAGCGTGGCGCTTTCTCATAGCTCACGCTGTAGGTATCTCAGTT
CGGTGTAGGTCGTTCGCTCCAAGCTGGGCTGTGTGCACGAACCCCCCGTT
CAGCCCGACCGCTGCGCCTTATCCGGTAACTATCGTCTTGAGTCCAACCC
GGTAAGACACGACTTATCGCCACTGGCAGCAGCCACTGGTAACAGGATTA
GCAGAGCGAGGTATGTAGGCGGTGCTACAGAGTTCTTGAAGTGGTGGCCT
AACTACGGCTACACTAGAAGGACAGTATTTGGTATCTGCGCTCTGCTGAA
GCCAGTTACCTTCGGAAAAAGAGTTGGTAGCTCTTGATCCGGCAAACAAA
CCACCGCTGGTAGCGGTGGTTTTTTTGTTTGCAAGCAGCAGATTACGCGC

AGAAAAAAAGGATCTCAAGAAGATCCTTTGATCTTTTCTACGGGGTCTGA
CGCTCAGTGGAACGAAAACTCACGTTAAGGGATTTTGGTCATGAGATTAT
CAAAAAGGATCTTCACCTAGATCCTTTTAAATTAAAAATGAAGTTTTAAA
TCAATCTAAAGTATATATGAGTAAACTTGGTCTGACAGTTACCAATGCTT
AATCAGTGAGGCACCTATCTCAGCGATCTGTCTATTTCGTTCATCCATAG
TTGCCTGACTCCCCGTCGTGTAGATAACTACGATACGGGAGGGCTTACCA
TCTGGCCCCAGTGCTGCAATGATACCGCGAGACCCACGCTCACCGGCTCC
AGATTTATCAGCAATAAACCAGCCAGCCGGAAGGGCCGAGCGCAGAAGTG
GTCCTGCAACTTTATCCGCCTCCATCCAGTCTATTAATTGTTGCCGGGAA
GCTAGAGTAAGTAGTTCGCCAGTTAATAGTTTGCGCAACGTTGTTGCCAT
TGCTACAGGCATCGTGGTGTCACGCTCGTCGTTTGGTATGGCTTCATTCA
GCTCCGGTTCCCAACGATCAAGGCGAGTTACATGATCCCCCATGTTGTGC
AAAAAAGCGGTTAGCTCCTTCGGTCCTCCGATCGTTGTCAGAAGTAAGTT
GGCCGCAGTGTTATCACTCATGGTTATGGCAGCACTGCATAATTCTCTTA
CTGTCATGCCATCCGTAAGATGCTTTTCTGTGACTGGTGAGTACTCAACC
AAGTCATTCTGAGAATAGTGTATGCGGCGACCGAGTTGCTCTTGCCCGGC
GTCAATACGGGATAATACCGCGCCACATAGCAGAACTTTAAAAGTGCTCA
TCATTGGAAAACGTTCTTCGGGGCGAAAACTCTCAAGGATCTTACCGCTG
TTGAGATCCAGTTCGATGTAACCCACTCGTGCACCCAACTGATCTTCAGC
ATCTTTTACTTTCACCAGCGTTTCTGGGTGAGCAAAAACAGGAAGGCAAA
ATGCCGCAAAAAAGGGAATAAGGGCGACACGGAAATGTTGAATACTCATA
CTCTTCCTTTTTCAATATTATTGAAGCATTTATCAGGGTTATTGTCTCAT
GAGCGGATACATATTTGAATGTATTTAGAAAAATAAACAAATAGGGGTTC
CGCGCACATTTCCCCGAAAAGTGCCACCTGACGTCTAAGAAACCATTATT
ATCATGACATTAACCTATAAAAATAGGCGTATCACGAGGCCCTTTCGTC

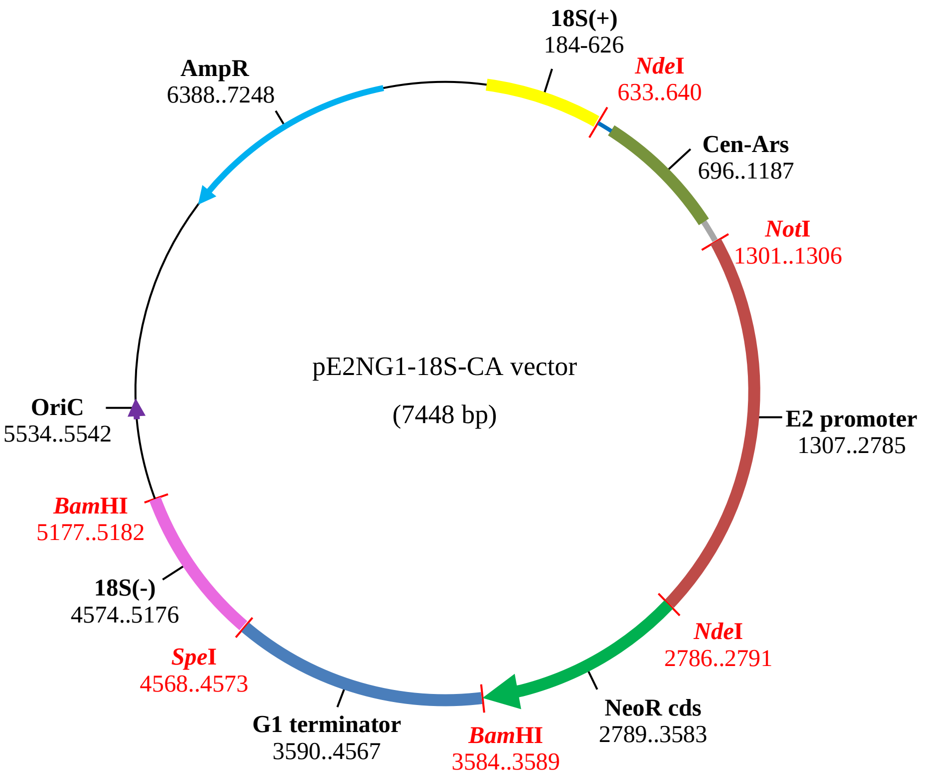

pE2NG1-18S-CA; 7448 bp

TCGCGCGTTTCGGTGATGACGGTGAAAACCTCTGACACATGCAGCTCCCG
GAGACGGTCACAGCTTGTCTGTAAGCGGATGCCGGGAGCAGACAAGCCCG
TCAGGGCGCGTCAGCGGGTGTTGGCGGGTGTCGGGGCTGGCTTAACTATG
CGGCATCAGAGCAGATTGTACTGAGAGTGCACCATATCGATACCTGCAGT
AATTCTGGAAATAATACATGCTGTAAGAGCCCTGTATGGGGCTGCACTTA
TTAGATTGAAGCCGATTTTATTGGTGAATCATGATAATTGAGCAGATTGA
CATATTTAGTCGATGAATCGTTTGAGTTTCTGCCCCATCAGTTGTCGACG
GTAGTGTATTGGACTACGGTGACTATAACGGGTGACGGAGAGTTAGGGCT
CGACTCCGGAGAGGGAGCCTGAGAGACGGCTACCATATCCAAGGATAGCA
GCAGGCGCGTAAATTACCCACTGTGGACTCCACGAGGTAGTGACGAGAAA
TATCGATGCGAAGCGTGTATGCGTTTTGCTATCGGAATGAGAGCAATGTA
AAACCCTCATCGAGGATCAACTGGAGGGCAAGTCTGGTGCCAGCAGCCGC
GGTAATTCCAGCTCCAGAAGCATATGGGGCCCGTTGCAGTCACTCCGCTT
TGGTTTCACAGTCAGGAATAACACTAGCTCGTCTTCAGCGAGCATCACGT
GCTATAAAAATAATTATAATTTAAATTTTTTAATATAAATATATAAATTA
AAAATAGAAAGTAAAAAAAGAAATTAAAGAAAAAATAGTTTTTGTTTTCC

GAAGATGTAAAAGACTCTAGGGGGATCGCCAACAAATACTACCTTTTACC
TTGCTCTTCCTGCTCTCAGGTATTAATGCCGAATTGTTTCATCTTGTCTG
TGTAGAAGACCACACACGAAAATCCTGTGATTTTACATTTTACTTATCGT
TAATCGAATGTATATCTATTTAATCTGCTTTTCTTGTCTAATAAATATAT
ATGTAAAGTACGCTTTTTGTTGAAATTTTTTAAACCTTTGTTTATTTTTT
TTTCTTCATTCCGTAACTCTTCTACCTTCTTTATTTACTTTCTAAAATCC
AAATACAAAACATAAAAATAAATAAACACAGAGTAAATTCCCAAATTATT
CCATCATTAAAAGATACGAGGCGCGTGTAAGTTACAGGCAAGCGATCCTA
GTACACTCTATATTTTTTTATGCCTCGGTAATGATTTTCATTTTTTTTTT
CCACCTAGCGGATGACTCTTTTTTTTTCTTAGCGATTGGCATTATCACGC
GGCCGCCTAGCTAGCTCATGCTCCTTTCCCGCCAAAAAGAAAGAAGAGGA
AAGCACCCCGAAGAAAAGAAAGAAATCACCCAAACACCCTCCTCCTTCCT
CGTCCACAGACAGCTCAGAATAATAAAAGCTATCTTTCCATCGCTCTTGA
CCTAACTCTCTTTCTGCTCCTGTAAATTCATCCAACAAATGTTTAGTCTA
AGAAACCCATCTGCCTCATACTCCTACTTACTACCTTCCTTACTTGAAAG
CAGGCAGGCTCACGGCCAGCTTGGCAGATAGGATAGTTCTCATATCTATT
GCTGATCGTTCCCGTTTCTTTCTTAAAGCAAAGTCTTTTCTTTTCTTTTC
TTTTCTTTCCAGGCTCTCCACGTTTTCAGAAGTAGTACATTTTCTACTTA
GTAATTAGAAAGCTTAGTACTTTTTGCTTTTCTGGATTCTGAAGACTTGG
AAATAGAAAGAAATTAAAAATCTTTTTCTTCTTTCTTTCAGCCTTTGTTG
GACTCCCTCGCACGTCTCCTTCTCCCCCAGCCATCCATCAGCGGGCACTC
CACCCGCGCTTCAACGCTCGCTCGAGTGCGTGCTTATTTGCCTTCAACGC
GGCGCGGCGGTTAATATAGTCCCAGCACTCCTTAAGGGGGGCATCGCAGG
GATTATCATTTTAAAAACTGTCACGGAGTTACATCTTCCCTCGCATCAAA
GTGTTCCCGGCCGCGTCGCATATCTAAGTTTTATAACCTACACCCCTGGT
GGGGTAGGGGCGAATTCTATGTACACAGCACCTCAGAACTTGCGCGCGTT
CCGTGACAAATGAGGGGTGTGGCGGCGCATTCGGCCGCATCGCCACATTC
AGATATCTAACATACCCCCCCTTCGCGATGGGTGGCAGGCGAGGCGGATT
CGCTCGCGAGAGGCGAGGTGCCACAGCAGACCAGTAACGAGGAGCCAAGG
TAGGTGACCACCGACGACTACGACCACGACCACAGCCACGGCGGCTGCAG
CCACGGGACGCCTCGCATGGCAGCGCAGCAGCACCAGCAACGACTGCTAC
AAGGAGTGCAGGGCCGATCTAGACGCGCCGGAGCCGCACGACCAATGCCG
ACGCAACGCTGATTCTTCTGGATTCCCTCTATACATTCATATATATGAAG
AGAAGCGGATGAGACGGCCTGCGAATAAATGAATGGCTTGGAGTTTGCTT
GCTTGCTGTATGCTCGAAGTGCGTGTGCAGACACAGGCACGACCGAGAGG
ACAACAGTCTGTGCTTACCTCACCAGCACATTCTTGCAACGCCATTCGAA
GCACGCGAAATCTTGTGGCTCAGAGCAAAAGGCATCCGTGGTACGGAAC
GTGGGGAGCGCTATCAATTTGGAATTCAAAATGAGTGAACCAGACAACTA

ACTGTGACTTGAACTGTTGCTCCACGCATCAAAACCAAACCCTTAACAGA
AGTAGACCAGTTCGAAGCTACTAGCACCAAACAAACATATGATTGAACAA
GATGGATTGCACGCAGGTTCTCCGGCCGCTTGGGTGGAGAGGCTATTCGG
CTATGACTGGGCACAACAGACAATCGGCTGCTCTGATGCCGCCGTGTTCC
GGCTGTCAGCGCAGGGGCGCCCGGTTCTTTTTGTCAAGACCGACCTGTCC
GGTGCCCTGAATGAACTGCAAGACGAGGCAGCGCGGCTATCGTGGCTGGC
CACGACGGGCGTTCCCTGCGCAGCTGTGCTCGACGTTGTCACTGAAGCGG
GAAGGGACTGGCTGCTATTGGGCGAAGTGCCGGGGCAGGATCTCCTGTCA
TCTCACCTTGCTCCTGCCGAGAAAGTATCCATCATGGCTGATGCAATGCG
GCGGCTGCATACGCTTGATCCGGCTACCTGCCCATTCGACCACCAAGCGA
AACATCGCATCGAGCGAGCACGTACTCGGATGGAAGCCGGTCTTGTCGAT
CAGGATGATCTGGACGAAGAGCATCAGGGGCTCGCGCCAGCCGAACTGTT
CGCCAGGCTCAAGGCGAGCATGCCCGACGGCGAGGATCTCGTCGTGACCC
ATGGCGATGCCTGCTTGCCGAATATCATGGTGGAAAATGGCCGCTTTTCT
GGATTCATCGACTGTGGCCGGCTGGGTGTGGCGGACCGCTATCAGGACAT
AGCGTTGGCTACCCGTGATATTGCTGAAGAGCTTGGCGGCGAATGGGCTG
ACCGCTTCCTCGTGCTTTACGGTATCGCCGCTCCCGATTCGCAGCGCATC
GCCTTCTATCGCCTTCTTGACGAGTTCTTCTAAGGATCCATGTACCCAAT
ACCACACCGGTAGCTTCTCGCGGCGGCTGACAAGAAAGATTGTTTTTACA
CATTTCGAGGCATTAATGACCCTTATCGACCTATCGTCTCAGATCATAAA
ATGCACGAGCATGAAGTACGCGTGTTGTGACTTGCTTGCGTCCCACCTTC
TAGATGGCTTTCTTCTTTTCAAGTGATTAAAACACCACAGTAGATGAGGA
CTCTTAGTAAGCACTGAAGAAGCGAGTAGATAGCCCTCATCCCGTTTCCC
TCTTTTCTAACACACTTTGTTTGGAATTCTAAAATATCTTTATCATCTCT
TTTCATTCACAAAACTAGTATTTCTGCATTAGAAATCATTATCCTATCTG
GCACTTTCACTCTGACAAGAACTTGCGTACATGGCGGGTCCTGGCAATCA
TTTTACTTGTACAGACCCAAGACTTTGTGAGTCAGTAAATAGTAAAGAAA
TGCAGAATAATCATTAGATAATTGCAAAACCCTGATCTTCAAAATGTTAT
ATCACAAGTACCTACCAAGACATTTGTATCTTCTTTTTGTCTTATGCATT
TTTATTTCCCATAGCGCAATTAAAAATATTAAATAACTGACGACTCTTTT
CTTAAGGATTCGCGATTTGCTAGTTGCACTATTGAAAAGAGCACAGTATT
AATTTCATAGTTTTACTTTGTTGGCAATCTCAGCAAACATGGGTTTTAAT
TTTAAAATTAAATAATATTTGTGTTAGACTCAAAAGATACCTATACAACC
CTTAGGGCTCATCGTCAACATTTGCAAATTAAAACGATTCAGCTCCTTTG
GTATGGTCATGTCTGTTTCTGATGCACTGAAAAATCTTGGACGAACGACA
CATTTTATTTTCATTAGGGCATCTAAAAAACGTTGTAAAGAGTATACAAT
AAGCAAAAAAAAAGGAGAGAAAAAAAAAGGACATATATGGTTCTGCTTTTC
TAGTGCTTCAAGTTTGTACTAGTCTGGGGATCGAAGATGATTAGATACCA

TCGTAGTCTAGACCGTAAACGATGCCGACTTGCGATTGTTGGGTGCTTTA
TTACATGGCCTCAGCAGCAGCACATGAGAAATCAAAGTCTTTGGGTTCC
GGGGGGAGTATGGTCGCAAGGCTGAAACTTAAAGGAATTGACGGAAGGGC
ACCACCAGGAGTGGAGCCTGCGGCTTAATTTGACTCAACACGGAAAACT
TACCAGGTCCAGACATAGGTAGGATTGACAGATTGAGAGCTCTTTCATGA
TTCTATGGGTGGTGGTGCATGGCCGTTCTTAGTTGGTGGAGTGATTTGTC
TGGTTAATTCCGTTAACGAACGAGACCTCGGCCTACTAAATAGTGCGTGG
TATGGCAACATAGTACGTTTTTAACTTCTTAGAGGACATGTCCGGTTTA
CGGGCAGGAAGTTCGAGGCAATAACAGGTCTGTGATGCCCTTAGATGTTC
TGGGCCGCACGCGCGCTACACTGATGGGTTCATCGGGTTTTAAATTCAAT
TTTTGGAATTGAGTGCTTGGTCGGAAGGCCTGGCTAATCCTTGGAACGCT
CATCGTGCTGGGGCTAGATTTTTGCAGGATCCCCTCTAGAGTCGACCTGC
AGGCATGCAAGCTTGGCGTAATCATGGTCATAGCTGTTTCCTGTGTGAAA
TTGTTATCCGCTCACAATTCCACACAACATACGAGCCGGAAGCATAAAGT
GTAAAGCCTGGGGTGCCTAATGAGTGAGCTAACTCACATTAATTGCGTTG
CGCTCACTGCCCGCTTTCCAGTCGGGAAACCTGTCGTGCCAGCTGCATTA
ATGAATCGGCCAACGCGCGGGGAGAGGCGGTTTGCGTATTGGGCGCTCTT
CCGCTTCCTCGCTCACTGACTCGCTGCGCTCGGTCGTTCGGCTGCGGCGA
GCGGTATCAGCTCACTCAAAGGCGGTAATACGGTTATCCACAGAATCAGG
GGATAACGCAGGAAAGAACATGTGAGCAAAAGGCCAGCAAAAGGCCAGGA
ACCGTAAAAAGGCCGCGTTGCTGGCGTTTTTCCATAGGCTCCGCCCCCCT
GACGAGCATCACAAAAATCGACGCTCAAGTCAGAGGTGGCGAAACCCGAC
AGGACTATAAAGATACCAGGCGTTTCCCCCTGGAAGCTCCCTCGTGCGCT
CTCCTGTTCCGACCCTGCCGCTTACCGGATACCTGTCCGCCTTTCTCCCT
TCGGGAAGCGTGGCGCTTTCTCATAGCTCACGCTGTAGGTATCTCAGTTC
GGTGTAGGTCGTTCGCTCCAAGCTGGGCTGTGTGCACGAACCCCCCGTTC
AGCCCGACCGCTGCGCCTTATCCGGTAACTATCGTCTTGAGTCCAACCCG
GTAAGACACGACTTATCGCCACTGGCAGCAGCCACTGGTAACAGGATTAG
CAGAGCGAGGTATGTAGGCGGTGCTACAGAGTTCTTGAAGTGGTGGCCTA
ACTACGGCTACACTAGAAGGACAGTATTTGGTATCTGCGCTCTGCTGAAG
CCAGTTACCTTCGGAAAAAGAGTTGGTAGCTCTTGATCCGGCAAACAAAC
CACCGCTGGTAGCGGTGGTTTTTTTGTTTGCAAGCAGCAGATTACGCGCA
GAAAAAAAGGATCTCAAGAAGATCCTTTGATCTTTTCTACGGGGTCTGAC
GCTCAGTGGAACGAAAACTCACGTTAAGGGATTTTGGTCATGAGATTATC
AAAAAGGATCTTCACCTAGATCCTTTTAAATTAAAAATGAAGTTTTAAAT
CAATCTAAAGTATATATGAGTAAACTTGGTCTGACAGTTACCAATGCTTA
ATCAGTGAGGCACCTATCTCAGCGATCTGTCTATTTCGTTCATCCATAGT
TGCCTGACTCCCCGTCGTGTAGATAACTACGATACGGGAGGGCTTACCAT

CTGGCCCCAGTGCTGCAATGATACCGCGAGACCCACGCTCACCGGCTCCA
GATTTATCAGCAATAAACCAGCCAGCCGGAAGGGCCGAGCGCAGAAGTGG
TCCTGCAACTTTATCCGCCTCCATCCAGTCTATTAATTGTTGCCGGGAAG
CTAGAGTAAGTAGTTCGCCAGTTAATAGTTTGCGCAACGTTGTTGCCATT
GCTACAGGCATCGTGGTGTCACGCTCGTCGTTTGGTATGGCTTCATTCAG
CTCCGGTTCCCAACGATCAAGGCGAGTTACATGATCCCCCATGTTGTGCA
AAAAAGCGGTTAGCTCCTTCGGTCCTCCGATCGTTGTCAGAAGTAAGTTG
GCCGCAGTGTTATCACTCATGGTTATGGCAGCACTGCATAATTCTCTTAC
TGTCATGCCATCCGTAAGATGCTTTTCTGTGACTGGTGAGTACTCAACCA
AGTCATTCTGAGAATAGTGTATGCGGCGACCGAGTTGCTCTTGCCCGGCG
TCAATACGGGATAATACCGCGCCACATAGCAGAACTTTAAAAGTGCTCAT
CATTGGAAAACGTTCTTCGGGGCGAAAACTCTCAAGGATCTTACCGCTGT
TGAGATCCAGTTCGATGTAACCCACTCGTGCACCCAACTGATCTTCAGCA
TCTTTTACTTTCACCAGCGTTTCTGGGTGAGCAAAAACAGGAAGGCAAAA
TGCCGCAAAAAAGGGAATAAGGGCGACACGGAAATGTTGAATACTCATAC
TCTTCCTTTTTCAATATTATTGAAGCATTTATCAGGGTTATTGTCTCATG
AGCGGATACATATTTGAATGTATTTAGAAAAATAAACAAATAGGGGTTCC
GCGCACATTTCCCCGAAAAGTGCCACCTGACGTCTAAGAAACCATTATTA
TCATGACATTA

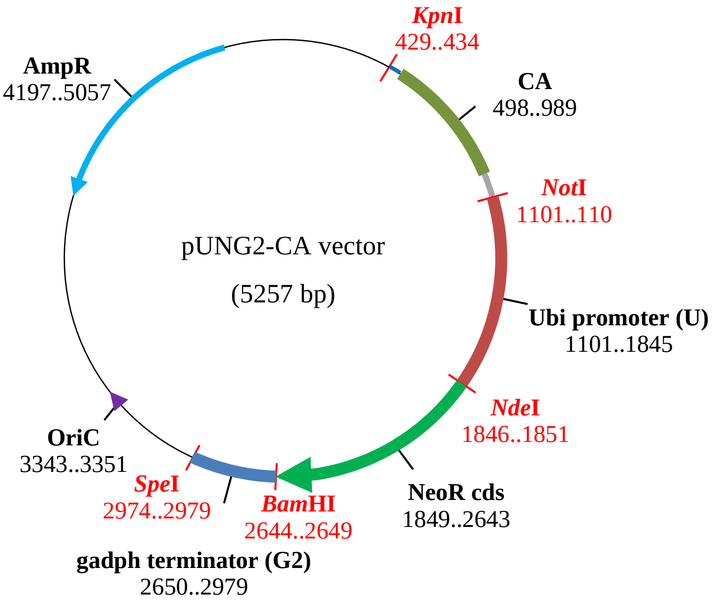

pUNG2-CA; 5257 bp

TCGCGCGTTTCGGTGATGACGGTGAAAACCTCTGACACATGCAGCTCCCG
GAGACGGTCACAGCTTGTCTGTAAGCGGATGCCGGGAGCAGACAAGCCCG
TCAGGGCGCGTCAGCGGGTGTTGGCGGGTGTCGGGGCTGGCTTAACTATG
CGGCATCAGAGCAGATTGTACTGAGAGTGCACCATATGCGGTGTGAAATA
CCGCACAGATGCGTAAGGAGAAAATACCGCATCAGGCGCCATTCGCCATT
CAGGCTGCGCAACTGTTGGGAAGGGCGATCGGTGCGGGCCTCTTCGCTAT
TACGCCAGCTGGCGAAAGGGGGATGTGCTGCAAGGCGATTAAGTTGGGTA
ACGCCAGGGTTTTCCCAGTCACGACGTTGTAAAACGACGGCCAGTGAATT
GTAATACGACTCACTATAGGGCGAGCTCGGTACCGTTGCAGTCACTCCGC
TTTGGTTTCACAGTCAGGAATAACACTAGCTCGTCTTCAGCGAGCATCAC
GTGCTATAAAAATAATTATAATTTAAATTTTTTAATATAAATATATAAAT
TAAAAATAGAAAGTAAAAAAAGAAATTAAAGAAAAAATAGTTTTTGTTTT
CCGAAGATGTAAAAGACTCTAGGGGGATCGCCAACAAATACTACCTTTTA
CCTTGCTCTTCCTGCTCTCAGGTATTAATGCCGAATTGTTTCATCTTGTC
TGTGTAGAAGACCACACACGAAAATCCTGTGATTTTACATTTTACTTATC
GTTAATCGAATGTATATCTATTTAATCTGCTTTTCTTGTCTAATAAATAT
ATATGTAAAGTACGCTTTTTGTTGAAATTTTTTAAACCTTTGTTTATTTT
TTTTTCTTCATTCCGTAACTCTTCTACCTTCTTTATTTACTTTCTAAAAT
CCAAATACAAAACATAAAAATAAATAAACACAGAGTAAATTCCCAAATTA
TTCCATCATTAAAAGATACGAGGCGCGTGTAAGTTACAGGCAAGCGATCC
TAGTACACTCTATATTTTTTTATGCCTCGGTAATGATTTTCATTTTTTTT

TTCCACCTAGCGGATGACTCTTTTTTTTTCTTAGCGATTGGCATTATCAC
GCGGCCGCGGGAGACTTTGAAAGGCACAGTAAGTTTTTGAAAGATAAAAG
GGATAAAGATCAAAAAGGTCATCCTGGCAGACATGTGGAAATCCAAGAGC
ACTGAAAACGCGAAGGTTCTCAGAGGTCTCTGCTCCATGTTCATGGCAGT
CCGAACGGCTTTGCCTAAGACTCGGTACTATGCTACAGCTCCAGAAGTCA
GTCAGTCAGTCTGTCTGAGCAAAATGAGCGAATTTTTAAATCCTTTGCGA
AGCTTTTTCGTTTGAAAGTTGTTTCTTTAAAATGGAAAGTTGATTGATTG
ACCCCGATCACTGAAGCTTTATCGCAAGAGGCAGAGAGAACAGACGGTGC
GAGATGGGTATTATTGTAAACGCTATGCGCGCAATAGCATCCAATCAAGA
GCGCAATCGAGCAACAGACGGAGATCAAAAAATAAGATAATAAAAATAAA
ACTGACTATATGTAGGAGTGGTTATGGATTTTGCTAAGATACTGGCGATT
GTGTTGAATTGAAAAGAAAGAAATAGAAGAAGAAGAAGAGAGAACAA
TGCTTTTATTTTACAAAGCAGCAGCTTACTTTAAAGTGCTTTTCATGGAC
TGAGCAGAATCACTCGAGGCGCAGCAAAAACCCAACTCCAGCCAGGCCAG
TCCAGACCGAAGAAAGCAAGCGTAGGGAGCTCAGCTTGATCCTGGTGCTG
TTCATTGAGATTGACAAAGTTGTAAGAATTAGATAAGCAAACAACCATAT
GATTGAACAAGATGGATTGCACGCAGGTTCTCCGGCCGCTTGGGTGGAGA
GGCTATTCGGCTATGACTGGGCACAACAGACAATCGGCTGCTCTGATGCC
GCCGTGTTCCGGCTGTCAGCGCAGGGGCGCCCGGTTCTTTTTGTCAAGAC
CGACCTGTCCGGTGCCCTGAATGAACTGCAAGACGAGGCAGCGCGGCTAT
CGTGGCTGGCCACGACGGGCGTTCCCTGCGCAGCTGTGCTCGACGTTGTC
ACTGAAGCGGGAAGGGACTGGCTGCTATTGGGCGAAGTGCCGGGGCAGGA
TCTCCTGTCATCTCACCTTGCTCCTGCCGAGAAAGTATCCATCATGGCTG
ATGCAATGCGGCGGCTGCATACGCTTGATCCGGCTACCTGCCCATTCGAC
CACCAAGCGAAACATCGCATCGAGCGAGCACGTACTCGGATGGAAGCCGG
TCTTGTCGATCAGGATGATCTGGACGAAGAGCATCAGGGGCTCGCGCCAG
CCGAACTGTTCGCCAGGCTCAAGGCGAGCATGCCCGACGGCGAGGATCTC
GTCGTGACCCATGGCGATGCCTGCTTGCCGAATATCATGGTGGAAAATGG
CCGCTTTTCTGGATTCATCGACTGTGGCCGGCTGGGTGTGGCGGACCGCT
ATCAGGACATAGCGTTGGCTACCCGTGATATTGCTGAAGAGCTTGGCGGC
GAATGGGCTGACCGCTTCCTCGTGCTTTACGGTATCGCCGCTCCCGATTC
GCAGCGCATCGCCTTCTATCGCCTTCTTGACGAGTTCTTCTAAGGATCCA
TGTACCCAATACCACACCGGTAGCTTCTCGCGGCGGCTGACAAGAAAGAT
TGTTTTTACACATTTCGAGGCATTAATGACCCTTATCGACCTATCGTCTC
AGATCATAAAATGCACGAGCATGAAGTACGCGTGTTGTGACTTGCTTGCG
TCCCACCTTCTAGATGGCTTTCTTCTTTTCAAGTGATTAAAACACCACAG
TAGATGAGGACTCTTAGTAAGCACTGAAGAAGCGAGTAGATAGCCCTCAT
CCCGTTTCCCTCTTTTCTAACACACTTTGTTTGGAATTCTAAAATATCTT

TATCATCTCTTTTCATTCACAAAACTAGTAGATCTGGATCCCCTCTAGAG
TCGACCTGCAGGCATGCAAGCTTGGCGTAATCATGGTCATAGCTGTTTCC
TGTGTGAAATTGTTATCCGCTCACAATTCCACACAACATACGAGCCGGAA
GCATAAAGTGTAAAGCCTGGGGTGCCTAATGAGTGAGCTAACTCACATTA
ATTGCGTTGCGCTCACTGCCCGCTTTCCAGTCGGGAAACCTGTCGTGCCA
GCTGCATTAATGAATCGGCCAACGCGCGGGGAGAGGCGGTTTGCGTATTG
GGCGCTCTTCCGCTTCCTCGCTCACTGACTCGCTGCGCTCGGTCGTTCGG
CTGCGGCGAGCGGTATCAGCTCACTCAAAGGCGGTAATACGGTTATCCAC
AGAATCAGGGGATAACGCAGGAAAGAACATGTGAGCAAAAGGCCAGCAAA
AGGCCAGGAACCGTAAAAAGGCCGCGTTGCTGGCGTTTTTCCATAGGCTC
CGCCCCCCTGACGAGCATCACAAAAATCGACGCTCAAGTCAGAGGTGGCG
AAACCCGACAGGACTATAAAGATACCAGGCGTTTCCCCCTGGAAGCTCCC
TCGTGCGCTCTCCTGTTCCGACCCTGCCGCTTACCGGATACCTGTCCGCC
TTTCTCCCTTCGGGAAGCGTGGCGCTTTCTCATAGCTCACGCTGTAGGTA
TCTCAGTTCGGTGTAGGTCGTTCGCTCCAAGCTGGGCTGTGTGCACGAAC
CCCCCGTTCAGCCCGACCGCTGCGCCTTATCCGGTAACTATCGTCTTGAG
TCCAACCCGGTAAGACACGACTTATCGCCACTGGCAGCAGCCACTGGTAA
CAGGATTAGCAGAGCGAGGTATGTAGGCGGTGCTACAGAGTTCTTGAAGT
GGTGGCCTAACTACGGCTACACTAGAAGGACAGTATTTGGTATCTGCGCT
CTGCTGAAGCCAGTTACCTTCGGAAAAAGAGTTGGTAGCTCTTGATCCGG
CAAACAAACCACCGCTGGTAGCGGTGGTTTTTTTGTTTGCAAGCAGCAGA
TTACGCGCAGAAAAAAAGGATCTCAAGAAGATCCTTTGATCTTTTCTACG
GGGTCTGACGCTCAGTGGAACGAAAACTCACGTTAAGGGATTTTGGTCAT
GAGATTATCAAAAAGGATCTTCACCTAGATCCTTTTAAATTAAAAATGAA
GTTTTAAATCAATCTAAAGTATATATGAGTAAACTTGGTCTGACAGTTAC
CAATGCTTAATCAGTGAGGCACCTATCTCAGCGATCTGTCTATTTCGTTC
ATCCATAGTTGCCTGACTCCCCGTCGTGTAGATAACTACGATACGGGAGG
GCTTACCATCTGGCCCCAGTGCTGCAATGATACCGCGAGACCCACGCTCA
CCGGCTCCAGATTTATCAGCAATAAACCAGCCAGCCGGAAGGGCCGAGCG
CAGAAGTGGTCCTGCAACTTTATCCGCCTCCATCCAGTCTATTAATTGTT
GCCGGGAAGCTAGAGTAAGTAGTTCGCCAGTTAATAGTTTGCGCAACGTT
GTTGCCATTGCTACAGGCATCGTGGTGTCACGCTCGTCGTTTGGTATGGC
TTCATTCAGCTCCGGTTCCCAACGATCAAGGCGAGTTACATGATCCCCCA
TGTTGTGCAAAAAAGCGGTTAGCTCCTTCGGTCCTCCGATCGTTGTCAGA
AGTAAGTTGGCCGCAGTGTTATCACTCATGGTTATGGCAGCACTGCATAA
TTCTCTTACTGTCATGCCATCCGTAAGATGCTTTTCTGTGACTGGTGAGT
ACTCAACCAAGTCATTCTGAGAATAGTGTATGCGGCGACCGAGTTGCTCT
TGCCCGGCGTCAATACGGGATAATACCGCGCCACATAGCAGAACTTTAAA

AGTGCTCATCATTGGAAAACGTTCTTCGGGGCGAAAACTCTCAAGGATCT
TACCGCTGTTGAGATCCAGTTCGATGTAACCCACTCGTGCACCCAACTGA
TCTTCAGCATCTTTTACTTTCACCAGCGTTTCTGGGTGAGCAAAAACAGG
AAGGCAAAATGCCGCAAAAAAGGGAATAAGGGCGACACGGAAATGTTGAA
TACTCATACTCTTCCTTTTTCAATATTATTGAAGCATTTATCAGGGTTAT
TGTCTCATGAGCGGATACATATTTGAATGTATTTAGAAAAATAAACAAAT
AGGGGTTCCGCGCACATTTCCCCGAAAAGTGCCACCTGACGTCTAAGAAA
CCATTATTATCATGACATTAACCTATAAAAATAGGCGTATCACGAGGCCC
TTTCGTC

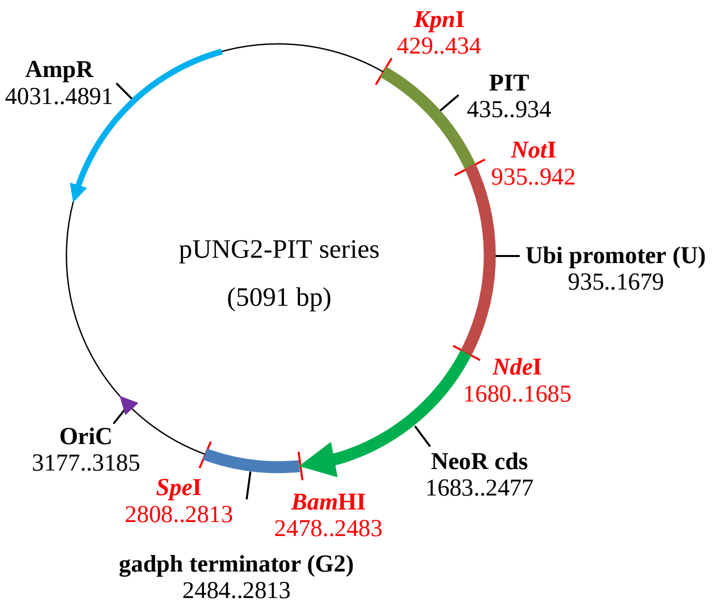

pUNG2-PIT24; 5091 bp

TCGCGCGTTTCGGTGATGACGGTGAAAACCTCTGACACATGCAGCTCCCG
GAGACGGTCACAGCTTGTCTGTAAGCGGATGCCGGGAGCAGACAAGCCCG
TCAGGGCGCGTCAGCGGGTGTTGGCGGGTGTCGGGGCTGGCTTAACTATG
CGGCATCAGAGCAGATTGTACTGAGAGTGCACCATATGCGGTGTGAAATA
CCGCACAGATGCGTAAGGAGAAAATACCGCATCAGGCGCCATTCGCCATT
CAGGCTGCGCAACTGTTGGGAAGGGCGATCGGTGCGGGCCTCTTCGCTAT
TACGCCAGCTGGCGAAAGGGGGATGTGCTGCAAGGCGATTAAGTTGGGTA
ACGCCAGGGTTTTCCCAGTCACGACGTTGTAAAACGACGGCCAGTGAATT
GTAATACGACTCACTATAGGGCGAGCTCGGTACCGAGATAAGAAGCCTCG
AGTAAACTATTTTCAGGCTTATTTTTGAAAACTCGCAACCCTAGTATATT
ACTAAGGAAATTTTTCTGCTTTCAATCGGAACAGATATCACATTTGTTTG
AAATTGTCTCTTTTTTTGTTTTCTGCAACAAATATGTAACGATTGTTTGTT
AGTAAGGAACGAATTAATGTCCTTAATTTTTCTAAAACCTTATCGACATT
ATTTGATTTATGTTTAGAATACGACATCGTCTCTGAACAACTTTTCATAA
TAAAATTTACCTGCCTTTTCTCTAGACGAGGTGAGAAAAATGAATATTCA
ATAGATAATCCTTATTGTTAAAGAATTGAAATTGAAAAGGTCCAAGCTAA
CAAAAAGGATGTGATCTGATAGCATGGATTTTTTCATCGAACACTGGAAA
ATTGGTTTTGAACGTTTATTGACTAATAGCATTTTAATAATGTTGACAGT
AAATTGATTCACATATTTTTTCTTATCTTAGACGGCGGCCGCGGGAGACT
TTGAAAGGCACAGTAAGTTTTTGAAAGATAAAAGGGATAAAGATCAAAAA
GGTCATCCTGGCAGACATGTGGAAATCCAAGAGCACTGAAAACGCGAAGG

TTCTCAGAGGTCTCTGCTCCATGTTCATGGCAGTCCGAACGGCTTTGCCT
AAGACTCGGTACTATGCTACAGCTCCAGAAGTCAGTCAGTCAGTCTGTCT
GAGCAAAATGAGCGAATTTTTAAATCCTTTGCGAAGCTTTTTCGTTTGAA
AGTTGTTTCTTTAAAATGGAAAGTTGATTGATTGACCCCGATCACTGAAG
CTTTATCGCAAGAGGCAGAGAGAACAGACGGTGCGAGATGGGTATTATTG
TAAACGCTATGCGCGCAATAGCATCCAATCAAGAGCGCAATCGAGCAACA
GACGGAGATCAAAAAATAAGATAATAAAAATAAAACTGACTATATGTAGG
AGTGGTTATGGATTTTGCTAAGATACTGGCGATTGTGTTGAATTGAAAAG
AAAGAAATAGAAGAAGAAGAAGAAGAGAGAACAATGCTTTTATTTTACAA
AGCAGCAGCTTACTTTAAAGTGCTTTTCATGGACTGAGCAGAATCACTCG
AGGCGCAGCAAAAACCCAACTCCAGCCAGGCCAGTCCAGACCGAAGAAAG
CAAGCGTAGGGAGCTCAGCTTGATCCTGGTGCTGTTCATTGAGATTGACA
AAGTTGTAAGAATTAGATAAGCAAACAACCATATGATTGAACAAGATGGA
TTGCACGCAGGTTCTCCGGCCGCTTGGGTGGAGAGGCTATTCGGCTATGA
CTGGGCACAACAGACAATCGGCTGCTCTGATGCCGCCGTGTTCCGGCTGT
CAGCGCAGGGGCGCCCGGTTCTTTTTGTCAAGACCGACCTGTCCGGTGCC
CTGAATGAACTGCAAGACGAGGCAGCGCGGCTATCGTGGCTGGCCACGAC
GGGCGTTCCCTGCGCAGCTGTGCTCGACGTTGTCACTGAAGCGGGAAGGG
ACTGGCTGCTATTGGGCGAAGTGCCGGGGCAGGATCTCCTGTCATCTCAC
CTTGCTCCTGCCGAGAAAGTATCCATCATGGCTGATGCAATGCGGCGGCT
GCATACGCTTGATCCGGCTACCTGCCCATTCGACCACCAAGCGAAACATC
GCATCGAGCGAGCACGTACTCGGATGGAAGCCGGTCTTGTCGATCAGGAT
GATCTGGACGAAGAGCATCAGGGGCTCGCGCCAGCCGAACTGTTCGCCAG
GCTCAAGGCGAGCATGCCCGACGGCGAGGATCTCGTCGTGACCCATGGCG
ATGCCTGCTTGCCGAATATCATGGTGGAAAATGGCCGCTTTTCTGGATTC
ATCGACTGTGGCCGGCTGGGTGTGGCGGACCGCTATCAGGACATAGCGTT
GGCTACCCGTGATATTGCTGAAGAGCTTGGCGGCGAATGGGCTGACCGCT
TCCTCGTGCTTTACGGTATCGCCGCTCCCGATTCGCAGCGCATCGCCTTC
TATCGCCTTCTTGACGAGTTCTTCTAAGGATCCATGTACCCAATACCACA
CCGGTAGCTTCTCGCGGCGGCTGACAAGAAAGATTGTTTTTACACATTTC
GAGGCATTAATGACCCTTATCGACCTATCGTCTCAGATCATAAAATGCAC
GAGCATGAAGTACGCGTGTTGTGACTTGCTTGCGTCCCACCTTCTAGATG
GCTTTCTTCTTTTCAAGTGATTAAAACACCACAGTAGATGAGGACTCTTA
GTAAGCACTGAAGAAGCGAGTAGATAGCCCTCATCCCGTTTCCCTCTTTT
CTAACACACTTTGTTTGGAATTCTAAAATATCTTTATCATCTCTTTTCAT
TCACAAAACTAGTAGATCTGGATCCCCTCTAGAGTCGACCTGCAGGCATG
CAAGCTTGGCGTAATCATGGTCATAGCTGTTTCCTGTGTGAAATTGTTAT
CCGCTCACAATTCCACACAACATACGAGCCGGAAGCATAAAGTGTAAAGC

CTGGGGTGCCTAATGAGTGAGCTAACTCACATTAATTGCGTTGCGCTCAC
TGCCCGCTTTCCAGTCGGGAAACCTGTCGTGCCAGCTGCATTAATGAATC
GGCCAACGCGCGGGGAGAGGCGGTTTGCGTATTGGGCGCTCTTCCGCTTC
CTCGCTCACTGACTCGCTGCGCTCGGTCGTTCGGCTGCGGCGAGCGGTAT
CAGCTCACTCAAAGGCGGTAATACGGTTATCCACAGAATCAGGGGATAAC
GCAGGAAAGAACATGTGAGCAAAAGGCCAGCAAAAGGCCAGGAACCGTAA
AAAGGCCGCGTTGCTGGCGTTTTTCCATAGGCTCCGCCCCCCTGACGAGC
ATCACAAAAATCGACGCTCAAGTCAGAGGTGGCGAAACCCGACAGGACTA
TAAAGATACCAGGCGTTTCCCCCTGGAAGCTCCCTCGTGCGCTCTCCTGT
TCCGACCCTGCCGCTTACCGGATACCTGTCCGCCTTTCTCCCTTCGGGAA
GCGTGGCGCTTTCTCATAGCTCACGCTGTAGGTATCTCAGTTCGGTGTAG
GTCGTTCGCTCCAAGCTGGGCTGTGTGCACGAACCCCCCGTTCAGCCCGA
CCGCTGCGCCTTATCCGGTAACTATCGTCTTGAGTCCAACCCGGTAAGAC
ACGACTTATCGCCACTGGCAGCAGCCACTGGTAACAGGATTAGCAGAGCG
AGGTATGTAGGCGGTGCTACAGAGTTCTTGAAGTGGTGGCCTAACTACGG
CTACACTAGAAGGACAGTATTTGGTATCTGCGCTCTGCTGAAGCCAGTTA
CCTTCGGAAAAAGAGTTGGTAGCTCTTGATCCGGCAAACAAACCACCGCT
GGTAGCGGTGGTTTTTTTGTTTGCAAGCAGCAGATTACGCGCAGAAAAAA
AGGATCTCAAGAAGATCCTTTGATCTTTTCTACGGGGTCTGACGCTCAGT
GGAACGAAAACTCACGTTAAGGGATTTTGGTCATGAGATTATCAAAAAGG
ATCTTCACCTAGATCCTTTTAAATTAAAAATGAAGTTTTAAATCAATCTA
AAGTATATATGAGTAAACTTGGTCTGACAGTTACCAATGCTTAATCAGTG
AGGCACCTATCTCAGCGATCTGTCTATTTCGTTCATCCATAGTTGCCTGA
CTCCCCGTCGTGTAGATAACTACGATACGGGAGGGCTTACCATCTGGCCC
CAGTGCTGCAATGATACCGCGAGACCCACGCTCACCGGCTCCAGATTTAT
CAGCAATAAACCAGCCAGCCGGAAGGGCCGAGCGCAGAAGTGGTCCTGCA
ACTTTATCCGCCTCCATCCAGTCTATTAATTGTTGCCGGGAAGCTAGAGT
AAGTAGTTCGCCAGTTAATAGTTTGCGCAACGTTGTTGCCATTGCTACAG
GCATCGTGGTGTCACGCTCGTCGTTTGGTATGGCTTCATTCAGCTCCGGT
TCCCAACGATCAAGGCGAGTTACATGATCCCCCATGTTGTGCAAAAAAGC
GGTTAGCTCCTTCGGTCCTCCGATCGTTGTCAGAAGTAAGTTGGCCGCAG
TGTTATCACTCATGGTTATGGCAGCACTGCATAATTCTCTTACTGTCATG
CCATCCGTAAGATGCTTTTCTGTGACTGGTGAGTACTCAACCAAGTCATT
CTGAGAATAGTGTATGCGGCGACCGAGTTGCTCTTGCCCGGCGTCAATAC
GGGATAATACCGCGCCACATAGCAGAACTTTAAAAGTGCTCATCATTGGA
AAACGTTCTTCGGGGCGAAAACTCTCAAGGATCTTACCGCTGTTGAGATC
CAGTTCGATGTAACCCACTCGTGCACCCAACTGATCTTCAGCATCTTTTA
CTTTCACCAGCGTTTCTGGGTGAGCAAAAACAGGAAGGCAAAATGCCGCA

AAAAAGGGAATAAGGGCGACACGGAAATGTTGAATACTCATACTCTTCCT
TTTTCAATATTATTGAAGCATTTATCAGGGTTATTGTCTCATGAGCGGAT
ACATATTTGAATGTATTTAGAAAAATAAACAAATAGGGGTTCCGCGCACA
TTTCCCCGAAAAGTGCCACCTGACGTCTAAGAAACCATTATTATCATGAC
ATTAACCTATAAAAATAGGCGTATCACGAGGCCCTTTCGTC

pUNG2-PIT29; 5091 bp

TCGCGCGTTTCGGTGATGACGGTGAAAACCTCTGACACATGCAGCTCCCG
GAGACGGTCACAGCTTGTCTGTAAGCGGATGCCGGGAGCAGACAAGCCCG
TCAGGGCGCGTCAGCGGGTGTTGGCGGGTGTCGGGGCTGGCTTAACTATG
CGGCATCAGAGCAGATTGTACTGAGAGTGCACCATATGCGGTGTGAAATA
CCGCACAGATGCGTAAGGAGAAAATACCGCATCAGGCGCCATTCGCCATT
CAGGCTGCGCAACTGTTGGGAAGGGCGATCGGTGCGGGCCTCTTCGCTAT
TACGCCAGCTGGCGAAAGGGGGATGTGCTGCAAGGCGATTAAGTTGGGTA
ACGCCAGGGTTTTCCCAGTCACGACGTTGTAAAACGACGGCCAGTGAATT
GTAATACGACTCACTATAGGGCGAGCTCGGTACCAAATTTTTCTGCTTTC
AATCGGAACAGATATCACATTTGTTTGAAATTGTCTCTTTTTTGTTTTCT
GCAACAAATGTGTAACGATTGTTTGTTAGTAAGGAACGAATTAATGTCCT
TAATTTTTCTAAAACCTTATCGACATTATTTGATTTATGTTTAGAATATG
ACATCGTCTCTGAACAACTTTTCTTAAAAAAATTTACCTGCCTTTTCTCT
AGACGAGGTGAGAAAAATGAATATTCAATAGATAATCCTTATTGTTAAAG
AATTGAAATTGAAAAGGTCCAAGCTAACAAAAAGGATGTGATCTGATAGC
ATGGATTTTTTCATCGAACACTGGAAAATTGGTTTTGAACGTTTATTGAC
TAATAGCATTTTAATAATGTTGACAGTCAATTGATTCACATATTTTTTCT
TATCTTAAACGGTAATTTTGGATCAACAAATTTTATGATGATGTTTATGG
CTTTTTTGTCCGATGTATTTTGAATACAACTCTGGCGGCCGCGGGAGACT
TTGAAAGGCACAGTAAGTTTTTGAAAGATAAAAGGGATAAAGATCAAAAA
GGTCATCCTGGCAGACATGTGGAAATCCAAGAGCACTGAAAACGCGAAGG
TTCTCAGAGGTCTCTGCTCCATGTTCATGGCAGTCCGAACGGCTTTGCCT
AAGACTCGGTACTATGCTACAGCTCCAGAAGTCAGTCAGTCAGTCTGTCT
GAGCAAAATGAGCGAATTTTTAAATCCTTTGCGAAGCTTTTTCGTTTGAA
AGTTGTTTCTTTAAAATGGAAAGTTGATTGATTGACCCCGATCACTGAAG
CTTTATCGCAAGAGGCAGAGAGAACAGACGGTGCGAGATGGGTATTATTG
TAAACGCTATGCGCGCAATAGCATCCAATCAAGAGCGCAATCGAGCAACA
GACGGAGATCAAAAAATAAGATAATAAAAATAAAACTGACTATATGTAGG
AGTGGTTATGGATTTTGCTAAGATACTGGCGATTGTGTTGAATTGAAAAG
AAAGAAATAGAAGAAGAAGAAGAAGAGAGAACAATGCTTTTATTTTACAA
AGCAGCAGCTTACTTTAAAGTGCTTTTCATGGACTGAGCAGAATCACTCG
AGGCGCAGCAAAAACCCAACTCCAGCCAGGCCAGTCCAGACCGAAGAAAG
CAAGCGTAGGGAGCTCAGCTTGATCCTGGTGCTGTTCATTGAGATTGACA
AAGTTGTAAGAATTAGATAAGCAAACAACCATATGATTGAACAAGATGGA
TTGCACGCAGGTTCTCCGGCCGCTTGGGTGGAGAGGCTATTCGGCTATGA
CTGGGCACAACAGACAATCGGCTGCTCTGATGCCGCCGTGTTCCGGCTGT
CAGCGCAGGGGCGCCCGGTTCTTTTGTCAAGACCGACCTGTCCGGTGCC

CTGAATGAACTGCAAGACGAGGCAGCGCGGCTATCGTGGCTGGCCACGAC
GGGCGTTCCCTGCGCAGCTGTGCTCGACGTTGTCACTGAAGCGGGAAGGG
ACTGGCTGCTATTGGGCGAAGTGCCGGGGCAGGATCTCCTGTCATCTCAC
CTTGCTCCTGCCGAGAAAGTATCCATCATGGCTGATGCAATGCGGCGGCT
GCATACGCTTGATCCGGCTACCTGCCCATTCGACCACCAAGCGAAACATC
GCATCGAGCGAGCACGTACTCGGATGGAAGCCGGTCTTGTCGATCAGGAT
GATCTGGACGAAGAGCATCAGGGGCTCGCGCCAGCCGAACTGTTCGCCAG
GCTCAAGGCGAGCATGCCCGACGGCGAGGATCTCGTCGTGACCCATGGCG
ATGCCTGCTTGCCGAATATCATGGTGGAAAATGGCCGCTTTTCTGGATTC
ATCGACTGTGGCCGGCTGGGTGTGGCGGACCGCTATCAGGACATAGCGTT
GGCTACCCGTGATATTGCTGAAGAGCTTGGCGGCGAATGGGCTGACCGCT
TCCTCGTGCTTTACGGTATCGCCGCTCCCGATTCGCAGCGCATCGCCTTC
TATCGCCTTCTTGACGAGTTCTTCTAAGGATCCATGTACCCAATACCACA
CCGGTAGCTTCTCGCGGCGGCTGACAAGAAAGATTGTTTTTACACATTTC
GAGGCATTAATGACCCTTATCGACCTATCGTCTCAGATCATAAAATGCAC
GAGCATGAAGTACGCGTGTTGTGACTTGCTTGCGTCCCACCTTCTAGATG
GCTTTCTTCTTTTCAAGTGATTAAAACACCACAGTAGATGAGGACTCTTA
GTAAGCACTGAAGAAGCGAGTAGATAGCCCTCATCCCGTTTCCCTCTTTT
CTAACACACTTTGTTTGGAATTCTAAAATATCTTTATCATCTCTTTTCAT
TCACAAAACTAGTAGATCTGGATCCCCTCTAGAGTCGACCTGCAGGCATG
CAAGCTTGGCGTAATCATGGTCATAGCTGTTTCCTGTGTGAAATTGTTAT
CCGCTCACAATTCCACACAACATACGAGCCGGAAGCATAAAGTGTAAAGC
CTGGGGTGCCTAATGAGTGAGCTAACTCACATTAATTGCGTTGCGCTCAC
TGCCCGCTTTCCAGTCGGGAAACCTGTCGTGCCAGCTGCATTAATGAATC
GGCCAACGCGCGGGGAGAGGCGGTTTGCGTATTGGGCGCTCTTCCGCTTC
CTCGCTCACTGACTCGCTGCGCTCGGTCGTTCGGCTGCGGCGAGCGGTAT
CAGCTCACTCAAAGGCGGTAATACGGTTATCCACAGAATCAGGGGATAAC
GCAGGAAAGAACATGTGAGCAAAAGGCCAGCAAAAGGCCAGGAACCGTAA
AAAGGCCGCGTTGCTGGCGTTTTTCCATAGGCTCCGCCCCCCTGACGAGC
ATCACAAAAATCGACGCTCAAGTCAGAGGTGGCGAAACCCGACAGGACTA
TAAAGATACCAGGCGTTTCCCCCTGGAAGCTCCCTCGTGCGCTCTCCTGT
TCCGACCCTGCCGCTTACCGGATACCTGTCCGCCTTTCTCCCTTCGGGAA
GCGTGGCGCTTTCTCATAGCTCACGCTGTAGGTATCTCAGTTCGGTGTAG
GTCGTTCGCTCCAAGCTGGGCTGTGTGCACGAACCCCCCGTTCAGCCCGA
CCGCTGCGCCTTATCCGGTAACTATCGTCTTGAGTCCAACCCGGTAAGAC
ACGACTTATCGCCACTGGCAGCAGCCACTGGTAACAGGATTAGCAGAGCG
AGGTATGTAGGCGGTGCTACAGAGTTCTTGAAGTGGTGGCCTAACTACGG
CTACACTAGAAGGACAGTATTTGGTATCTGCGCTCTGCTGAAGCCAGTTA

CCTTCGGAAAAAGAGTTGGTAGCTCTTGATCCGGCAAACAAACCACCGCT
GGTAGCGGTGGTTTTTTTGTTTGCAAGCAGCAGATTACGCGCAGAAAAAA
AGGATCTCAAGAAGATCCTTTGATCTTTTCTACGGGGTCTGACGCTCAGT
GGAACGAAAACTCACGTTAAGGGATTTTGGTCATGAGATTATCAAAAAGG
ATCTTCACCTAGATCCTTTTAAATTAAAAATGAAGTTTTAAATCAATCTA
AAGTATATATGAGTAAACTTGGTCTGACAGTTACCAATGCTTAATCAGTG
AGGCACCTATCTCAGCGATCTGTCTATTTCGTTCATCCATAGTTGCCTGA
CTCCCCGTCGTGTAGATAACTACGATACGGGAGGGCTTACCATCTGGCCC
CAGTGCTGCAATGATACCGCGAGACCCACGCTCACCGGCTCCAGATTTAT
CAGCAATAAACCAGCCAGCCGGAAGGGCCGAGCGCAGAAGTGGTCCTGCA
ACTTTATCCGCCTCCATCCAGTCTATTAATTGTTGCCGGGAAGCTAGAGT
AAGTAGTTCGCCAGTTAATAGTTTGCGCAACGTTGTTGCCATTGCTACAG
GCATCGTGGTGTCACGCTCGTCGTTTGGTATGGCTTCATTCAGCTCCGGT
TCCCAACGATCAAGGCGAGTTACATGATCCCCCATGTTGTGCAAAAAAGC
GGTTAGCTCCTTCGGTCCTCCGATCGTTGTCAGAAGTAAGTTGGCCGCAG
TGTTATCACTCATGGTTATGGCAGCACTGCATAATTCTCTTACTGTCATG
CCATCCGTAAGATGCTTTTCTGTGACTGGTGAGTACTCAACCAAGTCATT
CTGAGAATAGTGTATGCGGCGACCGAGTTGCTCTTGCCCGGCGTCAATAC
GGGATAATACCGCGCCACATAGCAGAACTTTAAAAGTGCTCATCATTGGA
AAACGTTCTTCGGGGCGAAAACTCTCAAGGATCTTACCGCTGTTGAGATC
CAGTTCGATGTAACCCACTCGTGCACCCAACTGATCTTCAGCATCTTTTA
CTTTCACCAGCGTTTCTGGGTGAGCAAAAACAGGAAGGCAAAATGCCGCA
AAAAAGGGAATAAGGGCGACACGGAAATGTTGAATACTCATACTCTTCCT
TTTTCAATATTATTGAAGCATTTATCAGGGTTATTGTCTCATGAGCGGAT
ACATATTTGAATGTATTTAGAAAAATAAACAAATAGGGGTTCCGCGCACA
TTTCCCCGAAAAGTGCCACCTGACGTCTAAGAAACCATTATTATCATGAC
ATTAACCTATAAAAATAGGCGTATCACGAGGCCCTTTCGTC

pUNG2-PIT30; 5091 bp

TCGCGCGTTTCGGTGATGACGGTGAAAACCTCTGACACATGCAGCTCCCG
GAGACGGTCACAGCTTGTCTGTAAGCGGATGCCGGGAGCAGACAAGCCCG
TCAGGGCGCGTCAGCGGGTGTTGGCGGGTGTCGGGGCTGGCTTAACTATG
CGGCATCAGAGCAGATTGTACTGAGAGTGCACCATATGCGGTGTGAAATA
CCGCACAGATGCGTAAGGAGAAAATACCGCATCAGGCGCCATTCGCCATT
CAGGCTGCGCAACTGTTGGGAAGGGCGATCGGTGCGGGCCTCTTCGCTAT
TACGCCAGCTGGCGAAAGGGGGATGTGCTGCAAGGCGATTAAGTTGGGTA
ACGCCAGGGTTTTCCCAGTCACGACGTTGTAAAACGACGGCCAGTGAATT
GTAATACGACTCACTATAGGGCGAGCTCGGTACCGGATTCAAACTTGTGC
CATTCATTTTTGAAAAGACTGAACTTTCCTTTTCTTCTCGGTTTCTTATC
TCCATATTTGATTCAAATAAGACTTGTTTGACAATTTCCAGTTTTTTAAA
ATTAAAAAAAGATAAAAAGAGCGACTAGAGTTGCTGTTTAAATCTAAAAA
TTAAATAATTGCATAACCTGCTTAAAAATCTTCGAAAATTAGTCTGGTTA
TCAGTGCCATGAAATACAGTGAAGATTGTTATCCCTGTAAGGAACTGAAA
TTGCCTTTCCAAGTAAGTAACTTTCCATTTTTTCAGTTTATGCATGGTAT
TTTACAATTGAAGCTCCCGGATAACTTTGGTATAACATATTAAATTTCTT
ATGTTAGTTTTTTTCAAGTCTTTCGTTACTACTGACATGAAAATTTTTTT
GGTTCATTACCCTACATTTCCTGATTTCGATCCACAAAACGAGAACAAAC
GAATTCCCGTAAAATTTCAAATATGCCGCAAACAGCGGCCGCGGGAGACT
TTGAAAGGCACAGTAAGTTTTTGAAAGATAAAAGGGATAAAGATCAAAAA
GGTCATCCTGGCAGACATGTGGAAATCCAAGAGCACTGAAAACGCGAAGG
TTCTCAGAGGTCTCTGCTCCATGTTCATGGCAGTCCGAACGGCTTTGCCT
AAGACTCGGTACTATGCTACAGCTCCAGAAGTCAGTCAGTCAGTCTGTCT
GAGCAAAATGAGCGAATTTTTAAATCCTTTGCGAAGCTTTTTCGTTTGAA
AGTTGTTTCTTTAAAATGGAAAGTTGATTGATTGACCCCGATCACTGAAG
CTTTATCGCAAGAGGCAGAGAGAACAGACGGTGCGAGATGGGTATTATTG
TAAACGCTATGCGCGCAATAGCATCCAATCAAGAGCGCAATCGAGCAACA
GACGGAGATCAAAAAATAAGATAATAAAAATAAAACTGACTATATGTAGG
AGTGGTTATGGATTTTGCTAAGATACTGGCGATTGTGTTGAATTGAAAAG
AAAGAAATAGAAGAAGAAGAAGAAGAGAGAACAATGCTTTTATTTTACAA
AGCAGCAGCTTACTTTAAAGTGCTTTTCATGGACTGAGCAGAATCACTCG
AGGCGCAGCAAAAACCCAACTCCAGCCAGGCCAGTCCAGACCGAAGAAAG
CAAGCGTAGGGAGCTCAGCTTGATCCTGGTGCTGTTCATTGAGATTGACA
AAGTTGTAAGAATTAGATAAGCAAACAACCATATGATTGAACAAGATGGA
TTGCACGCAGGTTCTCCGGCCGCTTGGGTGGAGAGGCTATTCGGCTATGA
CTGGGCACAACAGACAATCGGCTGCTCTGATGCCGCCGTGTTCCGGCTGT
CAGCGCAGGGGCGCCCGGTTCTTTTTGTCAAGACCGACCTGTCCGGTGCC

CTGAATGAACTGCAAGACGAGGCAGCGCGGCTATCGTGGCTGGCCACGAC
GGGCGTTCCCTGCGCAGCTGTGCTCGACGTTGTCACTGAAGCGGGAAGGG
ACTGGCTGCTATTGGGCGAAGTGCCGGGGCAGGATCTCCTGTCATCTCAC
CTTGCTCCTGCCGAGAAAGTATCCATCATGGCTGATGCAATGCGGCGGCT
GCATACGCTTGATCCGGCTACCTGCCCATTCGACCACCAAGCGAAACATC
GCATCGAGCGAGCACGTACTCGGATGGAAGCCGGTCTTGTCGATCAGGAT
GATCTGGACGAAGAGCATCAGGGGCTCGCGCCAGCCGAACTGTTCGCCAG
GCTCAAGGCGAGCATGCCCGACGGCGAGGATCTCGTCGTGACCCATGGCG
ATGCCTGCTTGCCGAATATCATGGTGGAAAATGGCCGCTTTTCTGGATTC
ATCGACTGTGGCCGGCTGGGTGTGGCGGACCGCTATCAGGACATAGCGTT
GGCTACCCGTGATATTGCTGAAGAGCTTGGCGGCGAATGGGCTGACCGCT
TCCTCGTGCTTTACGGTATCGCCGCTCCCGATTCGCAGCGCATCGCCTTC
TATCGCCTTCTTGACGAGTTCTTCTAAGGATCCATGTACCCAATACCACA
CCGGTAGCTTCTCGCGGCGGCTGACAAGAAAGATTGTTTTTACACATTTC
GAGGCATTAATGACCCTTATCGACCTATCGTCTCAGATCATAAAATGCAC
GAGCATGAAGTACGCGTGTTGTGACTTGCTTGCGTCCCACCTTCTAGATG
GCTTTCTTCTTTTCAAGTGATTAAAACACCACAGTAGATGAGGACTCTTA
GTAAGCACTGAAGAAGCGAGTAGATAGCCCTCATCCCGTTTCCCTCTTTT
CTAACACACTTTGTTTGGAATTCTAAAATATCTTTATCATCTCTTTTCAT
TCACAAAACTAGTAGATCTGGATCCCCTCTAGAGTCGACCTGCAGGCATG
CAAGCTTGGCGTAATCATGGTCATAGCTGTTTCCTGTGTGAAATTGTTAT
CCGCTCACAATTCCACACAACATACGAGCCGGAAGCATAAAGTGTAAAGC
CTGGGGTGCCTAATGAGTGAGCTAACTCACATTAATTGCGTTGCGCTCAC
TGCCCGCTTTCCAGTCGGGAAACCTGTCGTGCCAGCTGCATTAATGAATC
GGCCAACGCGCGGGGAGAGGCGGTTTGCGTATTGGGCGCTCTTCCGCTTC
CTCGCTCACTGACTCGCTGCGCTCGGTCGTTCGGCTGCGGCGAGCGGTAT
CAGCTCACTCAAAGGCGGTAATACGGTTATCCACAGAATCAGGGGATAAC
GCAGGAAAGAACATGTGAGCAAAAGGCCAGCAAAAGGCCAGGAACCGTAA
AAAGGCCGCGTTGCTGGCGTTTTTCCATAGGCTCCGCCCCCCTGACGAGC
ATCACAAAAATCGACGCTCAAGTCAGAGGTGGCGAAACCCGACAGGACTA
TAAAGATACCAGGCGTTTCCCCCTGGAAGCTCCCTCGTGCGCTCTCCTGT
TCCGACCCTGCCGCTTACCGGATACCTGTCCGCCTTTCTCCCTTCGGGAA
GCGTGGCGCTTTCTCATAGCTCACGCTGTAGGTATCTCAGTTCGGTGTAG
GTCGTTCGCTCCAAGCTGGGCTGTGTGCACGAACCCCCCGTTCAGCCCGA
CCGCTGCGCCTTATCCGGTAACTATCGTCTTGAGTCCAACCCGGTAAGAC
ACGACTTATCGCCACTGGCAGCAGCCACTGGTAACAGGATTAGCAGAGCG
AGGTATGTAGGCGGTGCTACAGAGTTCTTGAAGTGGTGGCCTAACTACGG
CTACACTAGAAGGACAGTATTTGGTATCTGCGCTCTGCTGAAGCCAGTTA

CCTTCGGAAAAAGAGTTGGTAGCTCTTGATCCGGCAAACAAACCACCGCT
GGTAGCGGTGGTTTTTTTGTTTGCAAGCAGCAGATTACGCGCAGAAAAAA
AGGATCTCAAGAAGATCCTTTGATCTTTTCTACGGGGTCTGACGCTCAGT
GGAACGAAAACTCACGTTAAGGGATTTTGGTCATGAGATTATCAAAAAGG
ATCTTCACCTAGATCCTTTTAAATTAAAAATGAAGTTTTAAATCAATCTA
AAGTATATATGAGTAAACTTGGTCTGACAGTTACCAATGCTTAATCAGTG
AGGCACCTATCTCAGCGATCTGTCTATTTCGTTCATCCATAGTTGCCTGA
CTCCCCGTCGTGTAGATAACTACGATACGGGAGGGCTTACCATCTGGCCC
CAGTGCTGCAATGATACCGCGAGACCCACGCTCACCGGCTCCAGATTTAT
CAGCAATAAACCAGCCAGCCGGAAGGGCCGAGCGCAGAAGTGGTCCTGCA
ACTTTATCCGCCTCCATCCAGTCTATTAATTGTTGCCGGGAAGCTAGAGT
AAGTAGTTCGCCAGTTAATAGTTTGCGCAACGTTGTTGCCATTGCTACAG
GCATCGTGGTGTCACGCTCGTCGTTTGGTATGGCTTCATTCAGCTCCGGT
TCCCAACGATCAAGGCGAGTTACATGATCCCCCATGTTGTGCAAAAAAGC
GGTTAGCTCCTTCGGTCCTCCGATCGTTGTCAGAAGTAAGTTGGCCGCAG
TGTTATCACTCATGGTTATGGCAGCACTGCATAATTCTCTTACTGTCATG
CCATCCGTAAGATGCTTTTCTGTGACTGGTGAGTACTCAACCAAGTCATT
CTGAGAATAGTGTATGCGGCGACCGAGTTGCTCTTGCCCGGCGTCAATAC
GGGATAATACCGCGCCACATAGCAGAACTTTAAAAGTGCTCATCATTGGA
AAACGTTCTTCGGGGCGAAAACTCTCAAGGATCTTACCGCTGTTGAGATC
CAGTTCGATGTAACCCACTCGTGCACCCAACTGATCTTCAGCATCTTTTA
CTTTCACCAGCGTTTCTGGGTGAGCAAAAACAGGAAGGCAAAATGCCGCA
AAAAAGGGAATAAGGGCGACACGGAAATGTTGAATACTCATACTCTTCCT
TTTTCAATATTATTGAAGCATTTATCAGGGTTATTGTCTCATGAGCGGAT
ACATATTTGAATGTATTTAGAAAAATAAACAAATAGGGGTTCCGCGCACA
TTTCCCCGAAAAGTGCCACCTGACGTCTAAGAAACCATTATTATCATGAC
ATTAACCTATAAAAATAGGCGTATCACGAGGCCCTTTCGTC

Supplemental Fig. S1 Maps and sequences of circular vectors utilized in this study.

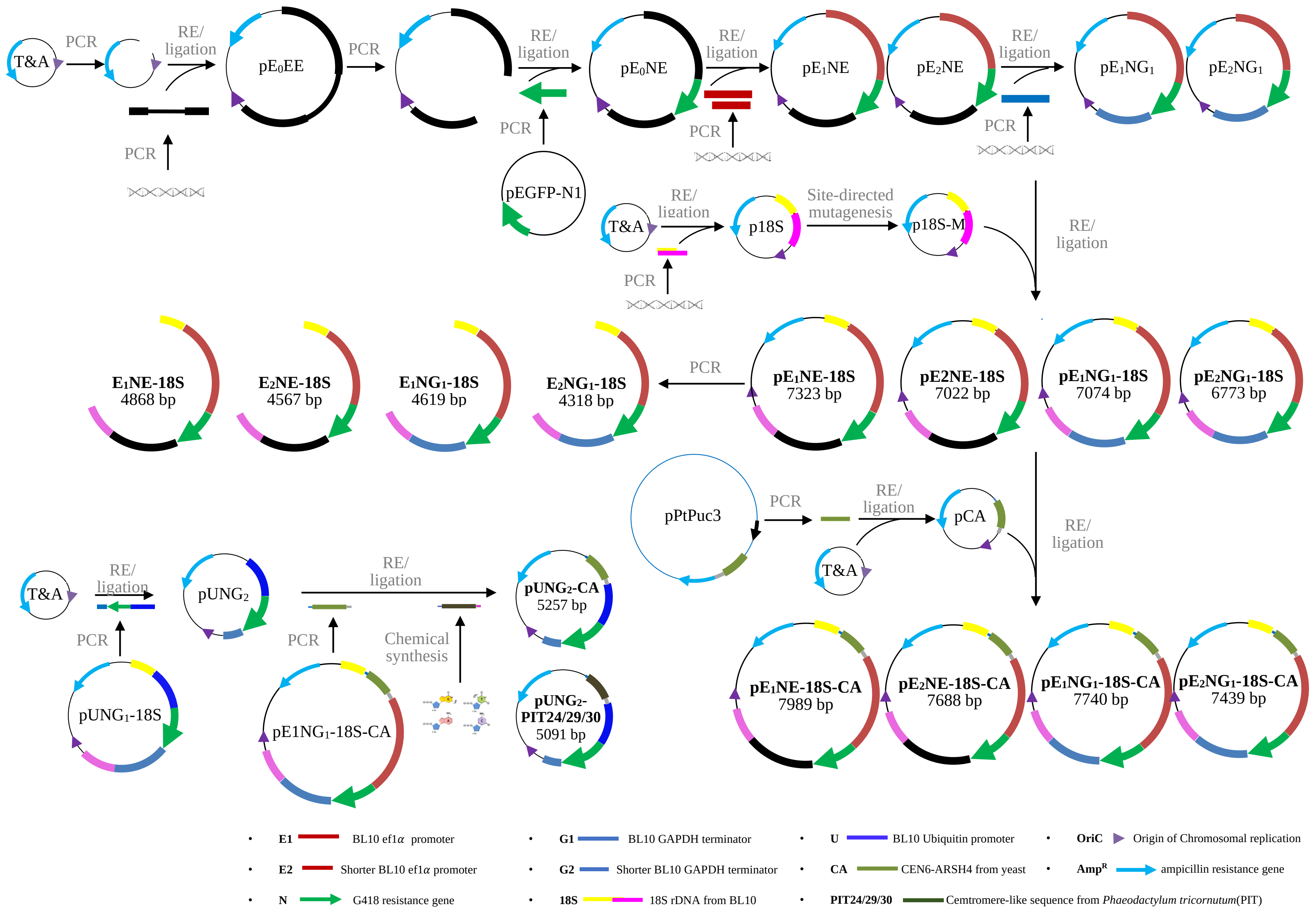

Supplemental Fig. S2 Flow chart illustrating plasmid construction process. See Materials and Methods for details.

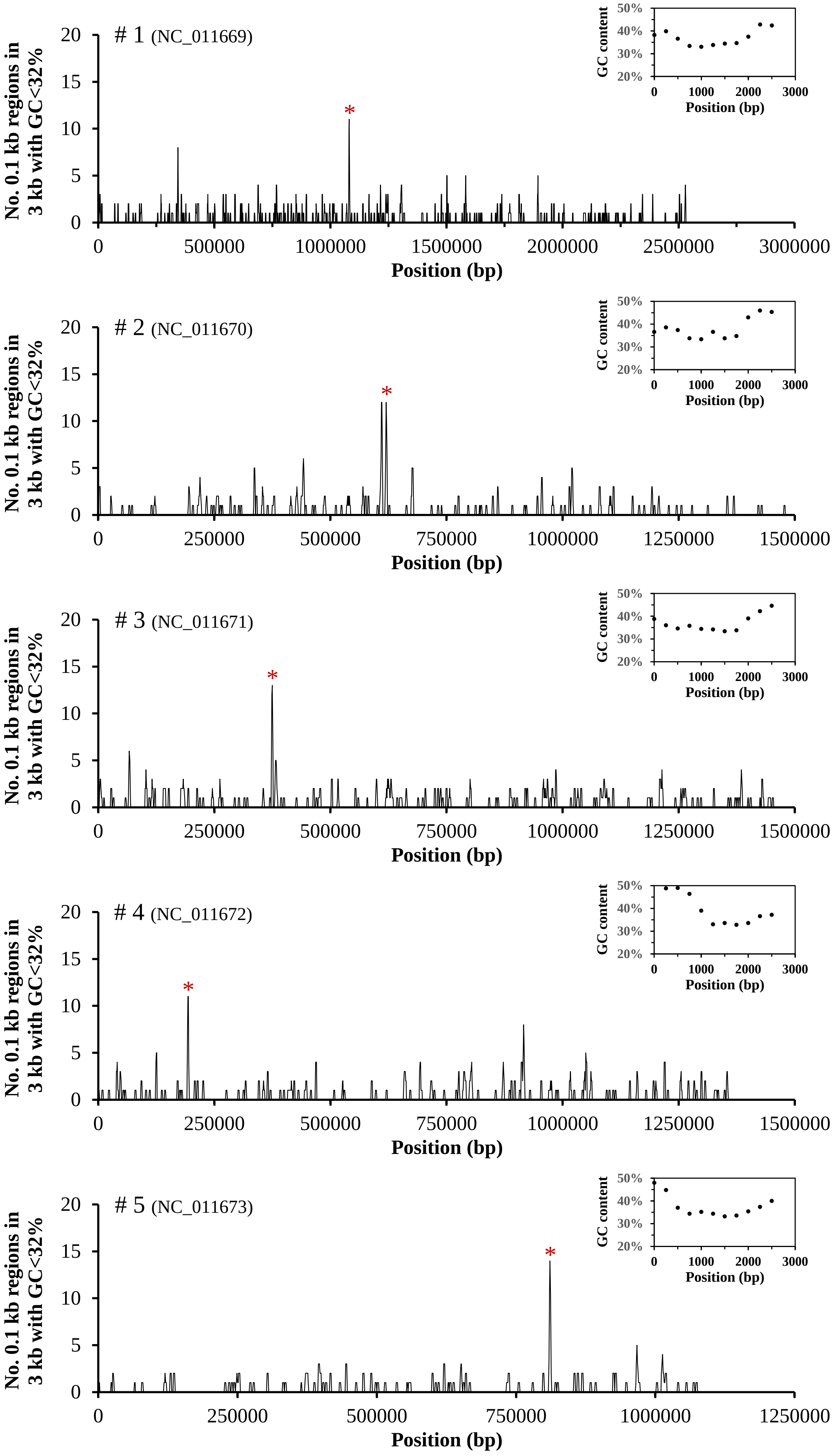
No. 0.1 kb regions in 3 kb with GC<32%
1 (NC_011669)
Position (bp)
GC content
2 (NC_011670)
3 (NC_011671)
4 (NC_011672)
5 (NC_011673)
*

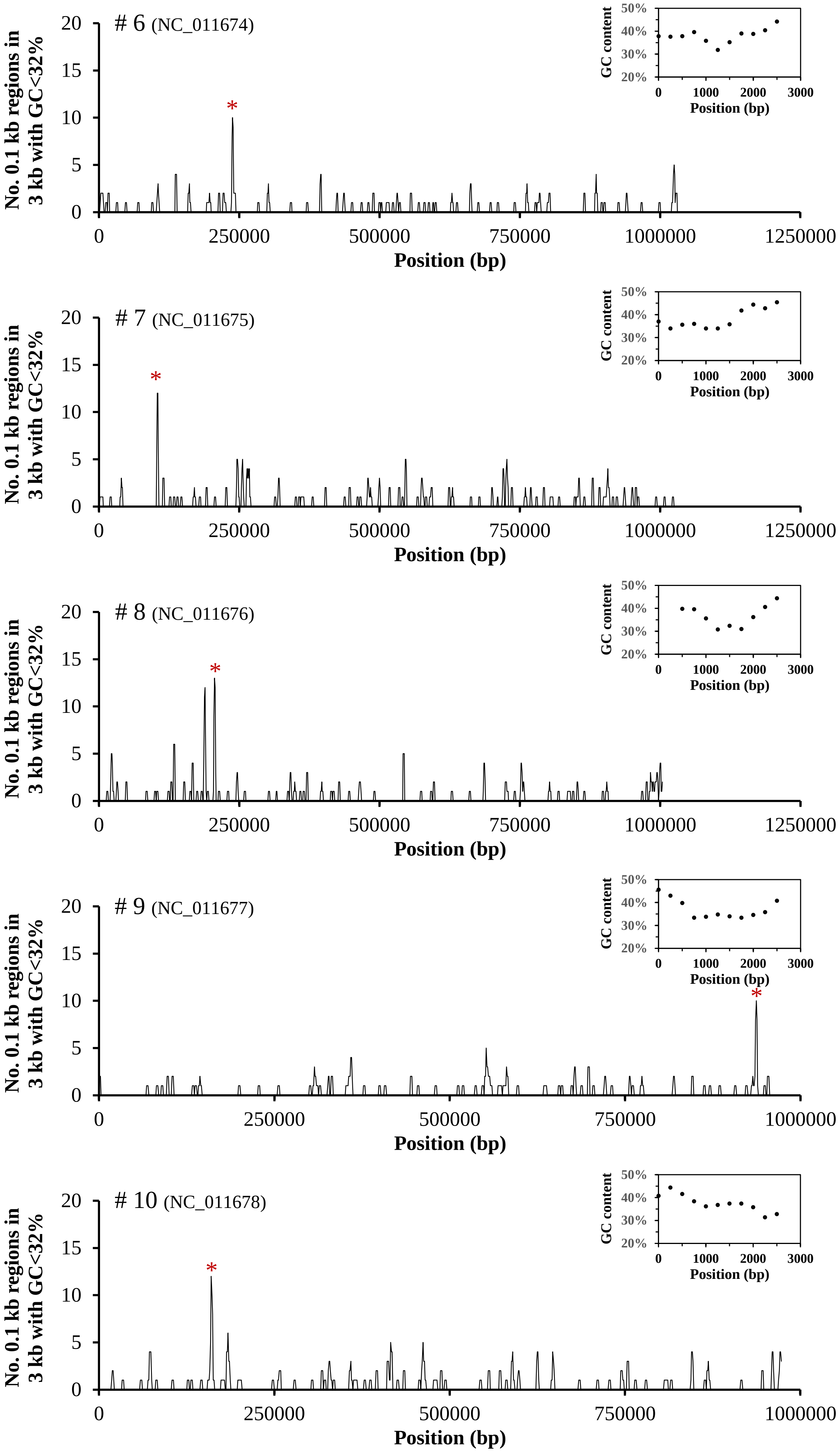

6 (NC_011674)
7 (NC_011675)
8 (NC_011676)
9 (NC_011677)
10 (NC_011678)
No. 0.1 kb regions in 3 kb with GC<32%
Position (bp)
GC content
*

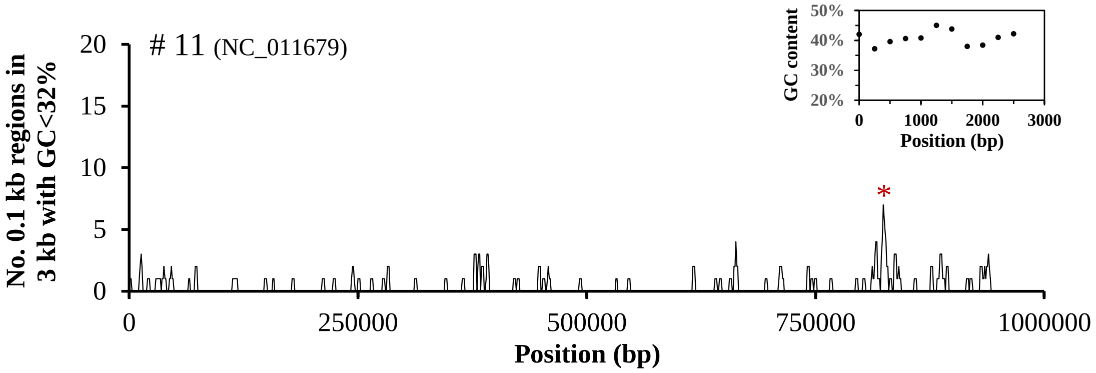

11 (NC_011679)
No. 0.1 kb regions in 3 kb with GC<32%
Position (bp)
GC content

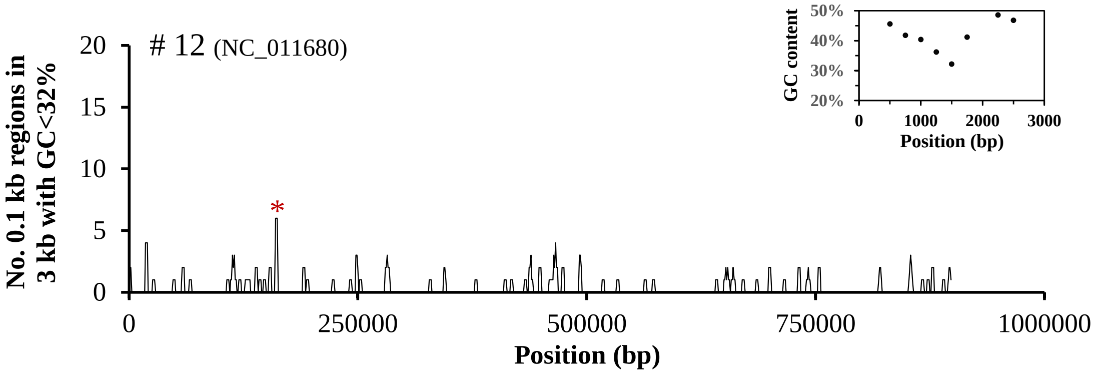

12 (NC_011680)
No. 0.1 kb regions in 3 kb with GC<32%
Position (bp)
GC content

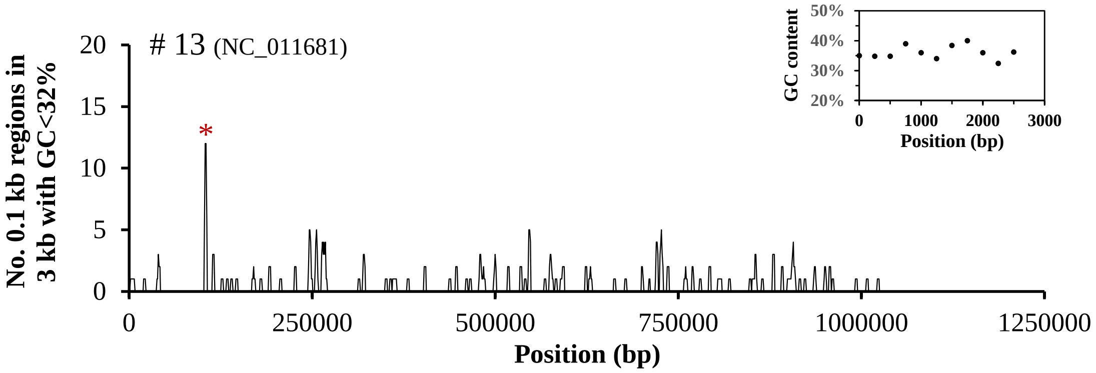

13 (NC_011681)
No. 0.1 kb regions in 3 kb with GC<32%
Position (bp)
GC content

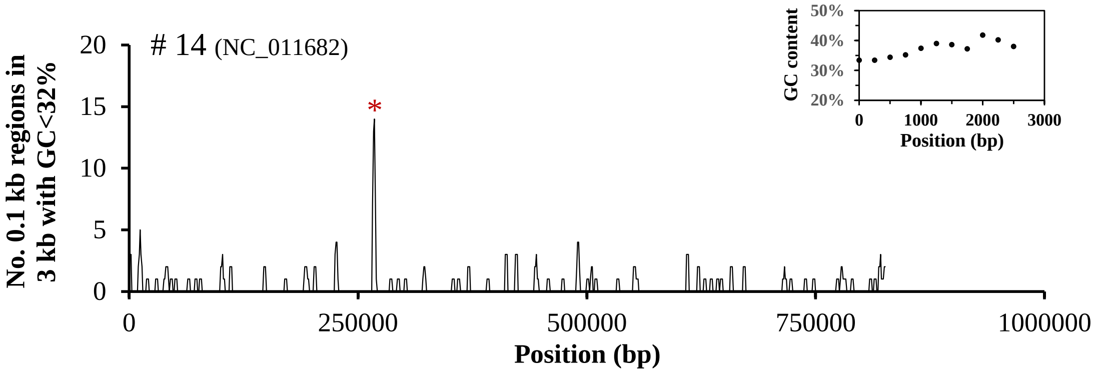

14 (NC_011682)
No. 0.1 kb regions in 3 kb with GC<32%
Position (bp)
GC content

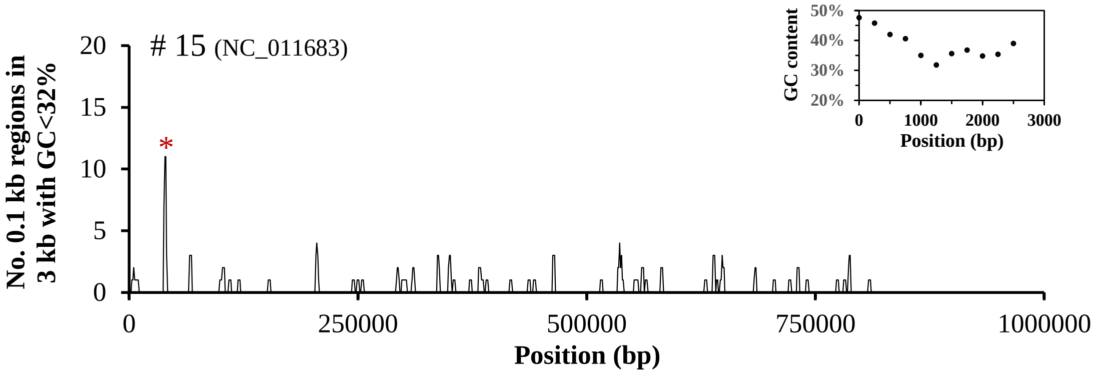

15 (NC_011683)
No. 0.1 kb regions in 3 kb with GC<32%
Position (bp)
GC content

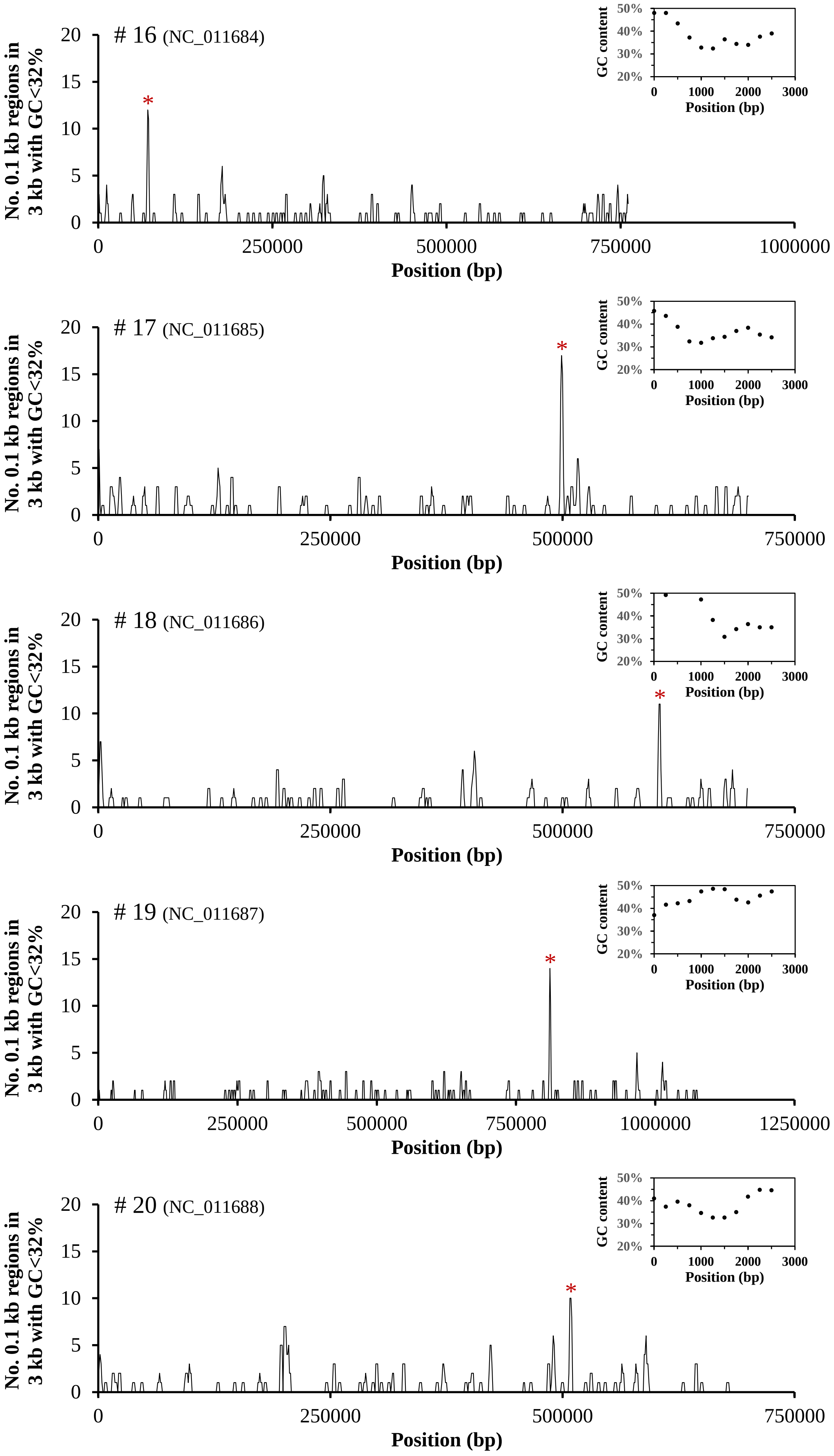

16 (NC_011684)
17 (NC_011685)
18 (NC_011686)
19 (NC_011687)
20 (NC_011688)
No. 0.1 kb regions in 3 kb with GC<32%
Position (bp)
GC content
50%
40%
30%
20%
0
1000
2000
3000
0
5
10
15
20
250000
500000
750000
1000000
1250000

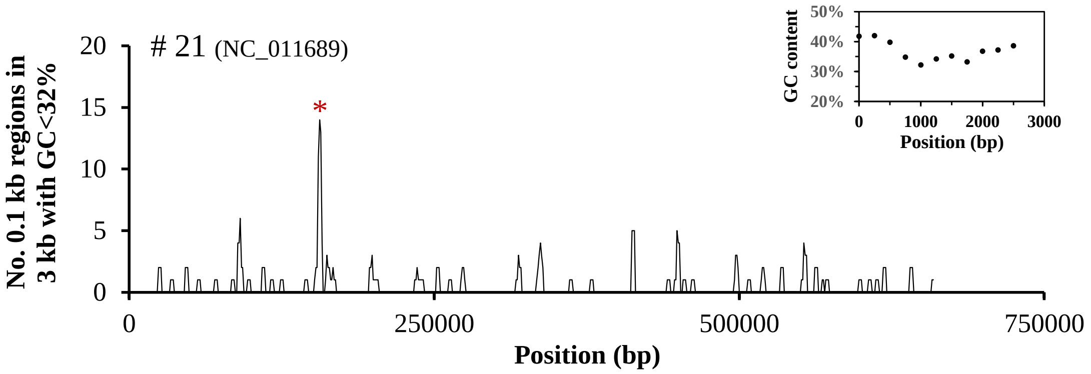

21 (NC_011689)
No. 0.1 kb regions in 3 kb with GC<32%
Position (bp)
GC content
Position (bp)

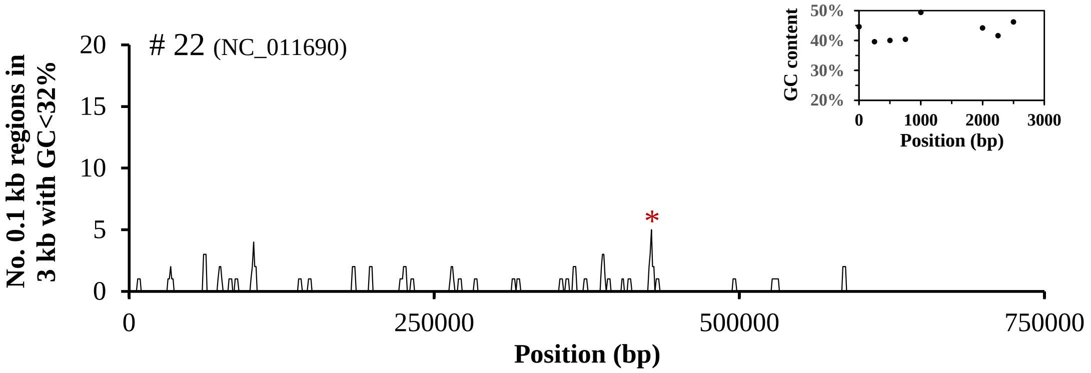

22 (NC_011690)
No. 0.1 kb regions in 3 kb with GC<32%
Position (bp)
GC content
Position (bp)

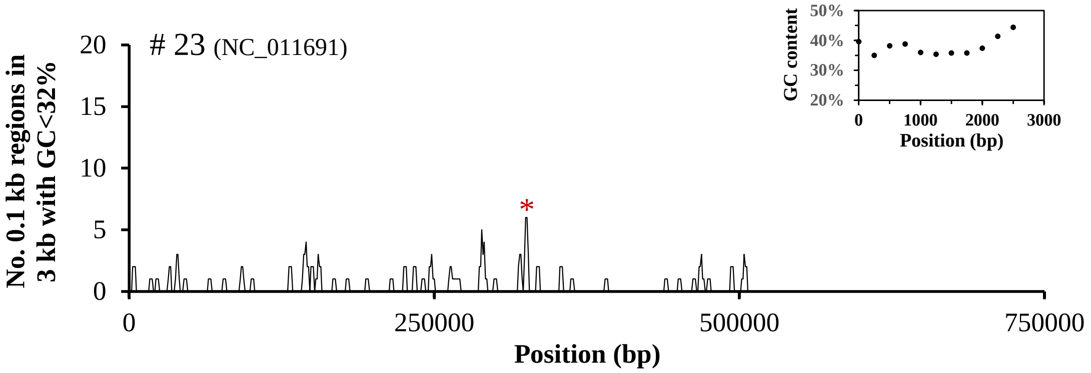

23 (NC_011691)
No. 0.1 kb regions in 3 kb with GC<32%
Position (bp)
GC content
Position (bp)

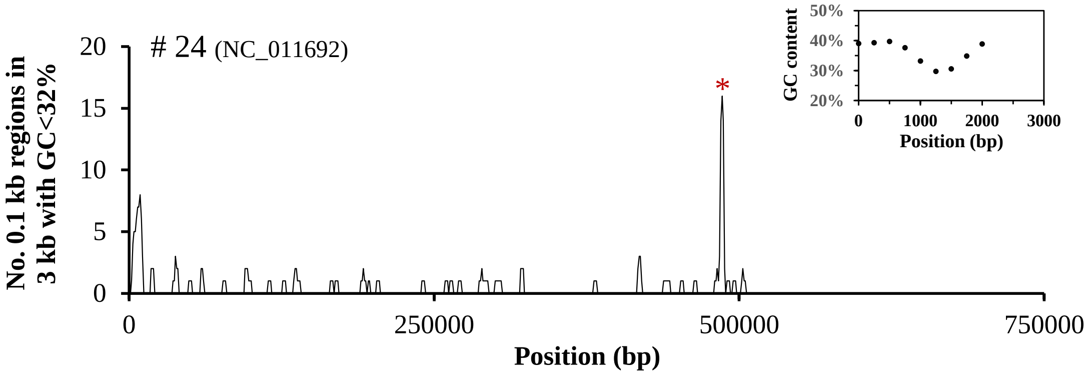

24 (NC_011692)
No. 0.1 kb regions in 3 kb with GC<32%
Position (bp)
GC content
Position (bp)

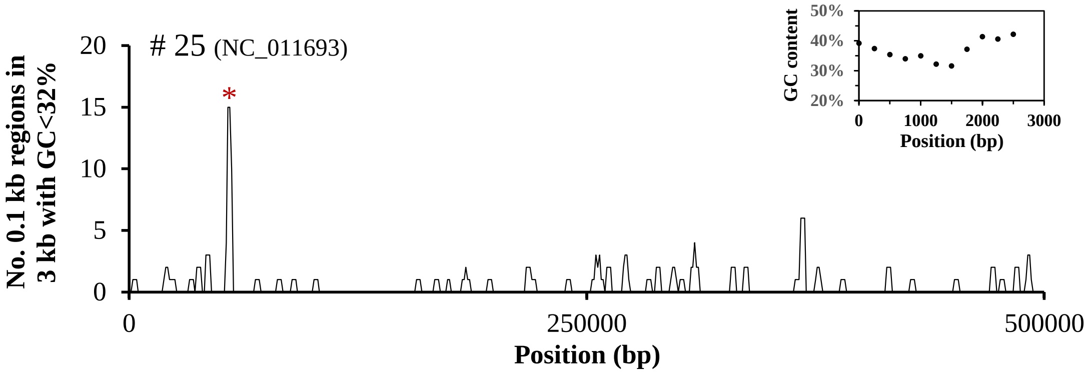

25 (NC_011693)
No. 0.1 kb regions in 3 kb with GC<32%
Position (bp)
GC content
Position (bp)

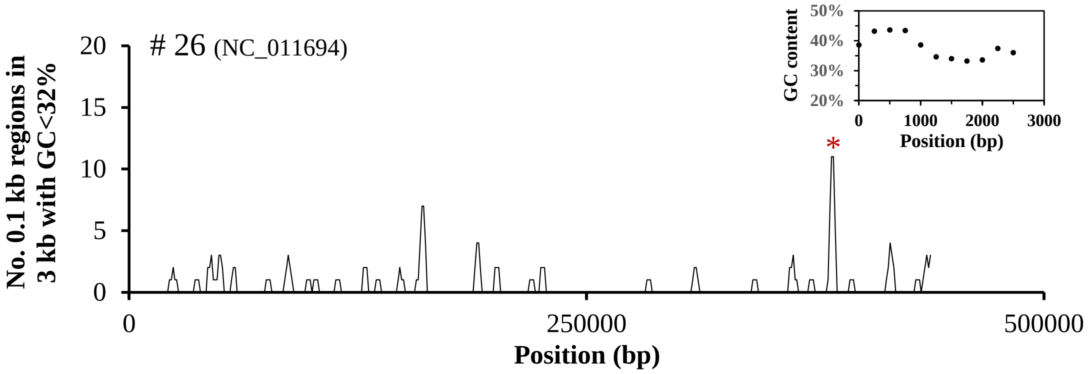

26 (NC_011694)
No. 0.1 kb regions in 3 kb with GC<32%
Position (bp)
GC content
*

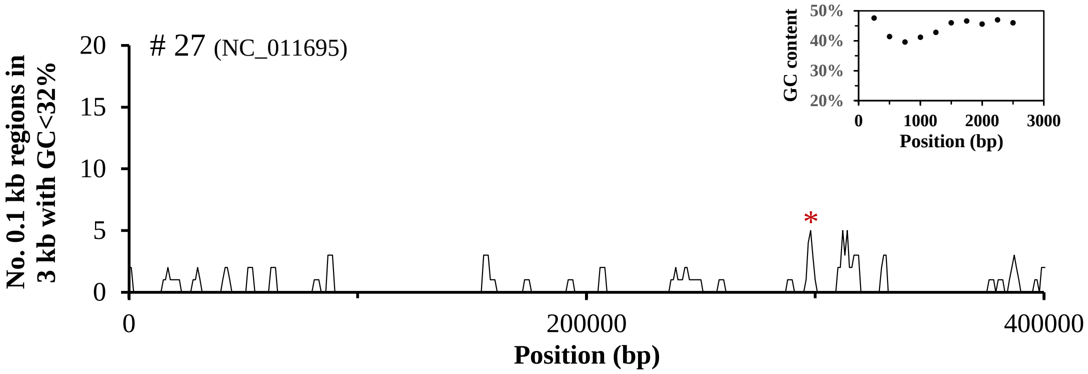

27 (NC_011695)
No. 0.1 kb regions in 3 kb with GC<32%
Position (bp)
GC content
*

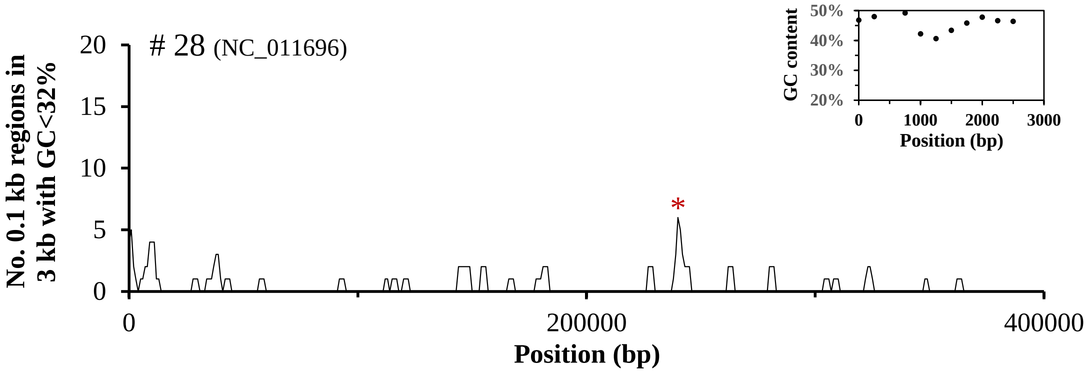

28 (NC_011696)
No. 0.1 kb regions in 3 kb with GC<32%
Position (bp)
GC content
*

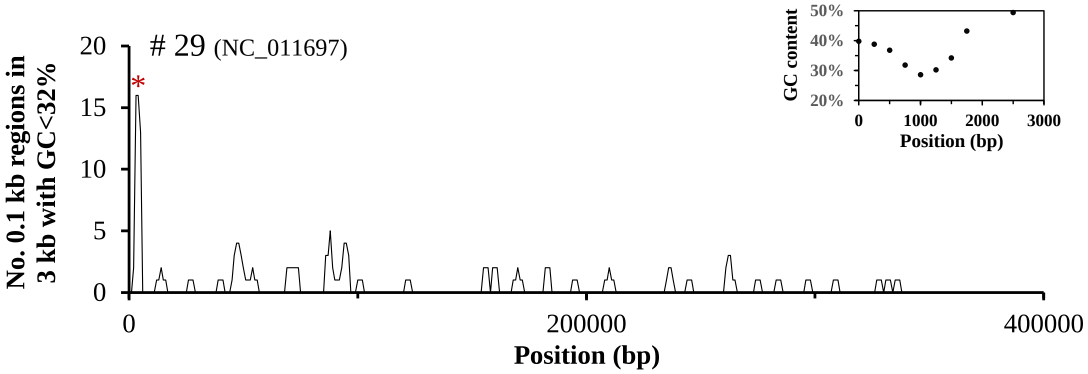

29 (NC_011697)
No. 0.1 kb regions in 3 kb with GC<32%
Position (bp)
GC content
*

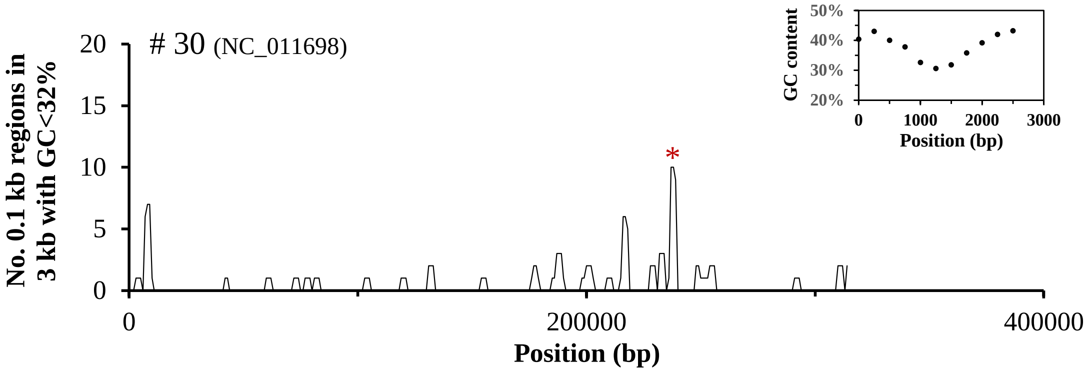

30 (NC_011698)
No. 0.1 kb regions in 3 kb with GC<32%
Position (bp)
GC content
*

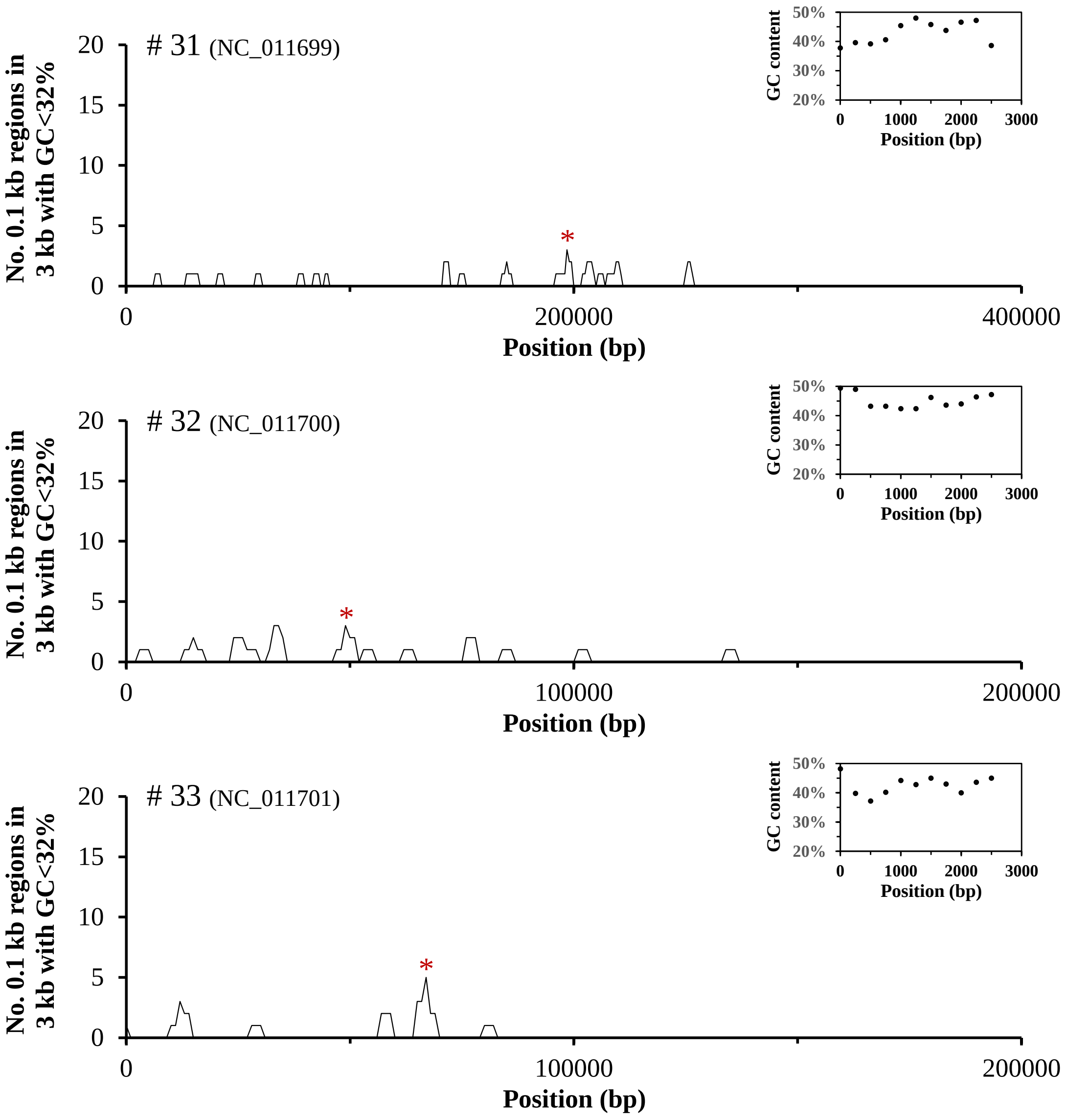

Supplemental Fig. S3 Predicted positions of centromeres on each chromosome of *P. tricornutum.* Chromosomal sequences of *P. tricornutum* CCAP 1055/1 were obtained from the NCBI dataset GCA_000150955.2. The analysis began with the identification of 100-bp regions (with a 50-bp overlap) within a 3-kb sliding window (advancing in 1-kb steps) with a GC content below 32%. The 3-kb region containing the highest number of such 100-bp windows was selected for further analysis. Within this region, the 500-bp sequence with the lowest GC content was identified.

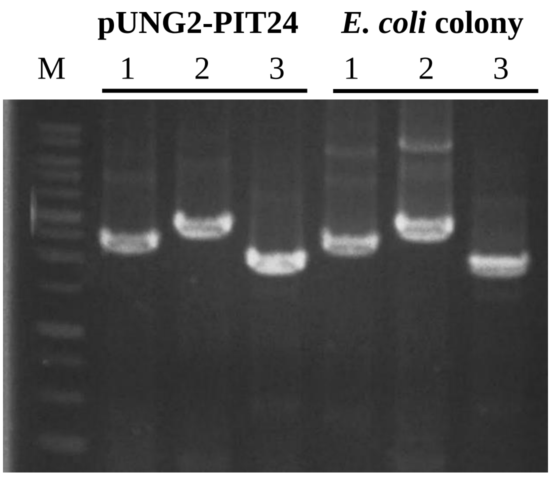

Supplemental Fig. S4 Results of colony PCR performed on transformed *E. coli* obtained from the rescue assay. The pUNG-PIT24 vector was used as a control group in the colony PCR comparison.

Supplemental Table S1 a PCR conditions. **b** primer sequences used in PCR analysis.

a)

Purpose	Primer set	PCR mixer		PCR condition		
		Component	Vol (µL)	Step	Time (m)	Temp (°C)
Preparing	ef1-(-1772)-*Not*I (F) &	DNA(100~500mg/L)	1	Initial denaturation	2.0	95
circular	ef1-2229-*Spe*I (R)	F primer (10 µM)	5	Denaturation	1.0	95
DNA	T&A-495-*Spe*I (F) &	R primer (10 µM)	5	Annealing } 32 cycles	0.30	55~60
constructs	T&A-170-*Not*I (R)	dNTP mix (10mM)	1	Extension	2.0-8.0	72
	N1-2629-*Nde*I (F) &	Pfu 10X buffer	5	Final extension	5.0	72
	N1-3423-*Bam*HI (R)	Pfu DNA	0.5			
	E1EE-2783-*Bam*HI (F) &	polymerase				
	E1EE-1780-*Nde*I (R)	H_2O	32.5			
	E1NE-1-*Not*I (F) &					
	E1NE-1478-*Nde*I (R)					
	gadph-t-1-*Bam*HI (F) &					
	gadph-t-978-*Spe*I (R)					
	18S-124-*Nde*I (F) &					
	18S-566-*Nde*I (R)					
	18S-NKNS-934 (F) &					
	18S-*Bam*HI-1536 (R)					
	yT&A-457-*Bam*HI (F) &					
	yT&A-182-*Nde*I (R)					
	Puc3-2479-*Nde*I (F) &					
	Puc3-3144-*Not*I (R)					
	E1NG1-18S-633-*Kpn*I (F) &					
	E1NG1-18S-1298-*Not*I (R)					
	ubi-(-762)-*Not*I (F) &					
	ubi-(-1)-*Nde*I (R)					
	UNG1-18S-641-*Not*I (F) &					
	UNG1-18S-2505-*Spe*I (R)					
Preparation of linear DNA constructs	18S(+) (F) & 18S(-) (R)	DNA(50~100mg/L)	1	Initial denaturation	3.0	95
	E (F) & G2 (R)	F primer (10 µM)	1.25	Denaturation	0.15	95
	U (F) & G2 (R)	R primer (10 µM)	1.25	Annealing } 32 cycles	0.15	55
		PCR Mix	12.5	Extension	3.0-5.0	72
		H_2O	9	Final extension	10.0	72
Checking centromeric plasmid in BL10	pE2NG1-18S-CA-2218 (F)+	DNA (50~100mg/L)	1	Initial denaturation	1.0	94
	pE2NG1-18S-CA-4376 (R)	F primer (10 µM)	1	Denaturation	0.5	98
	pE2NG1-18S-CA-5210 (F)+	R primer (10 µM)	1	Annealing } 32 cycles	0.5	55
	pE2NG1-18S-CA-6397 (R)	PCR Mix	25	Extension	0.2-1.3	72
	pE2NG1-18S-CA-3453 (F)+	H_2O	22	Final extension	3.0	72
	pE2NG1-18S-CA-3128 (R)					
	UNG2-CA-151 (F) &					
	UNG2-CA-3476 (R)					
	UNG2-CA-2513 (F) &					
	UNG2-CA-4435 (R)					
	UNG2-CA-3809 (F) &					
	UNG2-CA-731 (R)					
	UNG2-PIT24-151 (F) &					
	UNG2-PIT24-3310 (R)					
	UNG2-PIT24-3643 (F) &					
	UNG2-PIT24-3310 (R) UNG2-PIT24-2347 (F) &					
	UNG2-PIT24-4269 (R)					
	D-ef1α (F)					
	D-ef1α (R)					

Checking expression level of *neo* in BL10 (qRT-PCR)	neo-qPCR (F) & neo-qPCR (R) ef1a-qPCR (F) & ef1a-qPCR (R)	cDNA (100 mg/L)	1	Initial denaturation	3.0	95
		F primer (10 μM)	0.4	Denaturation } 40 cycles	0.3	95
		R primer (10 μM)	0.4	Anneal + Ext } 40 cycles	0.2	60
		Fast SYBR Green Master Mix (2X)	10	Melt curve anal.	0.15	95
					1.0	60
		H_2O	8.2		0.15	95
Colony PCR (Rescue assay)	UNG2-PIT24-1005 (F) & UNG2-PIT24-3310 (R) UNG2-PIT24-2347 (F) & UNG2-PIT24-4269 (R) UNG2-PIT24-3643 (F) & UNG2-PIT24-1280 (R)	*E.coli* cells	1	Initial denaturation	1.0	94
		F primer (10 μM)	1	Denaturation } 32 cycles	0.5	98
		R primer (10 μM)	1	Annealing } 32 cycles	0.5	55
		PCR Mix	25	Extension } 32 cycles	0.2-1.3	72
		H_2O	22	Final extension	3.0	72

b)

Usage	Primer name	Primer sequence (5′-3′)
Preparation of circular DNA constructs	ef1-(-1772)-*Not*I (F)	AAGCAGCGGCCGCGGAAAGCACCCCGAAGAAAAG
	ef1-2229-*Spe*I (R)	ACTAGTCACAGGCATTGGCAGGTTT
	T&A-495-*Spe*I (F)	TAAGCAACTAGTGGCGTAATCATGGTCATAGC
	T&A-170-*Not*I (R)	TGCTTAGCGGCCGCTACAATCTGCTCTGATGCCG
	N1-2629-*Nde*I (F)	GCATATGATTGAACAAGATGG
	N1-3423-*Bam*HI (R)	GGATCCTTAGAAGAACTCGTCAAGAAGGC
	E1EE-2783-*Bam*HI (F)	TAAGCAGGATCCGTGGTTTGACCTCTTATACTTGATC
	E1EE-1780-*Nde*I (R)	TGCTTACATATGTTCTTGATGAAATCACGGTGA
	E1NE-1-*Not*I (F)	ATAAGAATGCGGCCGCCTAGCTAGCT
	E1NE-1478-*Nde*I (R)	CGCCGGAATTCCATATGTTCTTGATGAA
	gadph-t-1-*Bam*HI (F)	CGCGGATCCATGTACCCAATA
	gadph-t-978-*Spe*I (R)	GCGGACTAGTACAAACTTGAAGC
	18S-124-*Nde*I (F)	CCGGAATTCCATATGATATGGATACC
	18S-566-*Nde*I (R)	GGAATTCCATATGCTTCTGGAGC
	18S-NKNS-934 (F)	CATATGGGTACCGCGGCCGCACTAGTCTGGGGATCG
	18S-*Bam*HI-1536 (R)	CGCGGATCCTGCAAAAATCTAGCCCCAGCACG
	yT&A-457-*Bam*HI (F)	TTATACGCGGATCCCCTCTAGAGTC
	yT&A-182-*Nde*I (R)	GCGGAATTCCATATGGTGCACTCTCAG
	Puc3-2479-*Nde*I (F)	CATATGGGGCCCGTTGCAGTCACTCCGCTTTGGTTTCA
	Puc3-3144-*Not*I (R)	GCGGCCGCGTGATAATGCCAATCGCTAAGAAAAAAAAAG
	E1NG1-18S-633-*Kpn*I (F)	GGTACCGTTGCAGTCACTCCGCTTTG
	E1NG1-18S-1298-*Not*I (R)	GCGGCCGCGTGATAATGCCAATCGCTAA
	ubi-(-762)-*Not*I (F)	ATTTATAGCGGCCGCCACAGTTACTCCGAGTC
	ubi-(-1)-*Nde*I (R)	GGGCCCACATATGGTTGTTTGCTTATCTA
	UNG1-18S-641-*Not*I (F)	GCGGCCGCGGGAGACTTTGAAAGGCACAG
	UNG1-18S-2505-*Spe*I (R)	ACTAGTTTTGTGAATGAAAA
Preparation of linear DNA constructs	18S(+) (F)	CCTGCAGTAATTCTGGAAATAATACATGCTG
	18S(-) (R)	TGCAAAAATCTAGCCCCAGCACG
	E (F)	CTAGCTAGCTCATGCTCCTTTCCCG
	U (F)	GGGAGACTTTGAAAGGCACA
	G2 (R)	ACTAGTTTTGTGAATGAAAAGAGA
Confirming centromeric plasmid in BL10	pE2NG1-18S-CA-2218 (F)	GTGCCACAGCAGACCAGTAA
	pE2NG1-18S-CA-4376 (R)	TGCAAATGTTGACGATGAGC
	pE2NG1-18S-CA-5210 (F)	AGCTTGGCGTAATCATGGTC
	pE2NG1-18S-CA-6397 (R)	GCATTGGTAACTGTCAGACCAA
	pE2NG1-18S-CA-3453 (F)	CGTTGGCTACCCGTGATATT
	pE2NG1-18S-CA-3128 (R)	ATACTTTCTCGGCAGGAGCA
	UNG2-CA-151 (F)	CGGCATCAGAGCAGATTGTA
	UNG2-CA-3476 (R)	TTTTTGTGATGCTCGTCAGG
	UNG2-CA-2513 (F)	CGTTGGCTACCCGTGATATT
	UNG2-CA-4435 (R)	GGATGGAGGCGGATAAAGTT
	UNG2-CA-3809 (F)	GCAGAGCGAGGTATGTAGGC
	UNG2-CA-731 (R)	CACAGGATTTTCGTGTGTGG
	UNG2-PIT24-1005 (F)	ATCCTGGCAGACATGTGGAA
	UNG2-PIT24-3310 (R)	TTTTTGTGATGCTCGTCAGG
	UNG2-PIT24-2347 (F)	CGTTGGCTACCCGTGATATT
	UNG2-PIT24-4269 (R)	GGATGGAGGCGGATAAAGTT
	UNG2-PIT24-3643 (F)	GCAGAGCGAGGTATGTAGGC
	UNG2-PIT24-1280 (R)	CGTCTGTTCTCTCTGCCTCT
	UNG2-PIT24-151 (F)	CGGCATCAGAGCAGATTGTA

	D-ef1α (F)	CTTGCTTGCCGTATGCTC
	D-ef1α (R)	GGTGGTGGTGGACTTGC
Checking expression level of *neo* in BL10	neo-363 (F)	GCGGCTGCATACGCTTGATCC
	neo-457 (R)	CAAGACCGGCTTCCATCCGAG
	ef1α-616 (F)	GACATCAAGCGCGGTAAC
	ef1α- 786 (R)	GATCTCGGCGAACTTGC

Supplemental Table S2 Formula of culture media

M3 medium

Ingredients	Unit
Peptone	0.5 g
Yeast extract	1.0 g
Glucose	2.0 g
Nutrient solution	500 μL
Vitamin solution	50 μL
Agar (if needed)	8 g
Sea salt	32 g
Distilled water	to 1 L

Nutrient solution

Ingredients	Unit
Ferric chloride hexahydrate	1.3 g
Manganese chloride tetrahydrate	0.36 g
Boric acid	33.6 g
EDTA	45.0 g
Sodium dihydrogen phosphate dihydrate	20.0 g
Sodium nitrate	100.0 g
Distilled water	to 1 L

Vitamin solution

Ingredients	Unit
Vitamin B1	2.0 g
Vitamin B12	0.1 g
Vitamin H	0.1 g
Distilled water	to 1 L

H3 medium

Ingredients	Unit
Peptone	1.0 g
Yeast extract	2.0 g
Glucose	4.0 g
Seawater 28‰	to 1 L

LB medium

Ingredients	Unit
LB broth	25.0 g
Agar (if needed)	15 g
Distilled water	to 1 L

GYSS medium

Ingredients	Unit
Glucose	45.0 g
Yeast extract	4.5 g
Sodium sulfate	9.0 g
Seawater 1‰	to 1 L

2X GYSS medium

Ingredients	Unit
Glucose	90.0 g
Yeast extract	9.0 g
Sodium sulfate	9.0 g
Seawater 1‰	to 1 L
